# BET proteolysis targeted chimera-based therapy of novel models of Richter Transformation-diffuse large B-cell lymphoma

**DOI:** 10.1038/s41375-021-01181-w

**Published:** 2021-03-02

**Authors:** Warren Fiskus, Christopher P. Mill, Dimuthu Perera, Christine Birdwell, Qing Deng, Haopeng Yang, Bernardo H. Lara, Nitin Jain, Jan Burger, Alessandra Ferrajoli, John A. Davis, Dyana T. Saenz, Wendy Jin, Cristian Coarfa, Craig M. Crews, Michael R. Green, Joseph D. Khoury, Kapil N. Bhalla

**Affiliations:** 1 The University of Texas M.D. Anderson Cancer Center, Houston, TX USA; 2 Department of Molecular and Cellular Biology, Baylor College of Medicine, Houston, TX USA; 3 Department of Molecular, Cellular, and Developmental Biology, Yale University, New Haven, CT USA; 4 Department of Chemistry, Yale University, New Haven, CT USA; 5 Department of Pharmacology, Yale University, New Haven, CT USA

**Keywords:** B-cell lymphoma, Targeted therapies

## Abstract

Richter Transformation (RT) develops in CLL as an aggressive, therapy-resistant, diffuse large B cell lymphoma (RT-DLBCL), commonly clonally-related (CLR) to the concomitant CLL. Lack of available pre-clinical human models has hampered the development of novel therapies for RT-DLBCL. Here, we report the profiles of genetic alterations, chromatin accessibility and active enhancers, gene-expressions and anti-lymphoma drug-sensitivity of three newly established, patient-derived, xenograft (PDX) models of RT-DLBCLs, including CLR and clonally-unrelated (CLUR) to concomitant CLL. The CLR and CLUR RT-DLBCL cells display active enhancers, higher single-cell RNA-Seq-determined mRNA, and protein expressions of IRF4, TCF4, and BCL2, as well as increased sensitivity to BET protein inhibitors. CRISPR knockout of IRF4 attenuated c-Myc levels and increased sensitivity to a BET protein inhibitor. Co-treatment with BET inhibitor or BET-PROTAC and ibrutinib or venetoclax exerted synergistic in vitro lethality in the RT-DLBCL cells. Finally, as compared to each agent alone, combination therapy with BET-PROTAC and venetoclax significantly reduced lymphoma burden and improved survival of immune-depleted mice engrafted with CLR-RT-DLBCL. These findings highlight a novel, potentially effective therapy for RT-DLBCL.

## Introduction

Richter Transformation (RT) is defined as the development of aggressive DLBCL (mostly ABC-type) in up to ~15% of patients with antecedent or concurrent diagnosis of chronic lymphocytic leukemia (CLL) [1, 2]. Based on the comparison of immunoglobulin gene rearrangements, ~80% of RT-DLBCLs arise due to a direct clonal evolution of the underlying CLL clone, i.e., clonally related (CLR) RT-DLBCLs, which exhibit poor median survival of 1 year [1–3]. Alternatively, ~20% of RT-DLBCLs are clonally unrelated (CLUR) to the underlying CLL, arising most likely due to branched clonal evolution from a common pre-CLL progenitor [2–4]. CLUR RT-DLBCLs exhibit a better median survival of 5 years [1–3]. Among the genetic features in CLL that predispose to RT include a stereotypic BCR (subset 8), deletion 17p13, as well as CD38 and LRP4 polymorphisms [5]. There is also an increased risk of RT-DLBCL in CLL with NOTCH1 mutations, bulky lymphadenopathy, and on PET-CT SUV > 5 in tumor masses, but the risk of developing RT-DLBCL is unrelated to any specific prior therapy of CLL [1, 2, 5, 6]. RT-DLBCL is associated with recurrent genetic alterations in TP53 (~60%), CDKN2A (~50%), NOTCH1 (~30%) and MYC (~40%), as well as cytogenetic alterations in 17p, 9p21, trisomy 12, loss of 13q14.3, 7q31–36.3, 11q22, 14q23.2-q32.33, and near tetraploidy [2, 3, 7–9]. RT-DLBCLs are Epstein-Barr virus (EBV) negative and commonly express high levels of CD20, CD23, PD-1, and PAX5, with low expression of CD5 and CD10 [2, 5, 10]. Clinical studies have documented that chemo-immunotherapy, or treatment with the Bruton’s tyrosine kinase (BTK) inhibitor ibrutinib, anti-apoptotic BCL2 inhibitor venetoclax, or with anti-PD1 checkpoint blockade therapy fails to achieve prolonged disease-free survival, and majority of patients relapse with therapy-refractory disease [2, 6, 11–15]. This poor clinical outcome highlights the unmet need to develop and test novel targeted therapies for RT-DLBCL. To achieve this, novel in vitro cellular and in vivo patient-derived xenograft (PDX) models of CLR and CLUR RT-DLBCL are essential for elucidating therapeutic targets and for developing and testing novel targeted therapies.

Constitutive activation of B-cell receptor (BCR) signaling and of downstream transcription factors (TFs), especially c-Myc and NFkB, contribute to the growth and survival of lymphoma, including RT-DLBCL cells [2, 3, 16–18]. These TFs bind to their canonical DNA binding sites on enhancers and promoters, recruiting HATs (histone acetyltransferases) to induce acetylation of lysine residues on histone H3 and H4 proteins and TFs [19–21]. As a member of the bromodomain extra-terminal (BET) family of reader proteins (BETP), BRD4 binds to acetylated lysine residues on histone proteins and TFs and assembles a complex of co-regulatory proteins [19–21]. These include mediator protein and P-TEFb (a heterodimer composed of CDK9 and its regulatory subunit Cyclin T) at super enhancers (SEs)/enhancers (Es) and promoters [19–21]. The kinase activity of CDK9 in P-TEFb phosphorylates serine-2 in the heptad-repeats in the C-terminal domain (CTD) of RNAP2, as well as phosphorylates the negative elongation factors NELF and SPT5, which induces promoter proximal pause-release of RNAP2 to enable productive mRNA transcript elongation [19–23]. Thus, BRD4-CDK9 axis induces RNAP2-mediated transcription of SE/E-driven oncogenes, including MYC, BCL2, Bcl-xL and CDK4/6, which are important for cell growth and survival of RT-DLBCL cells [19, 24, 25]. BRD4 also binds acetylated RELA (NFkB-p65) and is essential for NFkB transcriptional activity, necessary for survival of lymphoma cells [26, 27]. MYC is transcriptionally activated downstream of NOTCH1 mutation, whereas CDKN2A mutation and loss creates a dependency on CDK4 activity through RB phosphorylation [2, 5, 17]. Recently we had also reported that, in ABC-DLBCLs, copy gains of TCF4 gene are common, and BRD4 targeting inhibits TCF4 levels and induces apoptosis in ABC-DLBCL cells [28]. Taken together, these observations suggest that inhibition of BRD4 levels/activity could potentially undermine the transcriptional networks that sustain the growth and survival of RT-DLBCL cells.

In present studies, to address the lack of availability of in vitro cellular and in vivo PDX models of RT-DLBCL, which could be utilized for pre-clinically evaluating the efficacy of novel targeted agents, we successfully established three patient-derived xenograft (PDX) models of RT-DLBCL cells, including clonally related (CLR) (HPRT3) and clonally unrelated (CLUR) (HPRT2) to antecedent CLL. The third RT-DLBCL was a rarer GCB variety of RT-DLBCL (HPRT1). In a previous report, clonal relationships of the RT-DLBCL PDX models to preceding CLL and their sensitivity to therapeutic agents was not characterized [29]. The RT-DLBCL cells for the three PDX models presented here were derived from 3 separate patients with histologically-documented RT-DLBCL developing in CLL. The PDX models were established in immune-depleted NSG mice, after tail-vein infusion and engraftment of CD19+ RT-DLBCL cells. Studies presented here also describe the genetic alterations, as well as characterize the epigenomic and transcriptional features of the three PDX models. Additionally, present studies demonstrate marked dependency of CLR, RT-DLBCL cells on BRD4-regulated enhancers of oncogenes, including TCF4, IRF4, and MYC. They also demonstrate synergistic lethal activity of BET-proteolysis targeting chimera-based combinations with ibrutinib and venetoclax.

## Materials and methods

### Cell lines and cell culture

Human Richter Transformation DLBCL cells (harvested from the spleen, bone marrow, and liver of PDX-bearing mice) were cultured in RPMI media with 20% heat-inactivated fetal bovine serum (FBS), 1% penicillin/streptomycin and 1% non-essential amino acids. Following drug treatments, cells were washed free of the drug(s) prior to performing the studies described.

### Flow cytometry analysis of cell surface markers on RT-DLBCL cells

To determine the immunophenotype of the RT-DLBCL cells, HPRT3, HPRT2, and HPRT1 cells were harvested from NSG mice. Cells were suspended in 100 µL of 0.5% BSA/PBS and stained with fluorophore-conjugated anti-CD19, anti-CD5, anti-CD10, anti-CD20, anti-CD23, anti-PD-1 or IgG-isotype controls (BD Biosciences, San Jose, CA). Percent expression of each cell surface marker is reported relative to the respective IgG isotype control.

### RNA isolation and quantitative polymerase chain reaction

Following the designated treatments with ARV-771 or OTX015, total RNA was isolated from RT-DLBCL cells utilizing a PureLink RNA Mini kit from Ambion, Inc. and reverse transcribed. Quantitative real-time PCR analysis was performed on cDNA using TaqMan probes from Applied Biosystems (Foster City, CA).

### Single-cell RNA-Seq analysis of RT-DLBCL cells

To determine baseline expression of mRNA in the RT-DLBCL cells, we performed single cell RNA-Seq analysis utilizing the 10× Genomics Chromium Separator and a Chromium™ Single Cell 3′ Solution kit followed by next-generation sequencing (NGS). Sequencing files were loaded into Cell Ranger and Loupe Cell Browser for clustering, visualization, and analysis.

### Statistical analysis

Significant differences between values obtained in HPRT3, HPRT2, or HPRT1 cells treated with different experimental conditions were determined using the Student’s *t*-test in GraphPad V8. For the in vivo mouse models, a two-tailed *t*-test or a Mantel–Cox Rank sum test was utilized for group comparisons. *P* values of <0.05 were assigned significance.

### Data sharing statement

RNA-Seq, ATAC-Seq, and ChIP-Seq datasets have been deposited in GEO as a Super Series under accession # GSE154463.

Detailed methods for transcriptome analysis, next-generation sequencing (NGS) of RT-DLBCL cells by L-300 liquid panel, analysis of epigenetic state in RT-DLBCL cells, CRISPR/Cas9-mediated gene editing in RT-DLBCL cells, confocal immunofluorescence microscopy, and RT-DLBCL xenograft studies are provided in the Supplemental Methods.

## Results

### Generation and biologic features of three PDX models of RT-DLBCLs

**Fig. 1:**
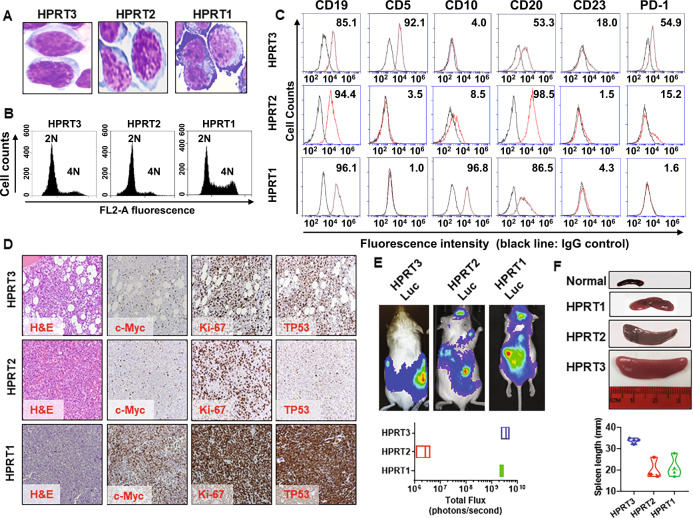
Phenotypic characterization of human HPRT3, HPRT2, and HPRT1 Richter Transformation PDX models. **A** RT-DLBCL cells from PDX models were cytospun onto glass slides at 500 rpm utilizing a cyto-centrifuge. Cells were fixed with Fast Green and stained with hematoxylin and eosin. Original magnification is ×40. Cell images were obtained with a CCD camera mounted on a microscope. **B** Representative cell cycle status histograms of HPRT3, HPRT2, and HPRT1 cells. RT-DLBCL cells were harvested, washed with 1× PBS and fixed in 70% molecular grade ethanol overnight at −20 °C. Following this, RT-DLBCL cells were washed with 1× PBS and stained with 1 mg/mL propidium iodide in Triton-PBS buffer. Cells were analyzed by flow cytometry. **C** RT-DLBCL cells were harvested and stained with the indicated cell surface markers or the corresponding IgG isotype control. The numbers beside the histograms indicate the % of cells expressing each surface marker relative to the IgG isotype control. **D** Immunohistochemical evaluation of c-Myc, KI-67, and TP53 expression in RT cells. **E** HPRT3, HPRT2, and HPRT1 cells were luciferized to enable tracking of their in vivo growth in NSG mice. Representative images of engrafted luciferase-expressing RT-DLBCL PDX cells 4 weeks after cell implantation are shown. The total flux (photons/sec) from 2 to 3 mice with each PDX is shown. **F** Representative spleens from HPRT1, HPRT2, and HPRT3-engrafted mice compared to a normal mouse spleen approximately 6 weeks after cell implantation. The range of spleen sizes from 3 mice with each PDX model is shown in the violin plot.

CD19-expressing RT-DLBCL HPRT3, HPRT2, and HPRT1 cells were purified from the core biopsy samples from three patients with histologically-documented RT-DLBCL developing in CLL. Prior to establishing their PDX models, we first characterized the biologic features of the RT-DLBCL cells. Figures 1A–C and S1A, respectively, present the morphologic features, cell cycle phase-distribution, and cell-surface markers of the RT-DLBCL cells. Compared to HPRT3 cells, HPRT2 and HPRT1 cells expressed low CD5, CD23, and PD1, but higher expression of CD20 (Fig. 1C). Flow cytometry and immunohistochemistry analyses revealed that HPRT1 cells exhibited higher % of cells in the S and G2/M phases of the cell cycle and greater expression of Ki-67, TP53, and c-Myc (Figs. 1B, D and S1A). FISH analysis confirmed 5′ MYC amplification but 3′ MYC deletion in HPRT1 cells (Fig. S1B). HPRT3 and HPRT2 cells were of the most common ABC-DLBCL variety of RT-DLBCL, based on positive MUM/IRF4 and negative CD10 and BCL6 expressions [1, 2]. In contrast, HPRT1 cells displayed high CD10 and BCL6 expressions, consistent with the rare GCB-DLBCL sub-type of RT-DLBCL (Figs. 1C and S1C). In a large cohort of additional 52 RT-DLBCLs managed at MD Anderson Cancer Center, co-expression of CD10 and BCL6 was documented by immunohistochemistry in only one sample, whereas PAX5 and IRF4 were expressed in virtually all RT-DLBCL samples (Tables S1 and S2). All three RT-DLBCL cell-types lacked EB virus DNA or the expression of EBNA2 protein (Fig. S1D, E) [2]. Clonal relationship of each of the three RT-DLBCL samples to their antecedent CLL samples was assessed by analyzing and comparing their immunoglobulin genes to those of the preceding CLL cells. Notably, HPRT3 was clonally-related to its antecedent CLL, whereas HPRT2 was clonally-unrelated (Table S3). Clonal relationship of HPRT1 cells to its antecedent CLL could not be established because of the lack of availability of the DNA from the preceding CLL cells. Next, we established PDX models of luciferase-transduced, CD19+ HPRT3, HPRT2, and HPRT1 cells, following tail-vein infusion and engraftment in immune-depleted NSG mice (Fig. 1E). The RT-DLBCL cells grew in the bone marrow, spleen and liver and caused marked splenomegaly and hepatomegaly, requiring euthanasia of the mice 4 to 6 weeks after engraftment (Fig. 1F).

#### Cytogenetics, gene-copy number variations, and genetic mutations in the RT-DLBCLs

Cytogenetic analysis showed large numbers of karyotypic abnormalities in the three DLBCLs, especially pronounced in HPRT3 cells (Fig. S2A). Chromosomes showing tetrasomies were prevalent in HPRT3, whereas trisomies were present in HPRT3 and HPRT2 RT-DLBCLs (Fig. S2A). Array-CGH analysis also demonstrated gains and losses of chromosomal regions, again more pronounced in HPRT3 compared to HPRT2 and HPRT1 cells (Fig. S2B). We also conducted low-pass whole-genome sequencing to determine regions of amplification and losses [28]. All three RT-DLBCLs showed large areas of DNA copy gains and losses in their chromosomes (Fig. S2C). HPRT3 and HPRT2, but not HPRT1, cells showed large areas of amplification of DNA on chromosome 18, with copy gains at 18q21.1 involving the TCF4 gene (Fig. S2C). This is consistent with our reported findings that DNA copy gains of TCF4 gene are common in ABC-like DLBCL [28]. To detect genetic variants, we also performed next-generation sequencing (NGS) of whole exomes of a panel of 300 genes (L-300 panel) (Table S4). Consistent with the pronounced aneuploidy observed in HPRT3 over HPRT2 cells noted above, as shown in Tables S5–S7, a greater number of genetic alterations at high % variant allelic frequency (VAF) were detected in HPRT3, as compared to HPRT2 or HPRT1 cells. These genetic alterations targeted transcription factors, epigenetic regulators, DNA damage/repair enzymes, signaling enzymes, and their regulators.

### Epigenomic and gene-expression diversity of the RT-DLBCL cells

**Fig. 2:**
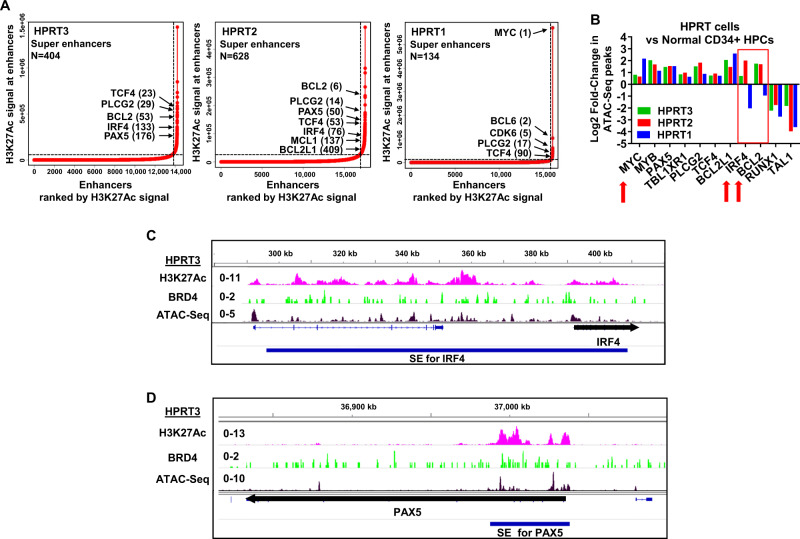
Epigenome and super-enhancer analysis in HPRT3, HPRT2, and HPRT1 cells and sequence tag density plots of H3K27Ac and BRD4 occupancy and chromatin accessibility at the IRF4 and PAX5 genes in HPRT3-DLBCL cells. **A** H3K27Ac ChIP-Seq analysis was performed on the chromatin from RT-DLBCL cells. Ranked ordering of SE (ROSE) analysis was performed utilizing the H3K27Ac sequence-tag density and H3K27Ac signal at enhancers. **B** ATAC-Seq analysis was performed on the nuclei from RT-DLBCL cells to determine chromatin accessibility. Publicly available ATAC-Seq data from normal CD34+ HPCs were downloaded from GEO. Log2 fold-changes in peaks from selected RT-relevant genes as determined by ATAC-Seq analysis are shown. Red arrows indicate significant differences in accessibility in the three RT-DLBCL subtypes compared to each other and normal CD34+ cells. **C**, **D** Integrated Genome Viewer (IGV) plots showing H3K27Ac and BRD4 ChIP-Seq signal density and ATAC-Seq determined chromatin accessibility at the IRF4 and PAX5 genes in clonally-related HPRT3-DLBCL cells. The blue bars beneath the signal tracks indicate the position of the super enhancer for IRF4 and PAX5 as determined by ROSE analysis.

To elucidate the impact of the large array of genetic alterations on chromatin accessibility, enhancer activity, and on gene-expression profiles, we performed ATAC-Seq, ChIP-Seq with H3K27Ac and BRD4 antibodies, and single-cell (sc) RNA-Seq analyses in HPRT3, HPRT2, and HPRT1 cells [28, 30, 31]. Utilizing anti-H3K27Ac ChIP-Seq analysis, we evaluated the active chromatin as signal-density plots of H3K27Ac mark on the chromatin of RT-DLBCL cells compared to publicly available H3K27Ac ChIP-Seq data from normal CD34+ hematopoietic progenitor cells (HPCs) (GSM772870, GSM772885, and GSM772894). Fig. S3A shows the average, normalized read-density across all SEs/Es as markedly increased sequence-tag densities in HPRT3 > HPRT1 > HPRT2 > normal CD34+ HPCs, compared to the read-densities on either side of the SEs/Es. Taking into account the SE/E score reflecting both enhancer-size and density of reads, we identified several SEs with high scores in HPRT3, HPRT2, and HPRT1 cells, as shown in the ‘ROSE plots’ in Fig. 2A. The top 25 super enhancers in HPRT3, HPRT2, and HPRT1 are shown in Table S8A–C. Whereas SEs of TCF4 and PLCG2 scored high in all three RT-DLBCL cells, MYC, BCL6, and CDK6 SEs scored high only in HPRT1 cells [28, 32, 33]. HPRT3 and HPRT2 also demonstrated active SEs of BCL2, PAX5, and IRF4 (Fig. 2A) [25, 32–34]. Heat map of the signal density determined by ATAC-Seq analysis showed markedly altered signal intensity in the three RT-DLBCL cell-types, as compared to publicly-available ATAC-Seq data from normal CD34+ HPC cells (GSE18927) (Fig. S3B). Loci showing log2 fold-alterations in ATAC-Seq peaks in HPRT3, HPRT2, and HPRT1 cells, versus normal CD34+ HPC ATAC-Seq peaks, are depicted in Fig. 2B. Compared to HPRT3 and HPRT2 cells, chromatin of IRF4 and BCL2 genes was less, whereas that of MYC was more accessible in HPRT1 cells (Fig. 2B). Figures 2C, D and S3C, D demonstrate ChIP-Seq analyses-determined signal-density plots of H3K27Ac and BRD4, as well as ATAC-Seq-determined peak-density at the MYC and the adjacent PVT1 gene [31], as well as at the SEs for IRF4, TCF4, and PAX5 genes. As shown here, in HPRT3 cells, greater H3K27Ac and BRD4 peaks and increased chromatin accessibility was observed on the E of PVT1, as well as on the SEs of TCF4, IRF4, and PAX5 genes. Similar findings were also observed in HPRT2 cells (Fig. S3E–H). In HPRT1 cells, in addition to MYC gene amplification, the Es of MYC and PVT1 genes showed a marked increase in H3K27Ac and BRD4 peaks and augmented chromatin accessibility, especially at the Es 1, 2, and 3 for the MYC gene (Fig. S3I). Next, we performed single-cell (sc) RNA sequencing, and generated t-SNE plots from the sequence reads based on similar mRNA expression levels in the clustered HPRT3 (12 clusters), HPRT2 (7 clusters) and HPRT1 cells (7 clusters) (Fig. S4A–C). Panels below the t-SNE plots show the number of clusters and number of cells per cluster for HPRT3, HPRT2, and HPRT1 cells. Fig. S4D demonstrates selected t-SNE plots of the baseline mRNA expressions of TCF4, MYC, IRF4, PAX5, BCL2, and BCL2L1 across all clusters in HPRT3, HPRT2, and HPRT1 cells. As shown here, whereas TCF4 expression was high in all clusters in all three RT-DLBCLs, MYC mRNA was clearly expressed at high levels in HPRT1 cells. Additional t-SNE plots of baseline mRNA expressions of 16 genes across the clusters in HPRT3, HPRT2, and HPRT1 cells are also presented in Fig. S4E–G. Cells from each PDX were also clustered and displayed using Uniform Manifold Approximation and Projection (UMAP), with clusters shown on Fig. S4H–J and cluster features provided in Table S9. Features of clusters included BRD4-regulated genes such as MYC (HPRT1, HPRT3), MCL1 (HPRT1, HPRT3), BIRC3 (HPRT1, HPRT3), IRF4 (HPRT2, HPRT3), and TCF4 (HPRT2, HPRT3), as shown on UMAP feature plots in Figures S4K–M.

### Disparate sensitivity of RT-DLBCL cells to ibrutinib or BH3-mimetics correlates with protein expressions regulating their activity

**Fig. 3:**
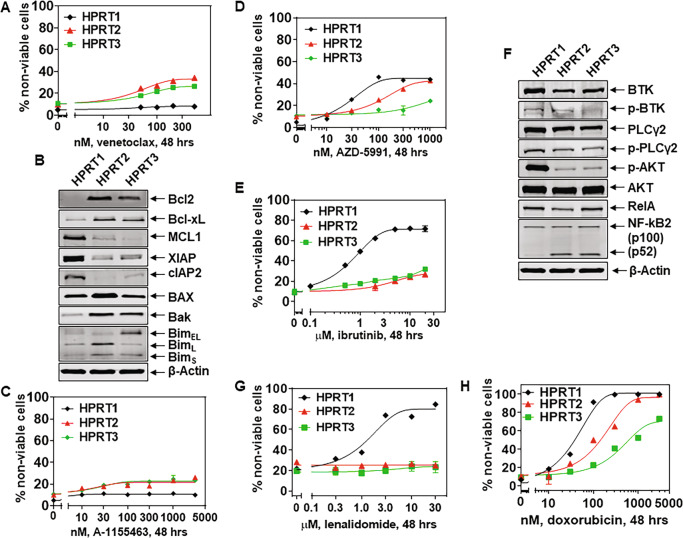
RT-DLBCL cells exhibit a diverse range of sensitivity to venetoclax, ibrutinib, lenalidomide, and doxorubicin. **A** RT-DLBCL cells were treated with the indicated concentrations of BCL2 inhibitor venetoclax, for 48 h. At the end of treatment, cells were washed with 1× PBS and stained with To-Pro-3 iodide. The % of non-viable cells was determined by flow cytometry. Mean of three experiments ± SEM. **B** Expression levels of BCL2 family proteins in HPRT1, HPRT2, and HPRT3 cells. The expression levels of β-Actin in the lysates served as the loading control. **C**–**E** RT-DLBCL cells were treated with the indicated concentrations of Bcl-xL-specific inhibitor A-1155463, MCL1 inhibitor AZD-5991, or ibrutinib, for 48 h. At the end of treatment, cells were washed with 1× PBS and stained with To-Pro-3 iodide. The % of non-viable cells was determined by flow cytometry. Mean of three experiments ± SEM. **F** Expression levels of B-cell receptor pathway proteins in HPRT1, HPRT2, and HPRT3 cells. The expression levels of β-Actin in the lysates served as the loading control. **G**–**H** RT-DLBCL cells were treated with the indicated concentrations of lenalidomide or doxorubicin for 48 h. At the end of treatment, cells were washed with 1× PBS and stained with To-Pro-3 iodide. The % of non-viable cells was determined by flow cytometry. Mean of three experiments ± SEM.

**Fig. 4:**
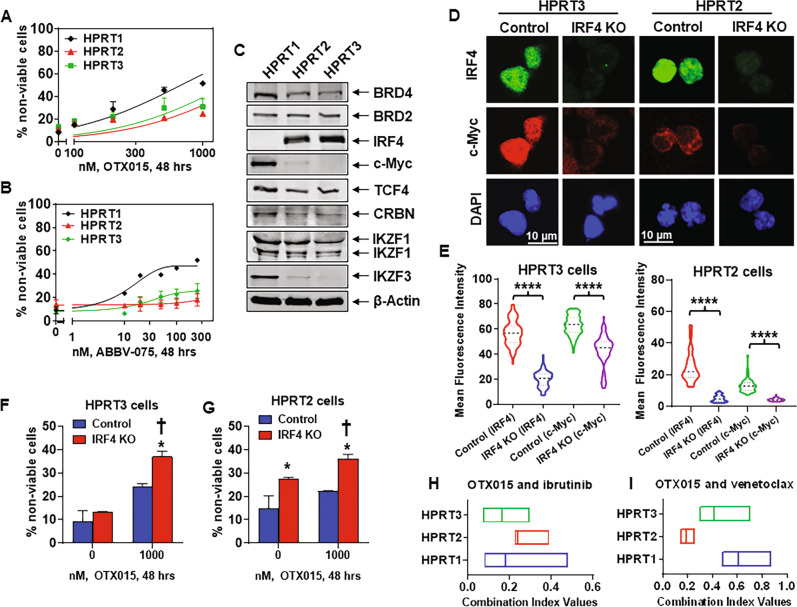
Knockout of IRF4 depletes c-Myc and increases sensitivity of HPRT3 and HPRT2 cells to OTX015, and co-treatment with BET inhibitor and ibrutinib or venetoclax exerts synergistic lethal activity in RT-DLBCL cells. **A**, **B** RT-DLBCL cells were treated with the indicated concentrations of OTX015 or ABBV-075 for 48 h. At the end of treatment, cells were washed with 1× PBS and stained with To-Pro-3 iodide. The % of non-viable cells was determined by flow cytometry. Mean of three experiments ± SEM. **C** Expression levels of BET proteins, IRF4, c-Myc and TCF4 in HPRT1, HPRT2, and HPRT3 cells. The expression levels of β-Actin in the lysates served as the loading control. **D** HPRT3 and HPRT2 cells were transfected with RNP complexes (sgNeg (Control) or 2 IRF4 sgRNAs (IRF4 KO) + recombinant Cas9) using an Amaxa Nucleofector II device. RT-DLBCL cells were cultured on a monolayer of HS5 cells for 5 days. At the end of treatment, cells were cytospun onto glass slides, fixed, permeabilized, and incubated with anti-IRF4 or anti-c-Myc antibody. Cells were washed with 1× PBS, then Alexa 488 or Alexa 594-conjugated secondary antibodies were added. Nuclei were stained with DAPI. Slides were treated with anti-fade reagent and mounting media to affix coverslips. Images were obtained by confocal microscopy. Original magnification is ×60. Scale bar indicates 10 microns. **E** Mean fluorescence intensity of IRF4 and c-Myc expression in HPRT3 and HPRT2 cells from two independent experiments transfected as in (**D**) and imaged by confocal microscopy. **F**–**G** HPRT3 and HPRT2 cells were transfected as in (**D**), and incubated on HS5 cells for 72 h. Following this, cells were plated and treated with OTX015 for 48 h. At the end of treatment, cells were washed with 1× PBS and stained with To-Pro-3 iodide. The % of To-Pro-3 iodide-positive, non-viable cells was determined by flow cytometry. *Indicates values that are significantly greater in HPRT3 or HPRT2 cells transfected with IRF4 sgRNAs and treated with OTX015 than cells transfected with sgNeg (Control) and treated with OTX015 determined by two-tailed, unpaired *t*-test. ^†^Indicates values significantly greater in OTX015-treated cells transfected with IRF4 sgRNAs (IRF4 KO) than untreated cells determined by two-tailed, unpaired *t*-test. **H**, **I** RT-DLBCL cells were treated with OTX015 (dose range: 250–1000 nM) and ibrutinib (dose range: 2–10 µM) or venetoclax (dose range: 20–500 nM) for 48 h. The % non-viable cells was determined by flow cytometry. Combination index (CI) values were calculated with CompuSyn and boxplots were generated with GraphPad V8. CI values <1.0 indicate a synergistic interaction of the two agents.

Previous reports had highlighted differential activity and response of RT-DLBCL cells to novel targeted agents such as BCL2 inhibitor venetoclax, and BTK inhibitor ibrutinib [11, 12, 14]. As shown in Fig. 3A, unlike the high level of sensitivity of CLL cells documented for venetoclax [35], the three RT-DLBCL cells were relatively less sensitive to venetoclax. Nonetheless, HPRT3 and HPRT2 were relatively more susceptible to venetoclax than HPRT1 cells. This correlated with higher BCL2, BAX, BAK, and BIM levels in HPRT3 and HPRT2 compared to HPRT1 cells (Fig. 3B). Higher Bcl-xL expression also correlated with increased sensitivity of HPRT3 and HPRT2 versus HPRT1 cells to a Bcl-xL-specific inhibitor A-1155463 (Fig. 3C) [36]. Conversely, higher MCL1 levels correlated with greater sensitivity of HPRT1 cells, compared to HPRT3 and HPRT2 cells, to an MCL1-specific inhibitor AZD-5991 (Fig. 3D) [36]. Notably, HPRT3 and HPRT2 were resistant to ibrutinib, as compared to HPRT1 cells (Fig. 3E). As was shown in a previous report, this is likely due to activation of the alternative MAP3K14-NFkB pathway, instead of the classical BCR-BTK-NFkB activation pathway, in HPRT3 and HPRT2 cells [37]. The alternative pathway involves increased processing of p100 to p52 by MAP3K14 (NIK kinase) resulting in activation of NFkB2 [37], which was noted in HPRT3 and HPRT2 cells (Fig. 3F). Despite expressing elevated levels of p-AKT, HPRT1 cells exhibited increased sensitivity to ibrutinib, which was consistent with increased p-BTK and p-PLCγ2 levels, suggesting increased BCR activity [38–40]. Compared to HPRT3 and HPRT2, HPRT1 cells also exhibited higher sensitivity to lenalidomide (Fig. 3G) [41]. This was associated with increased levels of cereblon and IKZF1/3 in HPRT1 cells compared to HPRT3 and HPRT2 cells (Fig. 4C, vide infra) [41, 42]. In contrast, the three RT-DLBCL cells displayed disparate level of sensitivity to the topoisomerase II inhibitor doxorubicin (Fig. 3H). Collectively, these findings demonstrated that the three RT-DLBCLs are differentially sensitive to several targeted agents, which correlated with expression levels of genes that regulate sensitivity to the targeted agents.

### Sensitivity of RT-DLBCL cells to BET inhibitor or its combination with ibrutinib or venetoclax

**Fig. 5:**
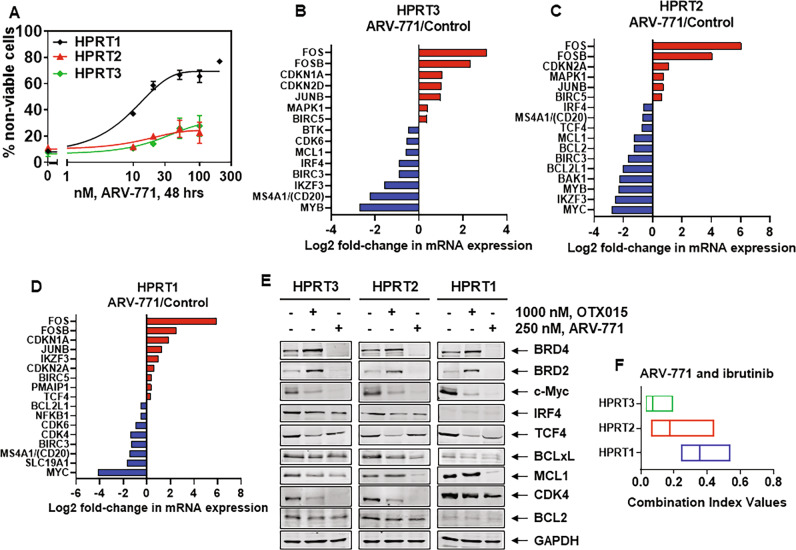
Treatment with BET-PROTAC ARV-771 depletes c-Myc, CDK4, TCF4, IRF4, and Bcl-xL expression and exerts synergistic lethal activity with ibrutinib in RT-DLBCL cells. **A** RT-DLBCL cells were treated with the indicated concentrations of ARV-771 for 48 h. At the end of treatment, the % of To-Pro-3 iodide-positive, non-viable cells was determined by flow cytometry. Mean of three experiments ± SEM. **B**–**D** Comparison of selected, significantly altered (≥1.25 fold change up or down and *p*-value < 0.05 relative to untreated control), mRNA expression changes detected by RNA-Seq analysis (on biologic triplicates) in HPRT3, HPRT2, and HPRT1 cells following treatment with BET-PROTAC ARV-771 for 8 h. **E** HPRT3, HPRT2, and HPRT1 cells were treated with 1000 nM of OTX015 or 250 nM of ARV-771 for 24 h. Immunoblot analyses were conducted on the cell lysates. The expression levels GAPDH in the cell lysates served as the loading control. **F** RT-DLBCL cells were treated with ARV-771 (dose range: 20–250 nM) and/or ibrutinib (dose range: 2–10 µM) for 48 h. The % of non-viable cells was determined by flow cytometry. Combination index (CI) values were calculated with CompuSyn and boxplots were graphed with GraphPad V8. CI values <1.0 indicate a synergistic interaction of the two agents.

We previously reported that BET inhibitors (BETis) either alone or in combinations with ibrutinib or venetoclax induce apoptosis in NHL including DLBCLs [27, 43]. Based on this, we determined the lethal activity of BETi OTX015 or ABBV-075 against the three RT-DLBCL cells. Treatment with OTX015 and ABBV-075 induced more lethality in HPRT1, compared to HPRT3 and HPRT2 cells (Fig. 4A, B). This correlated with higher levels of BRD4, c-Myc, and TCF4, but markedly lower levels of IRF4 in HPRT1 compared to HPRT3 and HPRT2 cells (Fig. 4C). Levels of DUB3, SPOP and TRIM33/24, previously reported to be involved in regulating sensitivity to BETis [44–46], were neither significantly different nor altered by OTX015 treatment in the three RT-DLBCL cells (Fig. S5A). RNA-Seq analysis showed that following treatment with OTX015 larger numbers of mRNA levels were up or downregulated in HPRT1 compared to HPRT3 and HPRT2 cells (Fig. S5B, C). The Venn diagram in Fig. S5C shows that 540 genes were commonly depleted whereas 574 genes were commonly induced by OTX015 treatment of the three RT-DLBCL cells. Among the mRNA expressions demonstrating greater than log2-fold inhibition (e.g., −0.3219) in HPRT1 cells were those of SLC19A1, MYC, MYB, BIRC3, BTK, CDK4/6, NFkB1, IRF4, and BCL2L1, while CDKN1A, PMAIP1, and HEXIM1 mRNA levels showed log2-fold increases (Fig. S5D). QPCR analysis showed that OTX015 treatment inhibited mRNA levels of the super-enhancer-driven MYC and CDK6 oncogenes, while inducing HEXIM1 mRNA levels in all three RT-DLBCL cell-types (Fig. S5E and Table S10) [20, 47]. We next determined whether high levels of IRF4 in HPRT3 and HPRT2 cells confer resistance to BETi treatment. Via CRISPR-Cas9, IRF4 was knocked out in HPRT3 and HPRT2 cells. Figure 4D, E demonstrate that, compared to the control, nuclear levels of IRF4 were markedly depleted in HPRT3 and HPRT2 cells in which IRF4 had been knocked out (labeled IRF4 KO). Notably, depletion of nuclear IRF4 in IRF4 KO HPRT3 and HPRT2 cells was accompanied by repression of nuclear levels of c-Myc, which is a transcriptional target of IRF4 in B cells (Fig. 4D, E) [48]. Importantly, as compared to the control, depletion of IRF4 and c-Myc significantly increased sensitivity to OTX015 in IRF4 KO cells, suggesting that IRF4 overexpression attenuates BETi-induced lethality in RT-DLBCL cells (Fig. 4F, G). We also determined the effect of ectopic overexpression of c-Myc, (driven by an EF1α promoter) via nucleofection into HPRT3 and HPRT2 cells. Although c-Myc overexpression increased % non-viable cells, it significantly increased OTX015-induced lethality in HPRT3 and HPRT2 cells (Fig. S6A, B). Additionally, we determined the effect of CDK9 inhibitor NVP-2 and AZD4573 on the viability of the RT-DLBCL cells. As shown in Fig. S7A, B, treatment with NVP-2 and AZD4573 dose-dependently induced loss of viability of the RT-DLBCL cells. Treatment with NVP-2 also reduced the levels of serine 2 (S2) phosphorylated RNAP2, IRF4, c-Myc, MCL1 and Bcl-xL, while increasing the levels of cleaved PARP in HPRT3, HPRT2, and HPRT1 cells (Fig. S7C, D). Next, despite observing disparate sensitivity to each of the agents alone, we determined whether co-treatment with BETi and ibrutinib or venetoclax would exert synergistic lethality against the three RT-DLBCL cell-types. Figures 4H and I and Table S11 demonstrate that co-treatment with OTX015 and ibrutinib or venetoclax is synergistically lethal in all three RT-DLBCL cell-types [27, 36, 49]. The synergistic activity of OTX015 with venetoclax may be partially explained by OTX015-mediated decline in the levels of MCL1 (vide infra Fig. 5E).

### Preclinical efficacy of BET-PROTAC alone and in combination with ibrutinib or venetoclax against RT-DLBCL cells

**Fig. 6:**
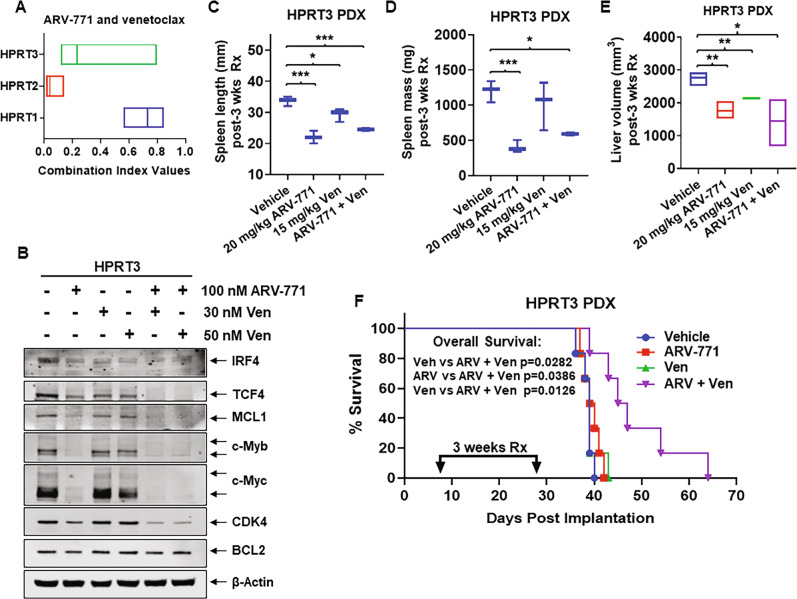
Co-treatment with ARV-771 and venetoclax (ABT-199) reduces lymphoma burden in the spleen and liver as well as significantly improves survival of NSG mice engrafted with clonally-related HPRT3-DLBCL cells. **A** RT-DLBCL cells were treated with ARV-771 (dose range: 20–250 nM) and venetoclax (dose range: 20–100 nM) for 48 h. The % of non-viable cells was determined by flow cytometry. Combination index (CI) values were calculated with CompuSyn and boxplots were generated with GraphPad V8. CI values <1.0 indicate a synergistic interaction of the two agents. **B** HPRT3 cells were treated with ARV-771 and venetoclax (Ven) as indicated for 24 h. At the end of treatment, total cell lysates were prepared and immunoblot analyses were conducted. The expression levels of β-Actin in the cell lysates served as the loading control. **C**, **D**. HPRT3 cells were engrafted into NSG mice by lateral tail-vein injection. Following documentation of engraftment, mice were treated with ARV-771 and venetoclax (Ven), as indicated for 3 weeks. Mice were sacrificed and spleen length and spleen mass were determined. *p < 0.05; ***p < 0.005 as determined by two-tailed, unpaired *t*-test. **E** HPRT3 cells were engrafted into NSG mice by lateral tail-vein injection. Following documentation of engraftment, mice were treated with ARV-771 and venetoclax (Ven) as indicated for 3 weeks. Mice were imaged by MRI to document effects of treatment on liver volume. *p < 0.05; **p < 0.01 as determined by two-tailed, unpaired *t*-test. **F** Kaplan–Meier survival plot of NSG mice treated with 20 mg/kg of ARV-771 and/or 15 mg/kg venetoclax for 3 weeks. Significant differences in overall survival between the vehicle and treated mice were determined by Mantel–Cox log rank test.

We had previously shown that BET-PROTACs degrade BET proteins and cause more profound perturbations in mRNA and protein levels, especially of c-Myc and other super-enhancer driven oncogenes, as well induce more apoptosis than BETi in NHL cells [43]. Therefore, we next evaluated effects of the BET-PROTAC ARV-771 on mRNA and protein expressions of RT-DLBCL-relevant oncogenes, as well as on survival of the RT-DLBCL cell-types. Figure 5A shows that ARV-771 induced greater lethality in HPRT1 (~65% loss of viability due to exposure to 50–100 nM of ARV-771), compared to HPRT3 and HPRT2 cells, which exhibited ~20% loss of viability following exposure to 50–100 nM of ARV-771. Concomitantly, RNA-Seq analyses demonstrated that ARV-771 induced more mRNA perturbations (up or downregulations) in HPRT1 compared to HPRT3 and HPRT2 cells (Fig. S8A–C). There were also more gene expression perturbations that overlapped between OTX015 and ARV-771 treatments in HPRT1 compared to HPRT3 and HPRT2 cells (Figs. S8D–F). RNA-Seq data showed log2-fold decline in the expression levels of MYB, IKZF3, IRF4, BIRC3, MCL1, CDK6, and BTK, with increases in CDKN1A and CDKN2D in HPRT3 and HPRT2 cells (Fig. 5B, C). Due to MYC amplification and increased expression of c-Myc, ARV-771 treatment caused more pronounced repression of MYC and its target SLC19A1 (folate transporter) in HPRT1 cells (Fig. 5D) [50]. As previously reported in other cell types, in the three RT-DLBCL cells also, treatment with BETi OTX015 induced BRD4 levels (Fig. 5E) [43]. In contrast, ARV-771 treatment depleted BRD4 and BRD2 levels in the three RT-DLBCL cell-types (Fig. 5E). Although ARV-771 exerts similar effects on protein expressions of the genes shown in Fig. 5E, HPRT1 cells express higher levels of c-Myc, TCF4, MCL1, and CDK4 on which they may be more dependent for growth and survival. Therefore, more pronounced decline in the levels of these proteins due to ARV-771 treatment may exert greater lethality in HPRT1 compared to HPRT3 and HPRT2 cells (Fig. 5E). Similar to BETi-based combinations, we also determined lethal effects of co-treatment with ARV-771 and ibrutinib or venetoclax in the three RT-DLBCL cell types. Co-treatment with ARV-771 and ibrutinib or venetoclax exerted synergistic lethality in all three RT-DLBCL cell-types (Figs. 5F and 6A). This synergy with venetoclax may be partially explained by ARV-771-mediated decline in the levels of MCL1. Finally, we determined the in vivo efficacy of BET-PROTAC ARV-771 and/or venetoclax against the PDX model of HPRT3 cells administered via tail-vein infusion and engrafted in NSG mice. Ex vivo exposure of HPRT3 cells to ARV-771 and venetoclax mediated more decline in IRF4, TCF4, c-Myc, c-Myb, MCL1, and CDK4 protein levels, as compared to treatment with ARV-771 alone (Fig. 6B). Following engraftment and 7-days post-implantation, compared to vehicle control, daily treatment for three weeks with ARV-771 and venetoclax significantly reduced the spleen length and mass, as well as reduced the liver volume, highlighting reduction in tumor volume in the mice engrafted with HPRT3 cells (Fig. 6C–E). Notably, mice co-treated with ARV-771 and venetoclax demonstrated significantly improved overall survival, as compared to mice treated with vehicle control, or with venetoclax or ARV-771 alone (*p* < 0.05) (Fig. 6F).

## Discussion

To address the preceding absence and unmet need for pre-clinical RT-DLBCL models, we report here generation and characterization of the first-ever, PDX models of CLR and CLUR RT-DLBCLs. Utilizing traditional cytogenetics, array-CGH, NGS, ATAC-Seq, ChIP-Seq, scRNA-Seq, and Western analyses, a full characterization of genomics/epigenomics and gene-expression profiles of the RT-DLBCL models is also presented. The large array of genetic alterations in HPRT3 and HPRT2 cells, representative of CLR or CLUR RT-DLBCLs, is a reflection of the rich profile of recurrent mutations previously described in the coding and non-coding genes during CLL progression [51, 52]. The high numbers of genetic alterations in the CLR and CLUR RT-DLBCLs complicated and limited the identification of genomic targets for therapy. Therefore, it was necessary to correlate the gene expressions in the RT-DLBCL cells with sensitivity to standard and novel therapeutic agents. These correlations demonstrated that, although individually the drugs are only modestly active, combinations of BETi or BET-PROTAC with ibrutinib or venetoclax were synergistically lethal against the CLR and CLUR RT-DLBCL cells. Notably, co-treatment with BET-PROTAC and venetoclax also reduced lymphoma burden and improved survival of NSG mice engrafted with CLR RT-DLBCL cells.

Based on the presence of IGH gene rearrangement, HPRT3 cells were CLR, HPRT2 CLUR, and HPRT1 were of indeterminate clonal origin, compared to their respective preceding CLL clones. Phenotypically, all three cells expressed CD20, but only HPRT3 and HPRT2 cells expressed high levels of IRF4, suggesting their post-germinal cell of origin [2, 5]. These observations were consistent with immunohistochemistry-determined protein expressions documented in the large cohort of 52 RT-DLBCLs. They showed uniformly positive expression of PAX5 and IRF4 (MUM1), whereas CD10 expression was absent. Phenotypic protein expression patterns indicated that HPRT3 and HPRT2 cells were of ABC sub-type of DLBCL, as were the vast majority of RT-DLBCLs [2, 5]. Although HPRT2 and HPRT1 cells were low-expressers of CD5 and CD23, HPRT3 cells expressed higher surface CD5 and CD23, as was also seen in some samples among the larger cohort of RT-DLBCLs. HPRT1 cells expressed high levels of CD10 and BCL6, which was consistent with their GCB cell phenotype [2]. These cells were also low expressers of PD-1, which is rarely expressed by de novo DLBCL [2, 5]. Additionally, compared to HPRT3 and HPRT2 cells, HPRT1 cells expressed higher levels of c-Myc and Ki67, which is consistent with high cell cycle S-phase status, and is explained by the presence of MYC amplification in HPRT1 cells. Consistent with other reports, the RT-DLBCLs presented here were also negative for EB virus infection [2, 5]. Also, similar to prior reports, cytogenetics analysis showed profound aneuploidy with presence of marked chromosomal tetrasomy and trisomy in CLR HPRT3 cells [8], compared to CLUR HPRT2 and HPRT1 cells. This was corroborated via array-CGH and low-pass WGS, which demonstrated large numbers of chromosomal amplifications and losses as well as arrays of copy gains and losses, respectively, in the CLR HPRT3 compared to either CLUR HPRT2 or the de novo-GCB-DLBCL HPRT1 cells. Whole exome NGS analysis of 300 genes also underscored the high numbers of genetic mutations in HPRT3 and HPRT2 cells compared to the mutationally relatively more quiescent HPRT1 cells. Overall, observed mutations in the RT-DLBCL cells involved genes of epigenetic modifiers, transcription factors, surface receptors, signaling kinases, DNA damage/response pathway, and apoptosis threshold regulators. Although genetic alterations in NOTCH, MYC, and DNA damage/repair pathways suggested likely sensitivity of HPRT1 cells to agents that target these dependencies, presence of multiple genetic alterations in HPRT3 and HPRT2 cells defied clear delineation of dependencies just based on their profile of genetic mutations. However, epigenetic and gene-expression analyses illuminated potentially more targetable dependencies in HPRT3 and HPRT2 cells.

All three RT-DLBCL cells displayed more open chromatin and increased H3K27Ac occupancy at the SEs/Es, as compared to normal CD34+ progenitor cells. More granularly, increased chromatin accessibility at the MYC, TCF4, and PLCγ2 loci was also accompanied by higher H3K27Ac occupancy at the Es of these genes. Additionally, open chromatin at the IRF4 and BCL2 loci was associated with increased H3K27Ac occupancy at their Es, but only in HPRT3 and HPRT2, not in HPRT1 cells. Notably, high copy-gains of TCF4 in HPRT3 and HPRT2 cells, observed via low-pass WGS, was associated with increased chromatin accessibility and activity of TCF4 SE, highlighted by increased occupancy with H3K27Ac and BRD4, as recently reported [28]. This report had also shown that copy gains of TCF4 at 18q21.1 are commonly found in ABC subtype of de novo DLBCL, which explains why TCF4 copy gains were present in the ABC subtype of HPRT3 and HPRT2 RT-DLBCL cells [28]. BRD4 occupancy was also increased at the active enhancers of IRF4, PAX5, and MYC in HPRT3 and HPRT2 cells. Consistent with its highly aneuploid status and highest level of active chromatin, scRNA-Seq analyses showed that HPRT3 cells exhibited highest number of transcriptionally active cell-clusters, although high mRNA expressions of TCF4, PAX5, and IRF4 were observed in HPRT3 and HPRT2 cells. Even though IRF4 and TCF4 induce MYC, scRNA-Seq showed only a muted MYC mRNA expression in HPRT3 and HPRT2 cells [28, 48]. Conversely, uniformly high Myc expression in HPRT1 cells was explained by amplification of MYC and high occupancy of MYC chromatin by H3K27Ac and BRD4. Although not shown here, high TCF4 expression in HPRT1 cells may be due to high H3K27Ac and BRD4 occupancy at the SE of TCF4, and by MYC-facilitated transcription of TCF4 [28, 53]. In all the RT-DLBCL cells, their mRNA levels, determined by scRNA-Seq, also generally corresponded with the protein levels of BCL2 family of proteins, including BCL2, MCL1, Bcl-xL, BAX, BAK, BIM (BCL2L11), as well as with the protein expression of c-IAP2 (BIRC3), XIAP, and Ki67 (MKI67).

Overall, the mRNA and protein expression levels correlated with specific drug-sensitivities of the RT-DLBCL cells. Notably, high BCL2 and Bcl-xL, concomitantly with low expression of MCL1 in HPRT3 and HPRT2 versus HPRT1 cells, respectively correlated with higher sensitivity to venetoclax and A-1155463 (Bcl-xL inhibitor) and lesser sensitivity to AZD-5991 (MCL1 inhibitor)-induced cell lethality. Additionally, in HPRT3 and HPRT2 versus HPRT1 cells, high NFkB2/p52 levels conferred relative resistance to ibrutinib [37]. Although of GCB-DLBCL sub-type, HPRT1 cells exhibited more sensitivity to ibrutinib, likely due to higher activities and levels of p-BTK and p-PLCγ2 (Fig. 4F). Higher cereblon and IKZF1/3 levels in HPRT1 cells, compared to HPRT3 and HPRT2 cells (Fig. 5C), may also explain the greater sensitivity of HPRT1 cells to lenalidomide, which induces cereblon-mediated degradation of IKZF1/3 in transformed B cells [41, 42]. Conversely, considerably higher levels of sensitivity of HPRT1 compared to HPRT3 and HPRT2 cells to BETis OTX015 and ABBV-075, and to the BET-PROTAC ARV-771, correlated with higher protein expressions of BRD4, c-Myc, and TCF4 in HPRT1 cells. The latter gene-expressions are well known to be driven by super-enhancers with high BRD4 occupancy [19, 25, 28]. It is noteworthy that previously reported mechanisms of resistance to BETi, such as high levels/activity of DUB3, TRIM33, SPOP or p-AKT, were not observed and did not appear to contribute to the disparate sensitivity of HPRT3 and HPRT2 versus HPRT1 cells to BETi [44–46]. However, markedly higher expression levels of IRF4 did mechanistically contribute to partial resistance of HPRT3 and HPRT2 cells to BETi treatment, since IRF4 knockout via CRISPR-Cas9 partially re-sensitized the cells to BETi. IRF4 depletion resulted in reduced nuclear c-Myc expression, which would explain increased sensitivity of HPRT3 and HPRT2 cells to BETi-induced lethality. To determine whether the modest single-agent activity of BETi, or ARV-771 could be improved against the RT-DLBCL cells, especially against the poor-risk CLR-RT-DLBCL HPRT3 cells, we interrogated the lethal activity of BETi or ARV-771 combined with venetoclax or ibrutinib. Indeed, co-treatment with BETi or ARV-771 exerted synergistic in vitro lethality against all three DLBCLs. Consistent with higher in vitro activity of venetoclax against HPRT3 cells, co-treatment with relatively low doses of ARV-771 and venetoclax significantly reduced in vivo HPRT3 cell burden and improved survival of immune-depleted mice engrafted with HPRT3 cells, without resulting in appreciable host toxicity. These findings strongly support further in vivo testing and development of BETi or BET-PROTAC-based combinations with other BH3 mimetics, BCR-signaling kinase inhibitors, as well as with other novel targeted agents, utilizing the newly established and characterized models of RT-DLBCLs.

## Supplementary information

Supplemental Figures and Tables

Supplemental Figure Legends

Supplemental Methods and Materials

Table S9
