# Supplementary material for: BET proteolysis targeted chimera-based therapy of novel models of Richter Transformation-diffuse large B-cell lymphoma
Source: Leukemia. 2021 Mar 2;35(9):2621–34. doi: 10.1038/s41375-021-01181-w (PMC8410602; doi:10.1038/s41375-021-01181-w)
Supplement: Supplementary file 1 — Supplemental Figures and Tables [file 41375_2021_1181_MOESM1_ESM.pdf]

A

Cell cycle status of RT-DLBCL cells

	G0/G1	S	G2/M
HPRT3	76.1 ± 1.6	11.4 ± 0.6	11.5 ± 0.8
HPRT2	81.2 ± 1.3	6.7 ± 0.8	6.6 ± 2.5
HPRT1	46.6 ± 1.2	35.9 ± 0.4	17.5 ± 0.9

B

HPRT1 MYC FISH

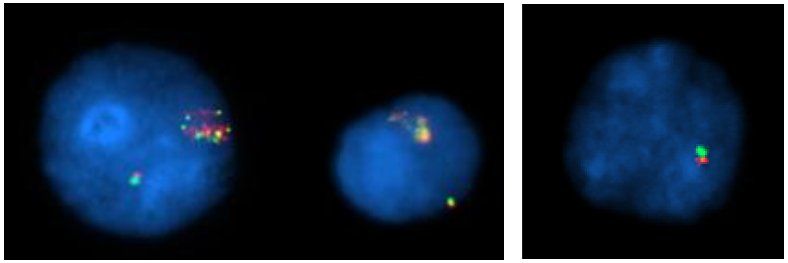

5’ MYC amplification   3’ MYC deletion

C

IRF4   CD10   BCL6

HPRT3

HPRT2

HPRT1

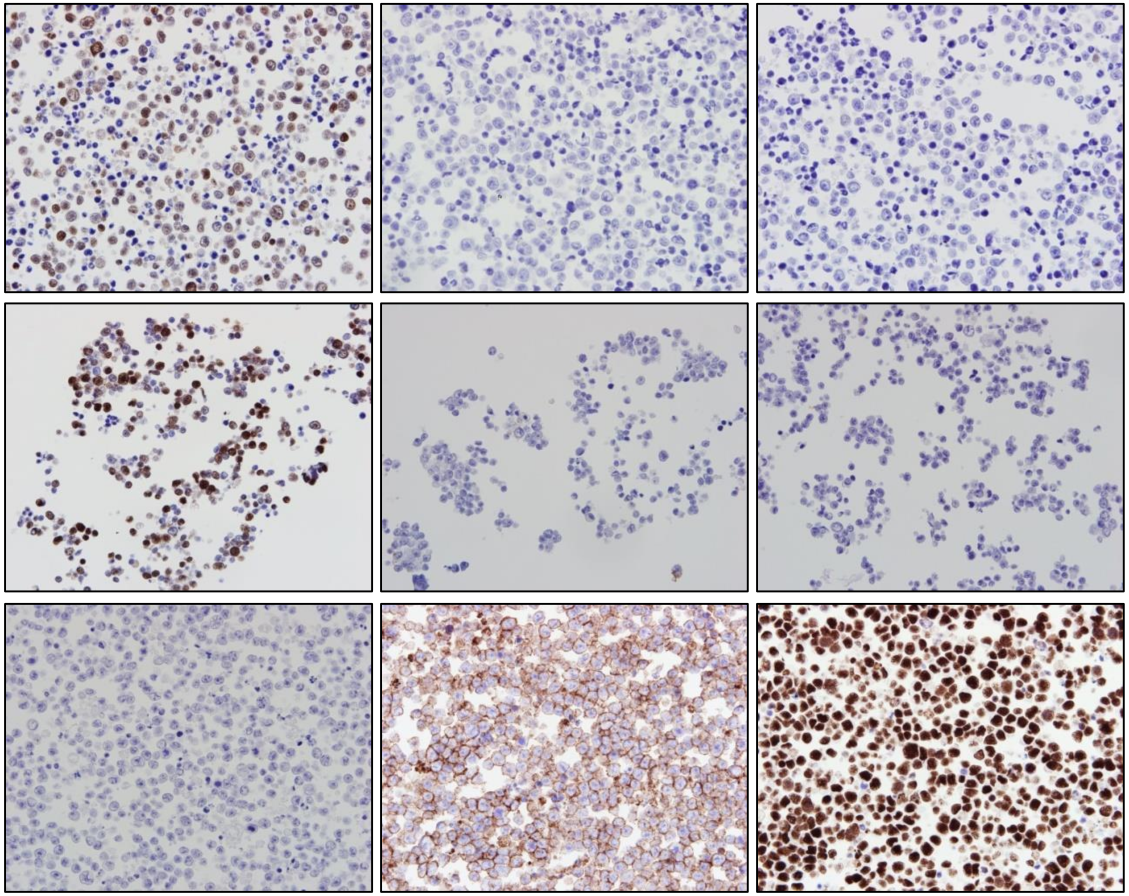

D

+ control   HPRT2   HPRT3   HPRT1

1 kb →

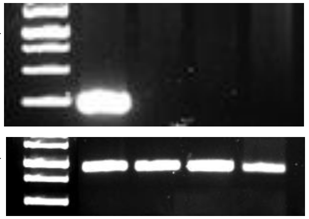

← EBV

1 kb →

← Internal control

E

+ control   HPRT2   HPRT3   HPRT1

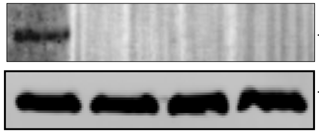

← EBNA2

← β-Actin

Figure S1

Path ID	Tissue type	PAX5	MUM-1	CD5	CD10	BCL-6	CD30
1	Soft tissue mass		1	0	0	1	
2	Bone marrow	1		D	0	0	
3	Lymph node	1		0	0	0	0
4	Lymph node	1	1	1	0	1	
5	Nasal mass	1	1	0	0	1	0
6	Lymph node		1	1			
7	Lymph node		1	0	0	1	
8	Breast mass	1	1	0	0	1	
9	Breast mass		1	1	0	0	
10	Lymph node			1	0		
11	Lymph node						0
12	Lymph node	1	1	D	0	1	0
13	Lymph node	1	1	1	0		0
14	Retroperitoneal mass	1		D			
15	Lymph node	1		1			
16	Lymph node						
17	Lymph node	1		1			0
18	Lymph node	1	1	1	0	0	0
19	Axilla mass			1			
20	Lymph node	1		D			
21	Lymph node	1		1			
22	Lymph node			1			
23	Lymph node	D		1			
24	Rib mass		1	0	1	1	0
25	Lymph node	1	1	1	0	1	0
26	Lymph node	1	1	1	0	0	0
27	Chest wall mass	1	1	0	0	0	
28	Lung mass	1	1	0	0	1	
29	Tonsil mass	1	1	1	0	1	0
30	Lymph node	1		D	0		0
31	Lymph node	1	1	1	0	1	
32	Lymph node	1		1	0		
33	Lymph node	1	1	1	0	0	
34	Lymph node	1		D			
35	Bone mass		1	D	0	1	
36	Lymph node		1	1			
37	Lymph node	1	0	1	0	1	
38	Neck mass	1	1		0	1	1
39	Lymph node		1	1	0	0	0
40	Lymph node			1			
41	Lymph node	1	1	1	0	1	
42	Lymph node	1	1	1	0	0	
43	Lymph node	1		1			
44	Lower extremity mass	1		0			
45	Lymph node	1	1	1		1	
46	Abdominal mass	1	1	1	0	1	
47	Lymph node		1	1	0	1	
48	Neck mass	1	1	1	0	1	
49	Lymph node	1		1	0		
50	Lymph node	1		D			
51	Lymph node	1		D			1
52	Lymph node	1	1	D	0	1	

Table S1

Table S2. Immuno-histochemical analysis of PAX5, IRF4, CD5, CD10, BCL6, and CD30 expression in tissues from 52 cases of Richter Transformation. Tabulated results of immuno-histochemical analysis of PAX5, IRF4, CD5, CD10, BCL6, and CD30 expression in tissues from 52 cases of Richter Transformation.

	Total tested	Positive	% positive
PAX5	38	38	100
MUM1 (IRF4)	30	29	96.7
CD5	50	41	82.0
CD10	33	1	3.0
BCL6	29	20	69.0
CD30	15	2	13.3

Table S2

Table S3. Clonal relationship of HPRT3, HPRT2, and HPRT1 RT-DLBCL patient-derived xenograft (PDX) models to antecedent CLL. Immunoglobulin heavy chain (IGH) status and somatic hypermutation (SH) was utilized to establish the clonal relationship of HPRT3, HPRT2, and HPRT1 DLBCL cells with their antecedent CLL.
FR= Framework regions of the VH segment of the IGH gene.

Sample	IG Heavy Chain	SH	Clonal Relationship
Antecedent CLL of HPRT3	FR1:357, FR2:300, FR3:136	V1-69_J6, 0% difference	Identical clone by IGH. SH not contributory
HPRT3	FR1:357, FR2:300, FR3:Failed	SH failed.	
Antecedent CLL of HPRT2	FR1: 316, 351; FR2: 285; FR3: 87, 127	2 bp (0.7%) difference, V4-61; V4-61_J4, 0.67% difference	Different clone by both IGH and SH
HPRT2	FR1: 323, FR3: 97	V3-74_J5, 8.11% difference	
Antecedent CLL of HPRT1	Failed analysis. No result.	FFPE sample. SH not possible	Indeterminate
HPRT1	FR1:316, FR2:254, FR3:95	SH failed.	

A

Karyotype analysis

HPRT3	HPRT2	HPRT1
86~88,XXX,-X,del(3)(p13p25)x2, add(4)(q35)x2,-5,-6,del(6)(q13q23)x2,-8,-8, -8, add(9)(p22)x2,-10,del(11)(q22q23)x2, -12,-13, del(13)(q12q22)x2,-14,-15,-16, add(16)(q24)x2,-17, add(17)(p11.2)x2, -19, add(19)(p13.3)x2,-20,-21, +5~11mar[cp9]/46,XX[11]	48-50,XY,del(X)(q24),-2, +3,del(3)(q21), add(7)(p22),add(8)(p21),+12, add(14)(q32), -15, -16,-17,+18,-19,+21, add(21)(q22),+3~5mar [cp20]	45~46,add(X)(p22.1),add(X)(q28),add(1)(p12),add(2)(q37),del(2)(q32q36),-3, del(6)(p21.1p24),add(7)(q36),der(8)hsr (8)(q24.2),add(11)(q13),der(11)dup(11)(q13q25),add(12)(p13),add(18)(q23),+mar[cp19]/ 54,idem,+1,+5,+13,+14,+17,+19,+20,+20,-22,+mar[1]

HPRT3

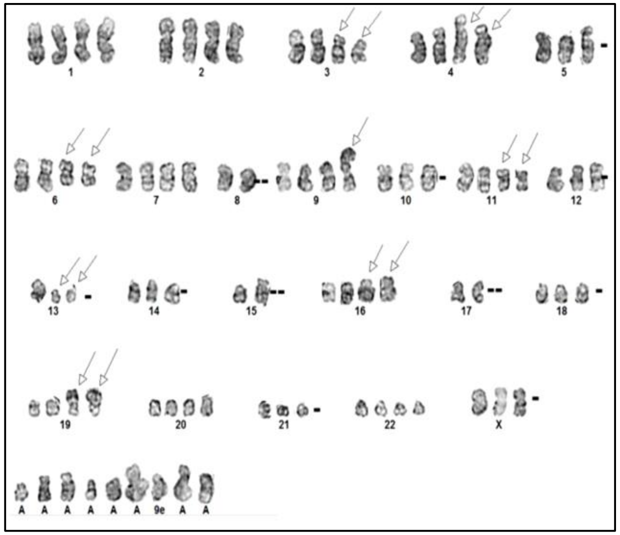

HPRT2

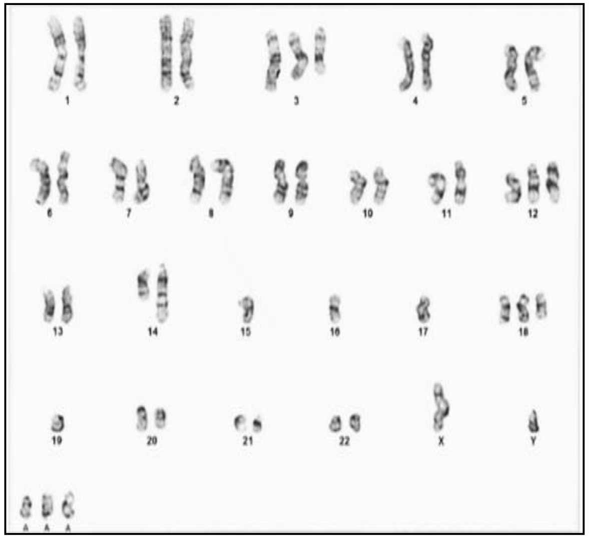

HPRT1

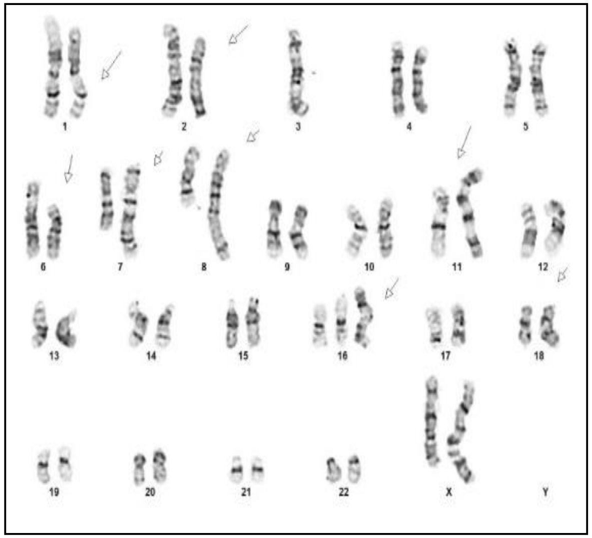

Figure S2

B

HPRT3

Chromosome	Gain/Loss	Cytobands
1	LOSS	p36.33 - p35.1
2	GAIN	p25.3 - p14
2	GAIN	q24.1 - q32.3
3	LOSS	p26.3 - p21.1
3	GAIN	p21.2 - q29
4	LOSS	q32.3 - q35.2
4	GAIN	p16.3 - p11
4	GAIN	q21.1
4	GAIN	q27 - q32.2
5	GAIN	p15.33 - q35.3
6	GAIN	q12 - q23.2
6	LOSS	p25.3 - p11.2
7	GAIN	p22.3 - q36.3
8	GAIN	p23.3 - p22
8	LOSS	p22 - q12.3
8	GAIN	q12.3 - q24.3
9	GAIN	q13 - q34.3
9	LOSS	p24.3 - p21.2
9	GAIN	p21.2 - p11.2
10	GAIN	q21.1 - q21.3
10	GAIN	q23.2 - q24.2
10	GAIN	q22.3 - q23.1
11	GAIN	p15.5 - q21
11	LOSS	q21 - q24.2
11	GAIN	q24.2 - q25
12	GAIN	p13.33 - p13.1
12	GAIN	q12 - q13.13
12	GAIN	q21.32 - q23.3
13	GAIN	q12.11 - q34
14	GAIN	q11.2
15	GAIN	q11.1 - q21.1
16	GAIN	p13.3 - q24.3
17	LOSS	p13.3 - p11.2
17	GAIN	p11.2
18	GAIN	p11.32 - q23
19	LOSS	p13.3
19	GAIN	p13.3 - q13.11
20	GAIN	p13 - p11.1
22	GAIN	q11.1 - q13.33

HPRT2

Chromosome	Gain/Loss	Cytobands
1	Loss	1p35.3 - p35.1
3	Gain	3p26.3 - q13.12
6	Loss	6q23.3 - p25.1
8	Loss	8p23.3 - p22
8	Gain	8p21.12 - q24.3
9	Gain	9p21.11 - q34.3
12	Gain	Trisomy 12
15	Loss	15q15.1 - q21.1
16	Loss	16q11.2 - q24.3
17	Loss	17p13.1
17	Loss	17q11.2
18	Gain	Trisomy 18

HPRT1

Chromosome	Gain/Loss	Cytobands
1	Loss	p32.3 - p31.1
2	Gain	q24.1 - q24.3
2	Loss	q33.2 - q37.3
3	Loss	p21.31
3	Loss	q13.13- q13.31
3	Gain	p21.31 - p21.2
3	Gain	q13.11- q13.13
6	Loss	p25.3 - p21.2
7	Gain	q31.1 - q36.3
7	Gain	q21.11 - q22.1
8	Gain	q23.3 - q24.21
8	Loss	p23.3 - p22
11	Gain	q12.1 - q12.2
11	Gain	q13.1 - q23.3
11	Loss	q23.3 - q25
18	Loss	q21.31 - q23
19	Gain	p13.3 - p13.2

Figure S2

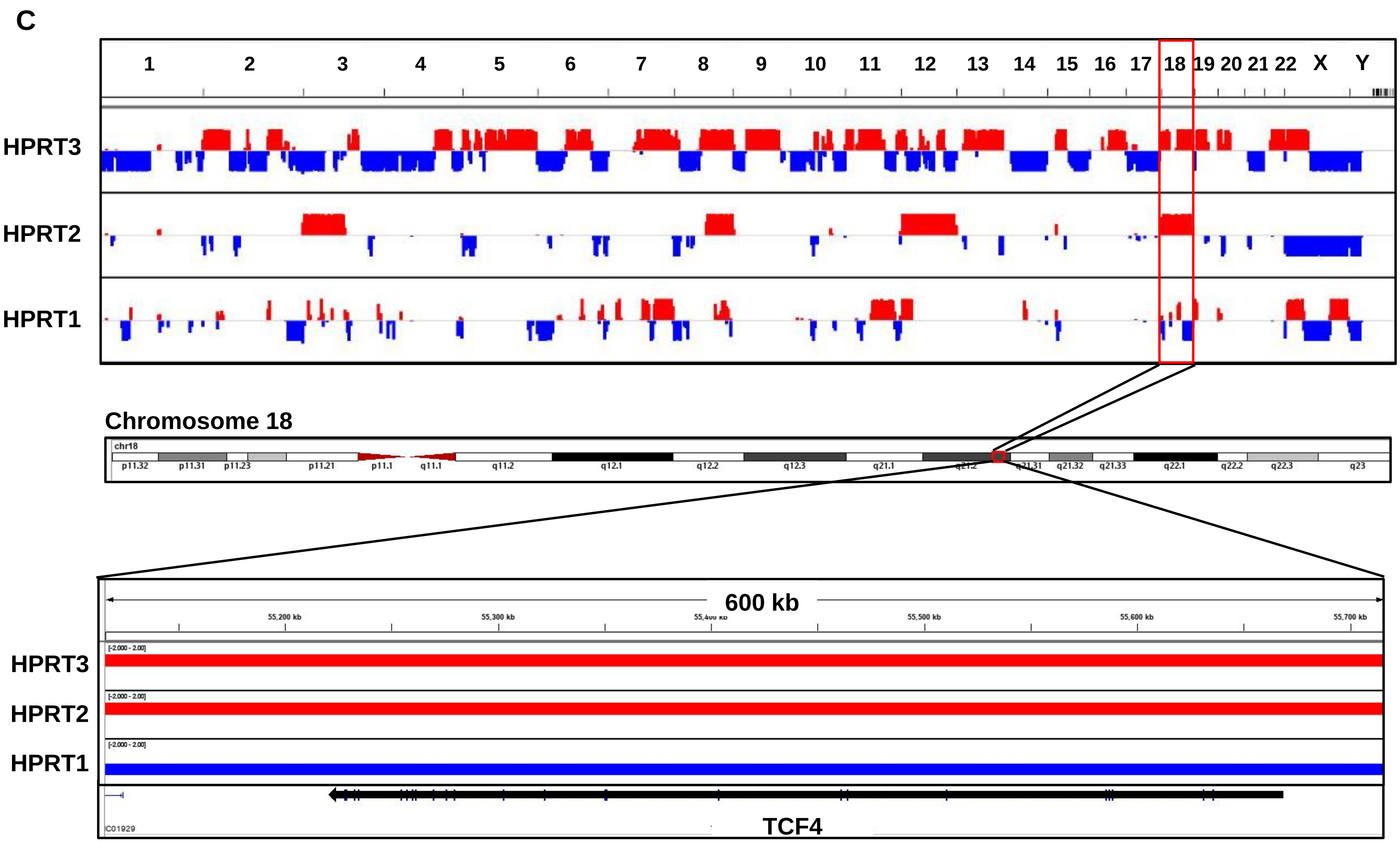
C
1
2
3
4
5
6
7
8
9
10
11
12
13
14
15
16
17
18
19
20
21
22
X
Y
HPRT3
HPRT2
HPRT1
Chromosome 18
chr18
p11.32
p11.31
p11.23
p11.21
p11.1
q11.1
q11.2
q12.1
q12.2
q12.3
q21.1
q21.2
q21.31
q21.32
q21.33
q22.1
q22.2
q22.3
q23
600 kb
55,200 kb
55,300 kb
55,500 kb
55,600 kb
55,700 kb
HPRT3
HPRT2
HPRT1
TCF4

Figure S2

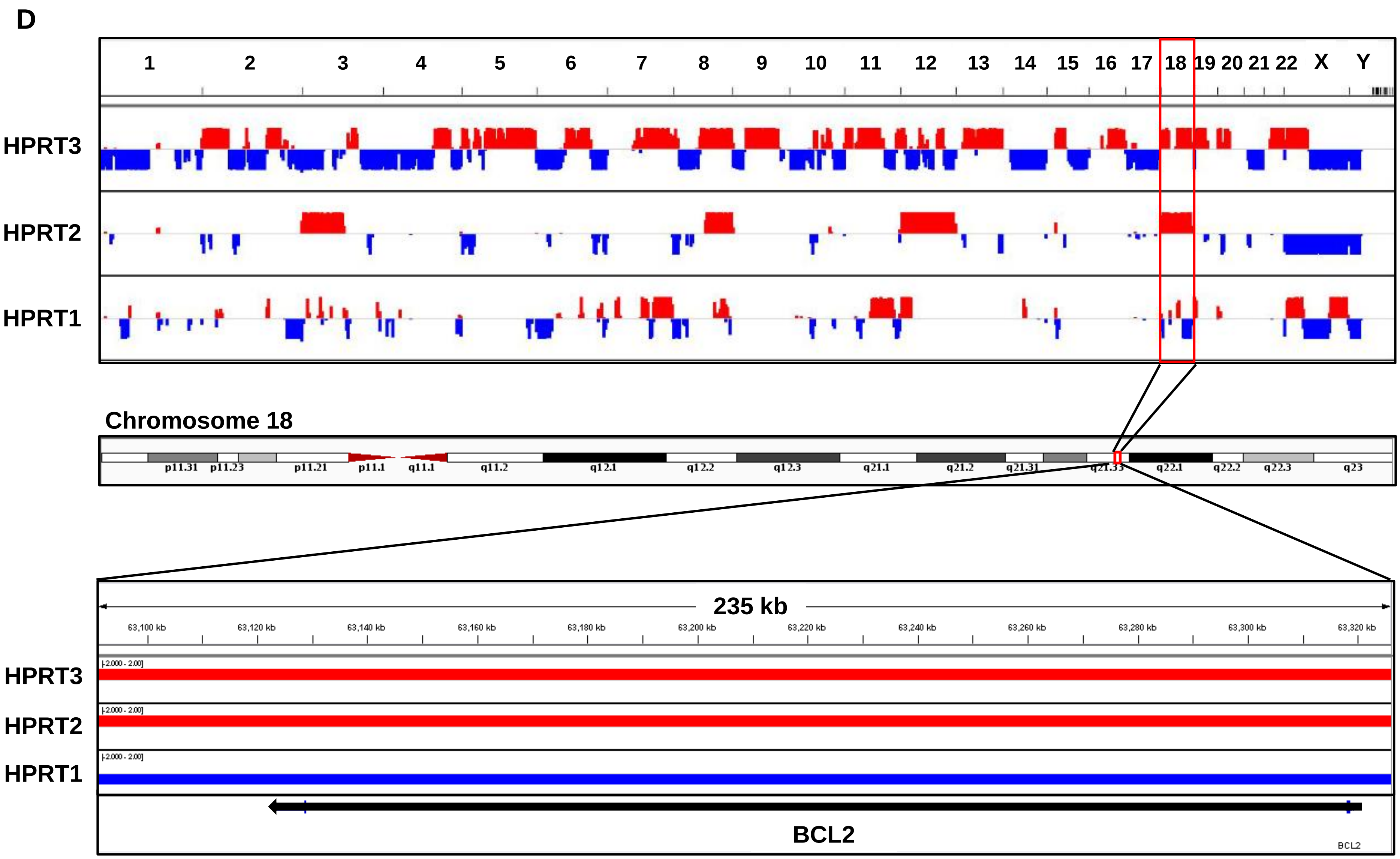
D
1
2
3
4
5
6
7
8
9
10
11
12
13
14
15
16
17
18
19
20
21
22
X
Y
HPRT3
HPRT2
HPRT1
Chromosome 18
p11.31
p11.23
p11.21
p11.1
q11.1
q11.2
q12.1
q12.2
q12.3
q21.1
q21.2
q21.31
q21.33
q22.1
q22.2
q22.3
q23
235 kb
63,100 kb
63,120 kb
63,140 kb
63,160 kb
63,180 kb
63,200 kb
63,220 kb
63,240 kb
63,260 kb
63,280 kb
63,300 kb
63,320 kb
HPRT3
HPRT2
HPRT1
BCL2
BCL2

Figure S2

Table S4. Genes includes on the Illumina L-300 liquid panel. All coding exons in the genes are represented in the panel.

ABCC9	BRCA1	CHEK1	DNMT3B	FAT3	HIST1H3D	KIT	MIR21	NSD2	RAD21	SGK1	TET2	ZMYM2
ABL1	BRCA2	CHK2	EBF1	FBXW7	HIST1H4D	KLHL6	MIR34B	NT5C2	RAD51	SH2B3	TGDS	ZMYM3
ACTG1	BRIP1	CIITA	ECT2L	FGFR3	HNRNPK	KRAS	MIR34C	PAG1	RAD51C	SHH	TINF2 (TIN2)	ZRSR2
AKT1	BTG1	CNOT3	EED	FLI1	HRAS	LAMB4	MLL	PALB2	RAG1	SMAD2	TLR2	
ANKRD11	BTK	CREBBP	EGR1	FLT3	ICOS	LEF1	MLL2	PAX5	RAG2	SMC1A	TLR9	
ARID1A	BTLA	CRLF2	EGR2	FNDC3A	ID3	LRP1B	MLL3	PDCD1	RASA2	SMC3	TNFAIP3	
ARID1B	C22orf194	CSF2RA	ELANE	FOXP1	IDH1	LTB	MPL	PDCD1LG2	RB1	SMC5	TNFRSF14	
ARID2	CALR	CSF3R	EP300	FYN	IDH2	LUC7L2	MRE11A	PDGFRB	REL	SNX7	TNKS	
ARID5B	CARD11	CTBP1	EPHA7	G6PC3	IKBKA	LYN	MS4A1	PEG3	RELA	SOCS1	TOX	
ARPP21	CBL	CTBP2	EPOR	GAB2	IKZF1	MALT1	MYB	PHF6	RELB	SOX5	TP53	
ASXL1	CBLB	CTCF	ERG	GATA1	IKZF2	MAP2K1	MYD88	PHIP	RELN	SP140	TRAF2	
ATF7IP	CCND1	CTLA4	ETV6	GATA2	IKZF3	MAP3K14	NBN	PIGA	RHOA	SPEN	TRAF3	
ATM	CCND3	CTNNA1	EZH2	GATA3	IL7R	MAPK1	NCOR1	PIK3CA	RIPK1	SPIB	TRAF6	
ATRX	CD200	CUL5	FAM46C	GCET2	IRAK1	MAX	NCOR2	PIK3CB	ROBO1	SRSF2	TYK2	
B2M	CD274	CUX1	FAM5C	GFI1B	IRAK4	MDM2	NF1	PIK3CG	ROR1	STAG1	TYK3	
BCL10	CD58	CYLD	FANCA	GNA13	IRF1	MED12	NFE2	PIK3R1	RPL10	STAG2	U2AF1	
BCL2	CD79A	DAXX	FANCB	GNAS	IRF4	MEF2B	NFKB1	PLA2G2D	RPL5	STAT1	U2AF2	
BCL6	CD79B	DCLRE1C	FANCC	GNB1	IRF7	MEF2C	NFKB2	PLCG2	RUNX1	STAT3	UBR5	
BCL7A	CDK4	DDX3X	FANCD2	GPRC5A	ITPKB	MGA	NFKBIA	POT1	RUNX2	SUZ12	USP29	
BCOR	CDKN2A	DIS3	FANCE	H2AFX	JAK1	MIR125a	NFKBIE	POU2AF1	SAMHD1	SYK	VPREB1	
BCR	CDKN2B	DKC1	FANCG	HAX1	JAK2	MIR-142	NOTCH1	PRDM1	SETBP1	TBL1XR1	WHSC1	
BIRC3	CDKN2C	DLC1	FANCI	HIST1H1E	JAK3	MIR155	NOTCH2	PRKCB	SETD2	TCF3	WHSC1L1	
BLK	CEBPA	DNM2	FANCL	HIST1H2AD	JARID2	MIR15a	NPM1	PTEN	SF3B1	TERC	WT1	
BMI1	CEBPE	DNMT1	FAS	HIST1H2BE	KDM4C	MIR16-1	NR3C2	PTPN1	SFRS1	TERT	XPO1	
BRAF	CHD2	DNMT3A	FAT1	HIST1H2BF	KDM6A	MIR17HG	NRAS	PTPN11	SFRS7	TET1	ZAP70	

HPRT3 mutations		
**Gene**	**Standardized Nomenclature (HGVS)**	**VAF (%)**
**KRAS**	KRAS_I187V, K182R	99.62%, 46.15%
**CHD2**	CHD2_V253G	99.60%
**SMAD2**	SMAD2_I93V	99.36%
**JAK2**	JAK2_PGS275/276/277SAR	99.00%
**PIK3CB**	PIK3CB_P384Q	98.93%
**LEF1**	LEF1_V235G	98.90%
**KIT**	KIT_E306K	98.30%
**STAG1**	STAG1_S108L	97.70%
**PIGA**	PIGA_I228V	97.48%
**PTEN**	PTEN_T398S	97.22%
**PPP2R5D**	PPP2R5D_F141C	96.88%
**TOX**	TOX_D17A	96.63%
**PRKCB**	PRKCB_V325I	96.60%
**PAX5**	PAX5_Q285L	96.30%
**FBXW7**	FBXW7_P23A	95.58%
**SHH**	SHH_D246E,R258L,	97.4%, 97.2%
**RUNX1**	RUNX1_A36G	90.00%
**PIK3CA**	PIK3CA_S66T	88.89%
**FAM46C**	FAM46C_S369N	85.71%
**RAD51**	RAD51_N268D	85.71%
**SF3B1**	SF3B1_R108S	85.00%
**ANKRD11**	ANKRD11_A91G	84.62%
**RELN**	RELN_S718A	83.33%
**CHUK**	CHUK_S249T	82.61%
**ATM**	ATM_I159V	81.82%
**CTNNA1**	CTNNA1_A92V	81.82%
**RAG2**	RAG2_H166Q	81.25%
**CHEK2**	CHEK2_A258E	79.00%
**FAT3**	FAT3_N1995S	50.00%
**CYLD**	CYLD_QP366/367Q	50.00%
**BTG1**	BTG1_R86W,S128L, K162R	11.03%
**CBL**	CBL_V535I	95.00%
**BCL6**	BCL6_T696A	94.74%
**CTLA4**	CTLA4_P121S	94.55%
**RB1**	RB1_P793S	93.46%
**DNM2**	DNM2_I417L	90.91%
**MLL**	MLL_P4L,PV45/46QA, T170A,L989P,T993A,N1814S,S3295G,P3301Q,L3305LP	100%, 90.48%, 99%,94.87%,100%, 95.71%,94.37, 92.75% 100%
**SUZ12**	SUZ12_V31A, T35A, S58/SAA,	100%, 100%, 85.3%

Table S5

HPRT3 mutations		
**Gene**	**Standardized Nomenclature (HGVS)**	**VAF (%)**
**LUC7L2**	LUC7L2_Q6H, Q43R, R48H, C348R	99.7%,97.4%, 85.7%,99.6%
**SPEN**	SPEN_G158A, GG110/111SA	99.68%,77%
**FAM5C**	FAM5C_N21S, A22V,G30D, M757V	99.42%,80%,98.8%,98.3%
**MEF2C**	MEF2C_C116S,N389S,D394G,T412TT	99.4%, 99.4%, 98.6%, 93.8%
**STX16**	STX16_N20T,I32T	99.3%,99.2%
**SMC5**	SMC5_N851S,L854P	99.2%,99%
**IKZF2**	IKZF2_E98D,G271A,P340A,A373S,H417R,H423N,T446A,	99.2%,95.7%,100%, 98.4%,97.4%,98.8%, 94.7%
**WHSC1L1**	WHSC1L1_N37H, A41V, V625A, I640N, N894S, K916R, L1186F	99.2%,85.7%,99.4%,96.6%,92.5%,96.8%, 98.7%
**FYN**	FYN_T12A,IA245/246VS,R266G,C269F,	99.2%,100%, 60%,84%
**EPHA7**	EPHA7_I209V,I214V	99.1,99.7%
**MGA**	MGA_N1387T, C1402S,E1427D,A1459S, S1472T, T1509P, P1514S	99%, 99%,99%,98%, 88%, 96%, 87%
**LRP1B**	LRP1B_Y548C, M2086L,A3240V, Y3253N	99%, 90%,96.9%,94.7%
**IKZF1**	IKZF1_I125V,TL235/236M	98.9%87%
**ATRX**	ATRX_TE154/155AD,T1303i, S2301T,N2303T,P2462S,M2479TV,Q2488P	98.7,, 98.7%, 40%,99.2%, 99.3%,75.7%, 96.4%, 96.5%, 99.2
**SOX5**	SOX5_A13T,D32E,H67P	98.64%,94.67%,95.83%
**ARID2**	ARID2_F19S, L67S, S1103N,P1131Q,S1137A,Q1835*	98.39%,66.67%, 80.95%,91.67%,92.11%, 81.82%
**MAX**	MAX_RG19/20DQ, S120G,AR51/52VQ	97.9%,96%,86%
**RUNX2**	RUNX2_S23C, S26A, V28A, T58H, K63E, T355S, G452A,	97.3%,90.4%,50%,98.4%,93.2%, 100%, 99.1%
**STAG2**	STAG2_R298S, N841S	96.3%, 87%
**EGR2**	EGR2_V18M, S22P,N24S,I25L,	95.57%, 87.93%,53.33%,41.67%
**IRF4**	IRF4_I49V, V233I	94.4%, 100%
**CREBBP**	CREBBP_GE49/50E, M328L, T462A, QT513/514PA	94.3%,91.3%,100%, 83.3%
**RAD21-AS1**	RAD21-AS1_P6A, L10R	93.3%, 93.6%
**MLL3**	MLL3_P12Q, P17A,A1142V,C3833G,T3846N	91.7%,97.5%, 97.7%, 96%,95.2%
**FOXP1**	FOXP1_L159F, C322R, N325H, H334Y	72.7%, 93.3%,92.9%, 85.7%
**MYB**	MYB_T331S, P508L	60%,81.8%
**NCOR1**	NCOR1_S1414P,PPL1418/1420L,A1800S, T1870N,L1881V,Y1882C, S1896A	58%,50%,100%,99.6%,90%,90%,97%
**RHOA**	RHOA_D146E, E186K	17.7%,99.2%

HPRT3 mutations		
**Gene**	**Standardized Nomenclature (HGVS)**	**VAF (%)**
**SETD2**	SETD2_M45V,D1055E	91.7%,86.7%
**BCOR**	BCOR_S228G,G1034S,M1043V,YS1188/1189CN	91.7%, 99.6%, 98.9%, 99.8%
**FGFR3**	FGFR3_V321A, S354R	91.7%, 97.9%
**MED12**	MED12_S63N, N987S	91.5%,90.9%
**WHSC1**	WHSC1_S147G,P634S	90.9%,76.9%
**ZMYM2**	ZMYM2_V319I,I1293V	87.5%,88.89%
**CUX1**	CUX1_K426R, S475T, S475Y,M516I, Q531P	87.5%, 93.3%, 92.3%,75%,66.7%
**LYN**	LYN_L19S, E28D	86.7%,96.5%
**PRDM1**	PRDM1_P168A,P804H	86.4%,64.7%
**ARID1A**	ARID1A_EA38/39E-, T118P, P153A,AP226/227PQ,S317G,A349V, S1197P, Q1579P,N2044D, Q2273H	80.95%, 100%,100%, 97%, 100%,100%, 98.28%, 98.41%, 93.33%12.14%
**UBR5**	UBR5_S982N, A1032S, I1845V	80%,95.8%, 87%
**SETBP1**	SETBP1_N926T, H992Q,I1185L	80%,69%,67%
**JARID2**	JARID2_A346T,D359E,R688K	78.6%,85.3%,100%
**EP300**	EP300_T140S,ST150/151SPA,S2095P,S2400A	77.8%,87%,92.3%,88%
**MLL2**	MLL2_A1161T,Y1169YG,L1599V,T2323A,P2558S,L3619V	75%,75%, 92.86%,93.33%,88.24%,63.64%
**XPO1**	XPO1_L27F,S961P,I972M,L974I,	72.7%,93.6%,81.3%72.7%
**ASXL1**	ASXL1_S526P, A530E, S534P, I598V, T600I	100%,98.5%,98.8%,95.5%, 94.7%
**ZMYM3**	ZMYM3_VS296/297MA, T324M, G334D,G345E,D898E	100%,100%,97.5%, 97.5%, 97.5%,98.3%
**NFKBIA**	NFKB1A_E125G, G132R	100%, 97.9%
**NF1**	NF1_K1385R,T2805A	100%, 94.1%
**ARID1B**	ARID1B_P228S,G278S,C295Y, G360S, A363S, AP445/446A, M479L, M479I,A2183T	100%, 100%, 100%,100%, 100%,84.6%, 96.2%,95.2%
**KDM6A**	KDM6A_T10TAA, S120P, P263T, I288V, I295V, L312P,A325T,Q344P,S348L,I421T,H430Q,M451V, T452M,T491P, A503T, M563I, S960N	>90%
**CCND1**	CCND1_V94L	100.00%
**CEBPA**	CEBPA_A9V	100.00%
**U2AF2**	U2AF2_P347L	100.00%

Table S5

HPRT2 mutations		
**Gene**	**Standardized Nomenclature (HGVS)**	**VAF (%)**
**TP53**	TP53_P72R	100.00%
**TET2**	TET2_I1783V	99.66%
**SPEN**	SPEN_G158A, R844*	11.86%, 99.56%
**CRLF2**	CRLF2_R207C	60.61%
**BCR**	BCR_F378L	48.77%
**BRCA1**	BRCA1_K1183R	48.54%
**ATM**	ATM_F858L	47.63%
**IL7R**	IL7R_T244I	47.04%
**NOTCH2**	NOTCH2_Y2392*, C19W, A21T	46.62%, 28.57%, 15.15%
**HIST1H1E**	HIST1H1E_A123V	46.32%
**BCL10**	BCL10_E140*	45.56%
**TBL1XR1**	TBL1XR1_SC324/325C	43.08%
**IRF4**	IRF4_G58S,G58D	42.17%, 42.42%
**KDM6A**	KDM6A_T10TAA, S120P,I288V,I295V,L312P, A325T,Q344P,S348L,I421T,H430Q,M451 V,T452M,T491P,M563I,	21.36%,29.83%, 19.21%,23.29%, 21.70%,15.63%,19.41%,21.96, 34.32%,26.51%,26.63%,27.01%, 16.92%,6.12%
**MLL3**	MLL3_P17A,Y987H,C988F,S990G	6.69%,40.72%,22.76%,9.21%
**MGA**	MGA_N1387T, C1402S,E1427D,A1459S	6.24%, 25.04%, 28.46%, 11.33%
**BCOR**	BCOR_G1034S,M1043V,YS1188/1189C N	25.65%,25.68%,13.45%
**NF1**	NF1_K1385R	23.65%
**ARID1B**	ARID1B_P228S,G278S,C295Y, AP445/446A, M479L, M479I	17.70%,19.8%,19.57%,8.33%,5. 62%, 19.98%
**IKZF2**	IKZF2_E98D,A373S	5.84%, 16.53%
**LUC7L2**	LUC7L2_Q6H, C348R	16.36%, 11.72%
**STX16**	STX16_N20T,I32T	19.41%,19.72%
**ATRX**	ATRX_TE154/155AD,S2301T,N2303T,P2 462S,M2479TV,Q2488P	19%, 10.32%, 10%,7.34%,11.87%,9.42%
**IKZF1**	IKZF1_I125V	12.19%
**CEBPA**	CEBPA_A9V	11.21%
**ARPP21**	ARPP21_G243S	11.17%
**PIGA**	PIGA_I228V	10.08%

HPRT2 mutations		
**Gene**	**Standardized Nomenclature (HGVS)**	**VAF (%)**
**JARID2**	JARID2_R688K	10.07%
**RHOA**	RHOA_E186K	9.87%
**CHD2**	CHD2_V253G	9.82%
**KRAS**	KRAS_D38/-	9.14%
**PIK3CB**	PIK3CB_P384Q	9.04%
**NCOR2**	NCOR2_1840/1841-	9.02%
**STAG2**	STAG2_N841S	8.46%
**LRP1B**	LRP1B_Y548C	8.05%
**WHSC1L1**	WHSC1L1_V625A	7.94%
**ITPKB**	ITPKB_S/92SG	7.62%
**MED12**	MED12_P1949S	7.46%
**EGR2**	EGR2_V18M	7.18%
**BTG1**	BTG1_S43R	6.86%
**STAG1**	STAG1_S108L	6.53%
**SOX5**	SOX5_A13T	5.84%
**ARID2**	ARID2_F19S	5.62%
**SUZ12**	SUZ12_VA31/32AV, T35A, S58/SAA,	6.5%, 5.7%,9.7%
**NCOR1**	NCOR1_A1800S, T1870N	6.15%,8.12%
**FYN**	FYN_T12A,IA245/246VS	5.57%, 7.2%
**RUNX2**	RUNX2_S23C, T355S	5.33%,8.06%
**ASXL1**	ASXL1_S534P, G1397S	5.31%, 44.93%
**ARID1A**	ARID1A_EA38/39E-, T118P, P153A,AP226/227PQ,S317G,A349V	15.62%,9.89%,12%, 12.3% 10.5%
**MEF2C**	MEF2C_C116S,N389S,D394G,T412TT	14.21%,17.86%,12.08%,14.61 %
**MAX**	MAX_RG19/20DQ, S120G	10%, 9.15%
**MLL**	MLL_PV45/46QA, AA61/62-	10%, 8.77%
**ZMYM3**	ZMYM3_G334D,G345E	7.78%,6.15%
**SHH**	SHH_D246E,R258L,	7.61%,10.2%
**SMC5**	SMC5_N851S,L854P	7.51%,9.04%
**FAM5C**	FAM5C_N21S, G30D	6.52%, 6.87%
**EPHA7**	EPHA7_I209V,I214V	9.05%,5.72%
**FGFR3**	FGFR3_S354R	5.13%

Table S6

HPRT1 mutations		
**Gene**	**Standardized Nomenclature (HGVS)**	**VAF (%)**
**BRCA1**	BRCA1_K1183R	99.5%
**TP53**	TP53_I232N	99.4%
**ATM**	ATM_L1715V	61.7%
**TET2**	TET2_I1783V	47.2%
**DCLRE1C**	DCLRE1C_T437I	46.4%
**MLL3**	MLL3_C988F, _Y987H, G845E,	45.4%, 15.6%, 8.2%
**NOTCH2**	NOTCH2_A21T, C19W, N46S,	39.2%, 16.7%, 6.1%
**FAT3**	FAT3_Y2248S	19.0%
**CTBP1**	CTBP1_H405P, H408P	10.5%, 5.9%
**NCOR2**	-	22.7 %,
**BCR**	BCR_T1127S	8.0%
**DKC1**	DKC1_K498-	5.8%
**LUC7L2**	-	5.3%
**PDE4D**	-	5.1%

Table S7

Table S8A: Top 25 Super Enhancers determined by ROSE analysis in HPRT3 DLBCL cells

Super Enhancer Rank	Gene	Chromosome	5’ end of Super Enhancer	3’ end of Super Enhancer	Size of Super enhancer (bp)
1	IRF8	chr16	85921018	86053033	118288
2	ENTPD1	chr10	97489010	97603415	66679
3	DAD1	chr14	22916513	23039635	113129
4	TOMM20	chr1	234962564	235192209	143613
5	VOPP1	chr7	55535647	55643995	107058
6	TMSB4X	chrX	12964953	13113746	102832
7	CYTOR	chr2	87739311	87909576	126964
8	IRF2	chr4	185184232	185402620	157102
9	SUSD1	chr9	114653053	114844287	116969
10	RPLP1	chr15	69848015	70058546	121288
11	IGLL5	chr22	23190746	23296934	90022
12	PPIF	chr10	80999148	81057449	36659
13	ELK2AP	chr14	106090085	106245324	75126
14	CCDC68	chr18	52673190	52808411	77029
15	TMEM121	chr14	106024623	106074650	48982
16	MIR4435-2HG	chr2	112123122	112309031	146999
17	TRIB1	chr8	126555361	126661470	61247
18	CLCN4	chrX	10043233	10178241	86598
19	RAD51B	chr14	68714923	68886484	111164
20	CLLU1	chr12	92788189	92901557	83710
21	EHMT1	chr9	140567384	140670470	71165
22	ARID5B	chr10	63699450	63844173	83137
23	TCF4	chr18	52876435	52996067	93451
24	ASB2	chr14	94406171	94471640	55131
25	MSI2	chr17	55481284	55640658	106600

Table S8B: Top 25 Super Enhancers determined by ROSE analysis in HPRT2 DLBCL cells

Super Enhancer Rank	Gene	Chromosome	5' end of Super Enhancer	3' end of Super Enhancer	Size of Super enhancer (bp)
1	HGSNAT	chr8	43092765	43097158	3714
2	TMEM121	chr14	106024353	106053791	29286
3	ELK2AP	chr14	106142085	106177043	30548
4	IGLL5	chr22	23240937	23298213	53841
5	EMBP1	chr1	121458768	121485451	2250
6	BCL2	chr18	60779129	60903231	65256
7	VOPP1	chr7	55528433	55641592	73438
8	CMTM7	chr3	32432518	32520249	65897
9	THADA	chr2	43561459	43693127	79136
10	BTG2	chr1	203240729	203310140	51529
11	SH3BP5	chr3	15296453	15449463	78395
12	MTSS1	chr8	125604759	125660667	46306
13	TGFBR2	chr3	30637130	30739196	61206
14	PLCG2	chr16	81806884	81918840	64101
15	ZNF595	chr4	9967	61884	31853
16	CD44	chr11	35084826	35209483	57763
17	CLDND1	chr3	98237380	98293303	46116
18	RHEX1	chr1	206252822	206310416	44034
19	FOXP1	chr3	71039006	71185002	59747
20	ATP2B1	chr12	90252967	90361534	49719
21	LOC101927151	chr19	27731907	27740347	4638
22	ACTR3BP2	chr2	92272960	92326157	19669
23	ENTPD1	chr10	97501031	97605005	58645
24	OSBPL10	chr3	31916506	32079804	65916
25	CYTH1	chr17	76661606	76778307	66710

Table S8C: Top 25 Super Enhancers determined by ROSE analysis in HPRT1 DLBCL cells

Super Enhancer Rank	Gene	Chromosome	5’ end of Super Enhancer	3’ end of Super Enhancer	Size of Super enhancer (bp)
1	MYC	chr8	129054681	129219222	136458
2	BCL6	chr3	187587946	187857441	154427
3	POU2AF1	chr11	111218186	111317279	61086
4	PDGFD	chr11	103732531	104042785	134460
5	CDK6	chr7	92390887	92469816	57211
6	DAD1	chr14	22849151	23039739	127997
7	SH3KBP1	chrX	19611695	19932461	181623
8	RFTN1	chr3	16366547	16578125	144026
9	CADPS	chr3	62609710	62830894	115284
10	PTPRJ	chr11	47921141	48135402	114209
11	FUT8	chr14	65748436	65852386	60600
12	IGLL5	chr22	23152153	23303452	97747
13	ITPKB	chr1	226814412	226930087	79560
14	GRHPR	chr9	37360209	37419499	47177
15	CASC8	chr8	128568131	128592536	15792
16	WEE1	chr11	9586225	9776663	111634
17	PLCG2	chr16	81803475	81922958	79581
18	MSH6	chr2	47867681	48003920	51874
19	CPEB4	chr5	173116701	173262046	82168
20	MIR3681HG	chr2	12132887	12271588	96382
21	PTPRC	chr1	198564755	198685367	90322
22	RAPGEF5	chr7	22308280	22500172	98657
23	BFSP2/CDV3	chr3	133143825	133298129	62108
24	FCHSD2	chr11	72843363	72901191	35686
25	NCOA3	chr20	46006211	46250350	88248

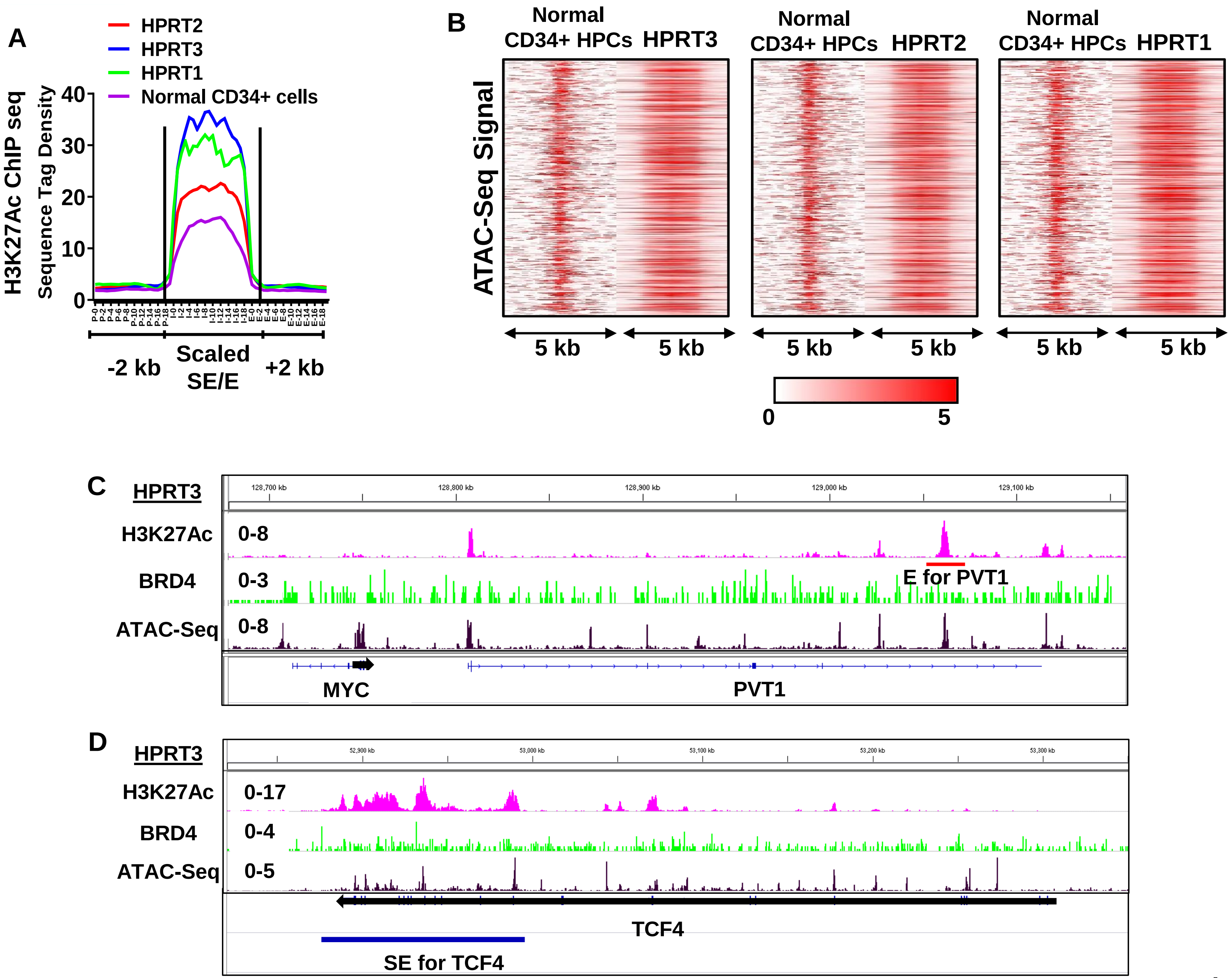
A
HPRT2
HPRT3
HPRT1
Normal CD34+ cells
H3K27Ac ChIP seq
Sequence Tag Density
40
30
20
10
0
-2 kb
Scaled SE/E
+2 kb
B
Normal CD34+ HPCs
HPRT3
Normal CD34+ HPCs
HPRT2
Normal CD34+ HPCs
HPRT1
ATAC-Seq Signal
5 kb
5 kb
5 kb
5 kb
5 kb
5 kb
0
5
C
HPRT3
H3K27Ac
0-8
BRD4
0-3
ATAC-Seq
0-8
E for PVT1
MYC
PVT1
D
HPRT3
H3K27Ac
0-17
BRD4
0-4
ATAC-Seq
0-5
TCF4
SE for TCF4

Figure S3

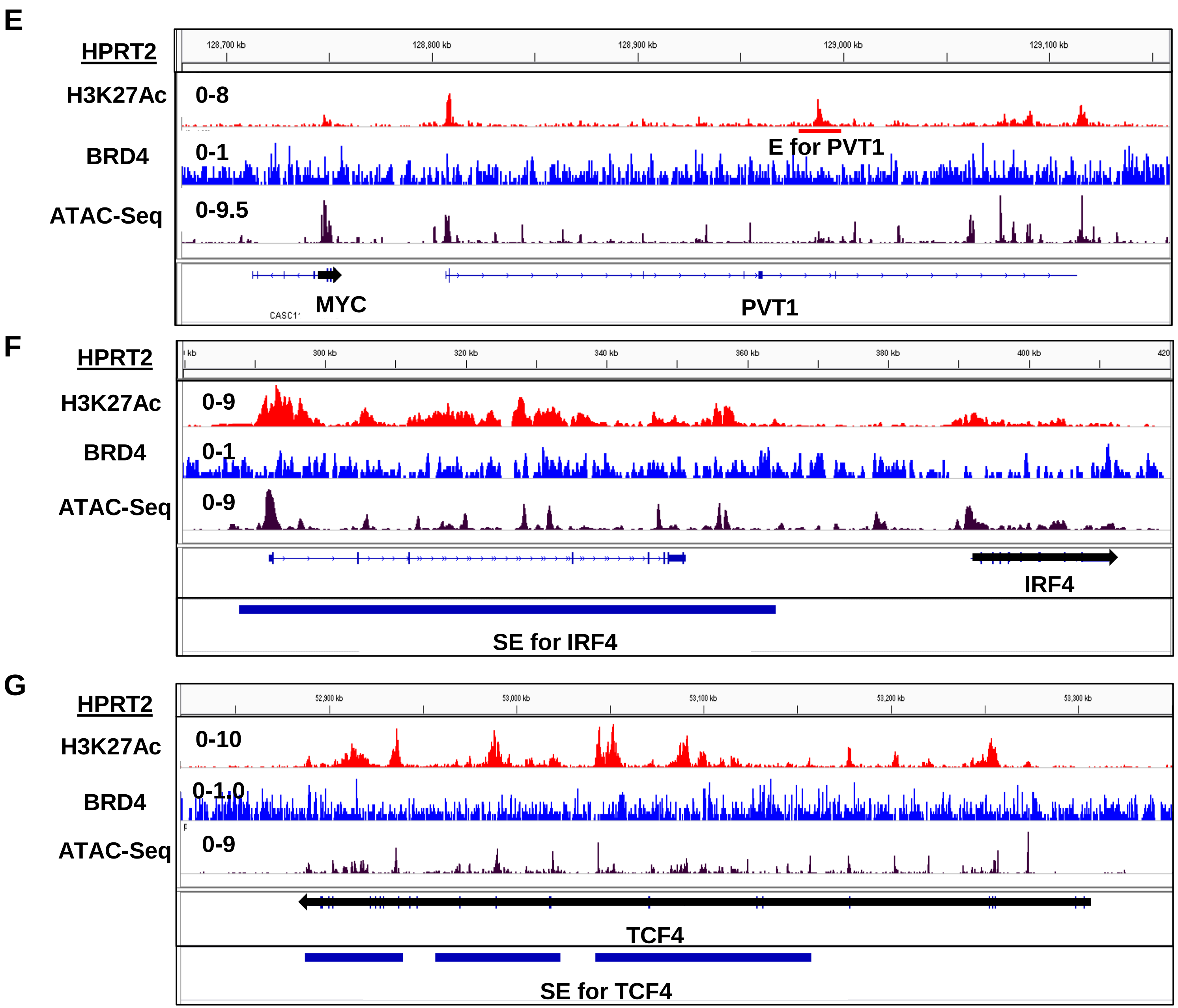
E
HPRT2
128,700 kb
128,800 kb
128,900 kb
129,000 kb
129,100 kb
H3K27Ac
0-8
E for PVT1
BRD4
0-1
ATAC-Seq
0-9.5
MYC
PVT1
F
HPRT2
300 kb
320 kb
340 kb
360 kb
380 kb
400 kb
H3K27Ac
0-9
BRD4
0-1
ATAC-Seq
0-9
IRF4
SE for IRF4
G
HPRT2
52,900 kb
53,000 kb
53,100 kb
53,200 kb
53,300 kb
H3K27Ac
0-10
BRD4
0-1.0
ATAC-Seq
0-9
TCF4
SE for TCF4

Figure S3

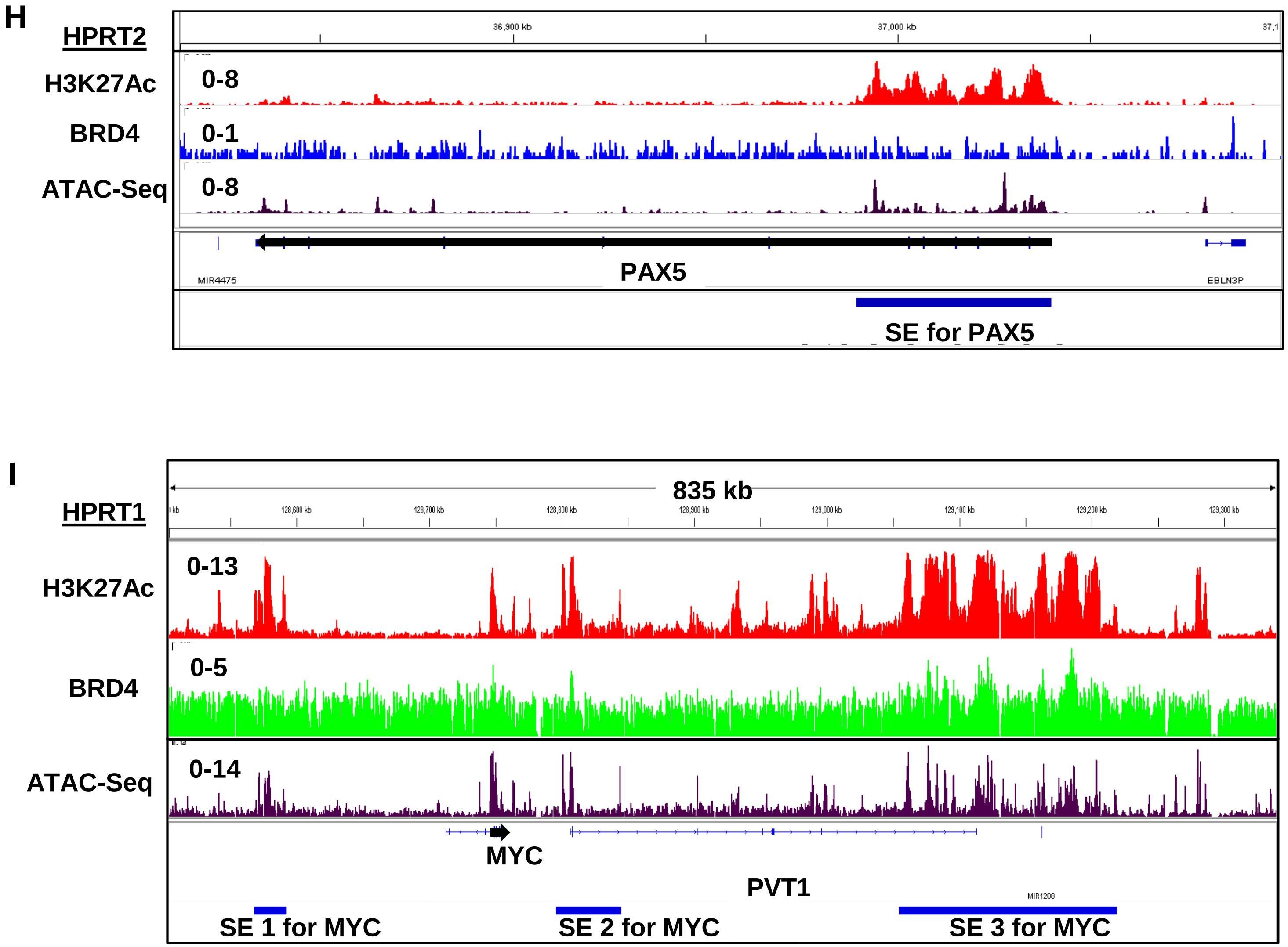
H
HPRT2
H3K27Ac
BRD4
ATAC-Seq
36,900 kb
37,000 kb
0-8
0-1
0-8
MIR4475
PAX5
EBLN3P
SE for PAX5
I
HPRT1
835 kb
128,600 kb
128,700 kb
128,800 kb
128,900 kb
129,000 kb
129,100 kb
129,200 kb
129,300 kb
H3K27Ac
BRD4
ATAC-Seq
0-13
0-5
0-14
MYC
PVT1
MIR1208
SE 1 for MYC
SE 2 for MYC
SE 3 for MYC

Figure S3

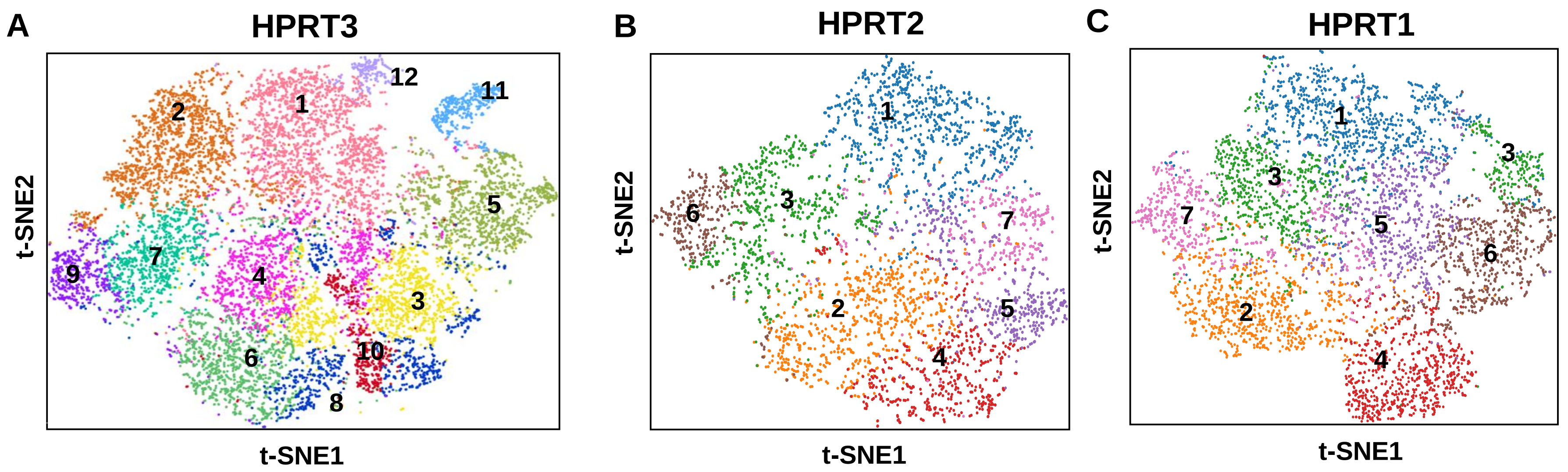

HPRT3

Cluster number	# of cells per cluster
1	2300
2	1731
3	1456
4	1314
5	1226
6	1217
7	931
8	913
9	503
10	441
11	438
12	207

HPRT2

Cluster number	# of cells per cluster
1	878
2	769
3	606
4	502
5	454
6	294
7	288

HPRT1

Cluster number	# of cells per cluster
1	1129
2	963
3	929
4	772
5	733
6	709
7	562

Figure S4

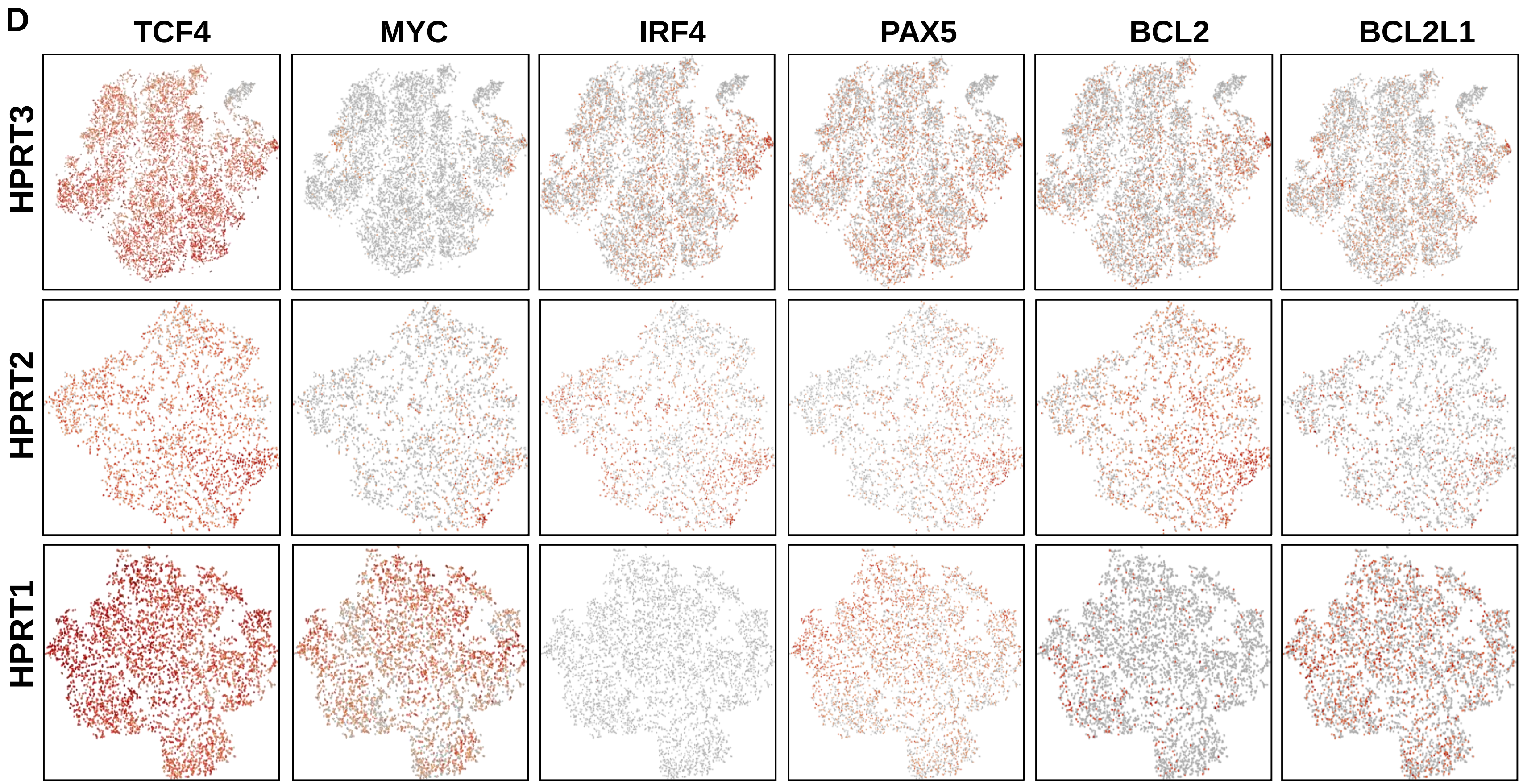

Figure S4

E

Baseline RNA expression (t-SNE plots) in HPRT3 cells following single cell RNA sequencing (10X genomics)

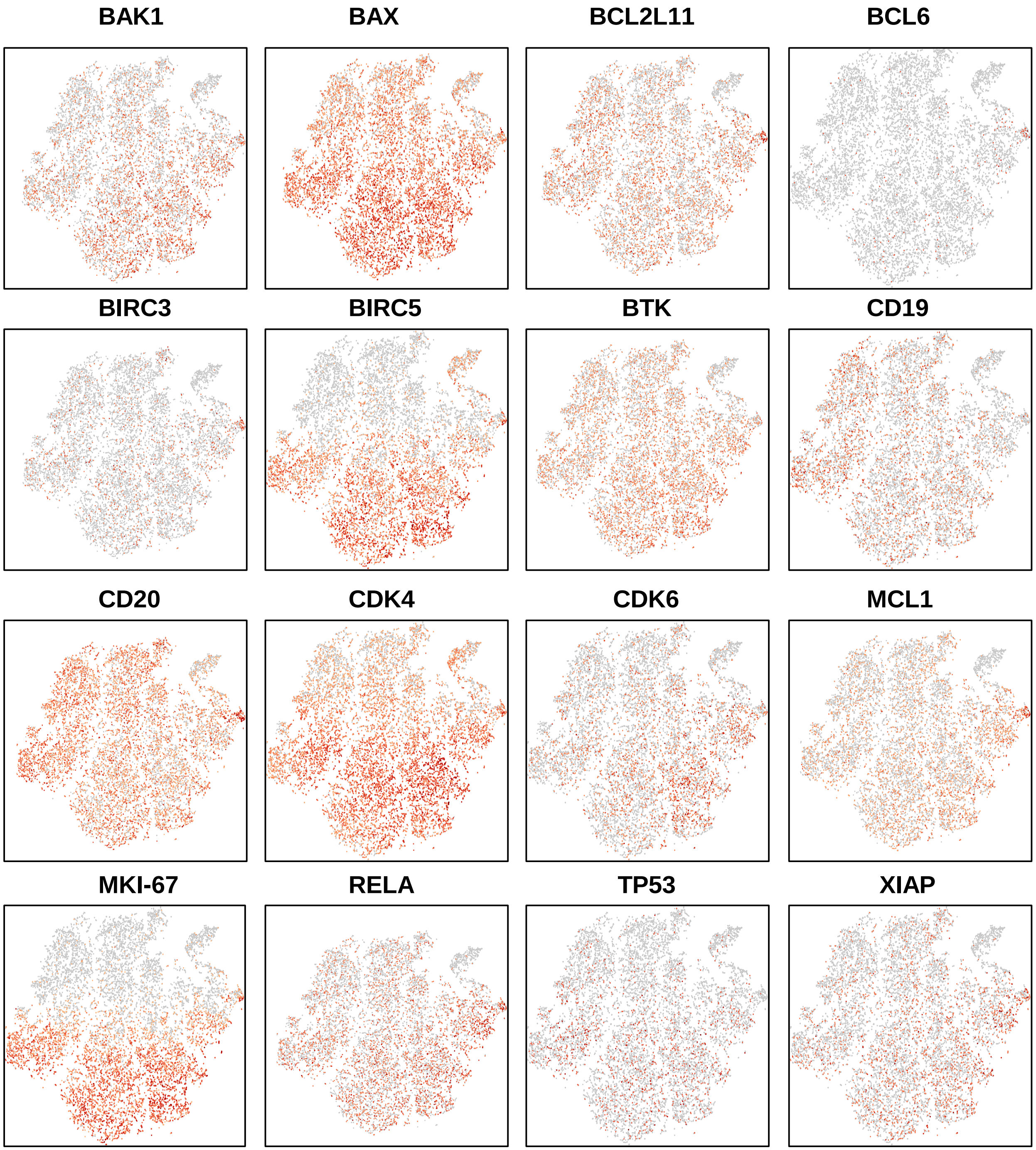

Figure S4

F

Baseline RNA expression (t-SNE plots) in HPRT2 cells following single cell RNA sequencing (10X genomics)

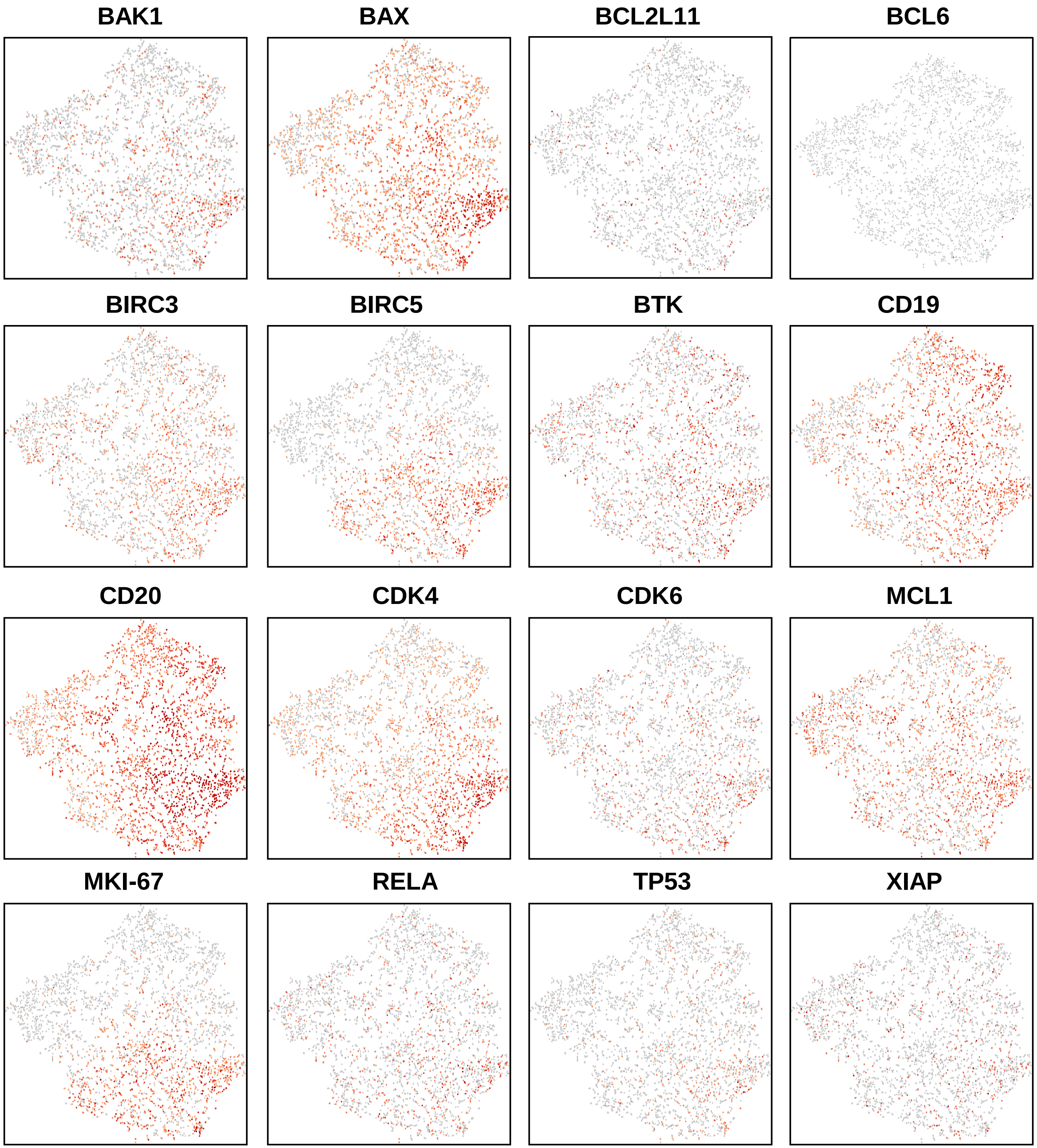

Figure S4

G

Baseline RNA expression (t-SNE plots) in HPRT1 cells following single cell RNA sequencing (10X Genomics)

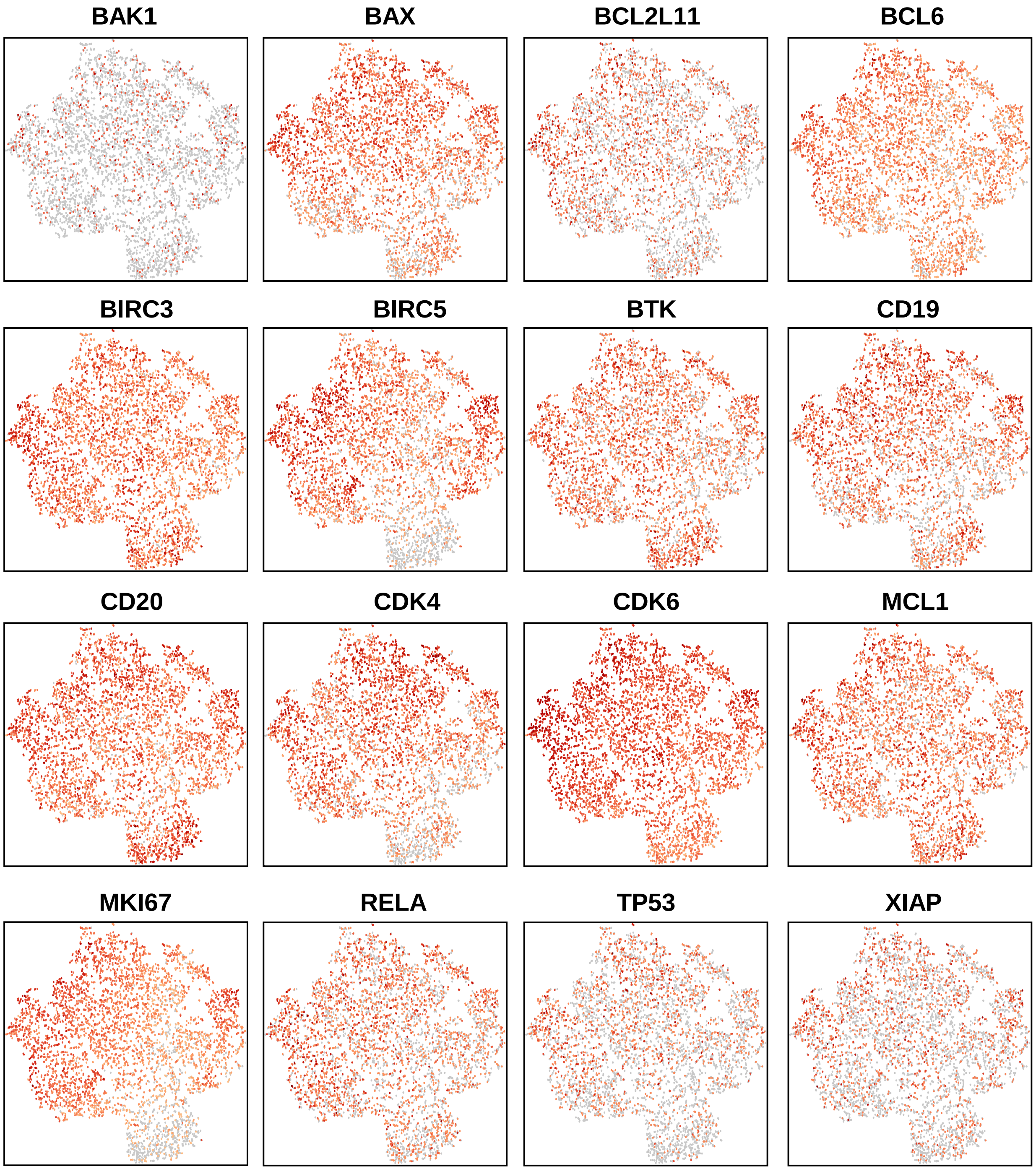

Figure S4

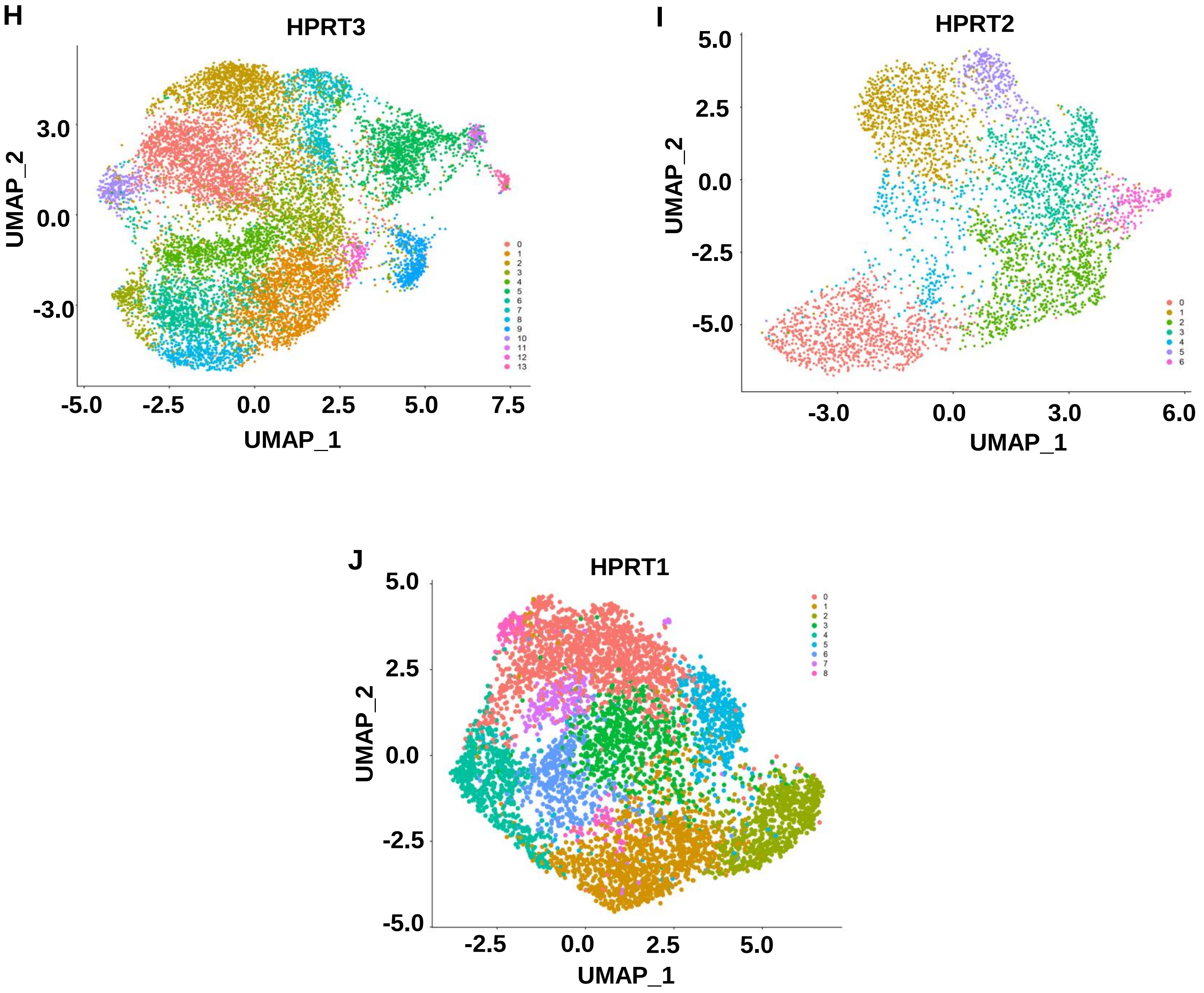
H
HPRT3
3.0
0.0
-3.0
UMAP_2
-5.0
-2.5
0.0
2.5
5.0
7.5
UMAP_1
I
HPRT2
5.0
2.5
0.0
-2.5
-5.0
UMAP_2
-3.0
0.0
3.0
6.0
UMAP_1
J
HPRT1
5.0
2.5
0.0
-2.5
-5.0
UMAP_2
-2.5
0.0
2.5
5.0
UMAP_1

Figure S4

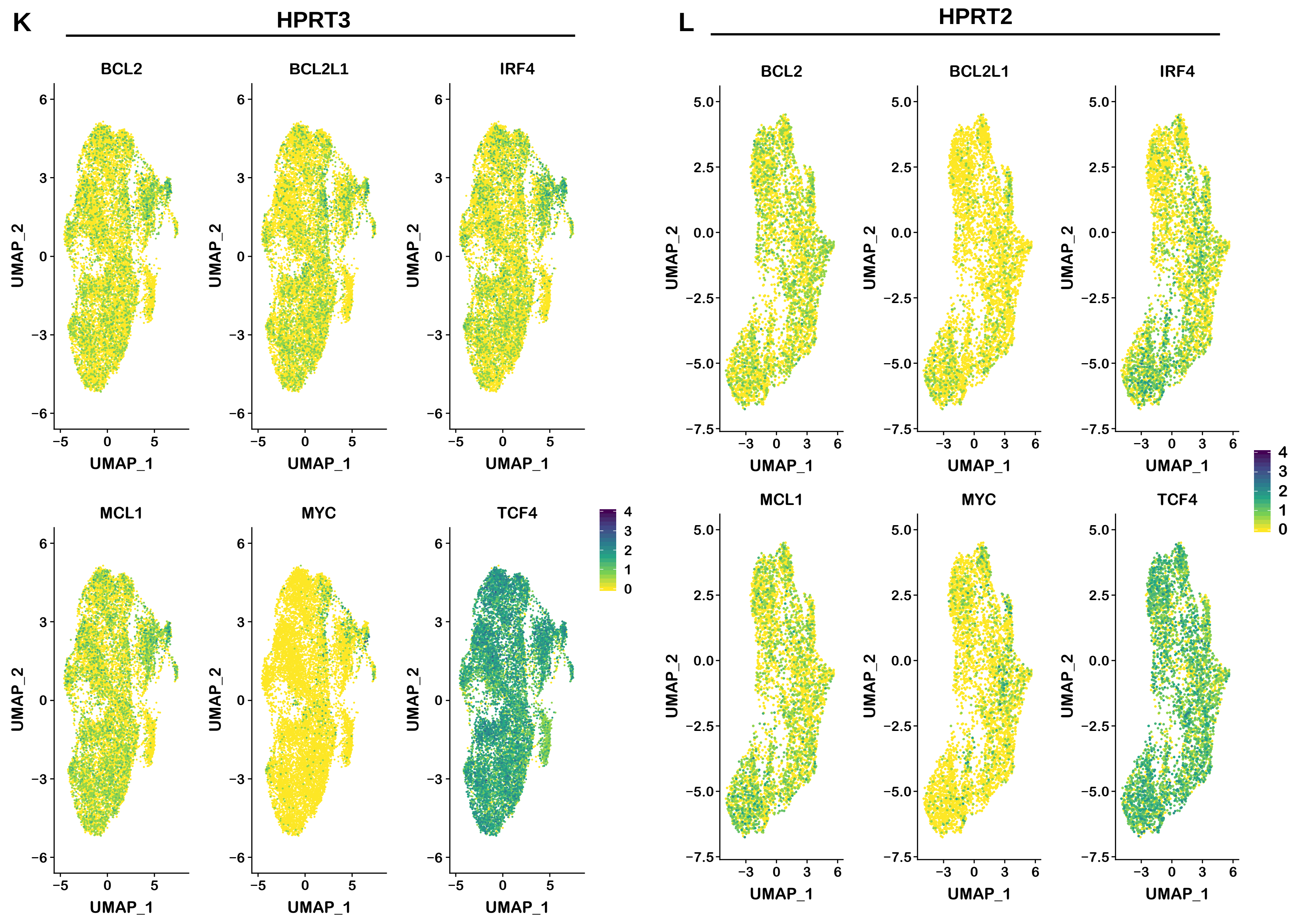

K
HPRT3
BCL2
BCL2L1
IRF4
MCL1
MYC
TCF4
UMAP_1
UMAP_2
L
HPRT2
4
3
2
1
0

Figure S4

M

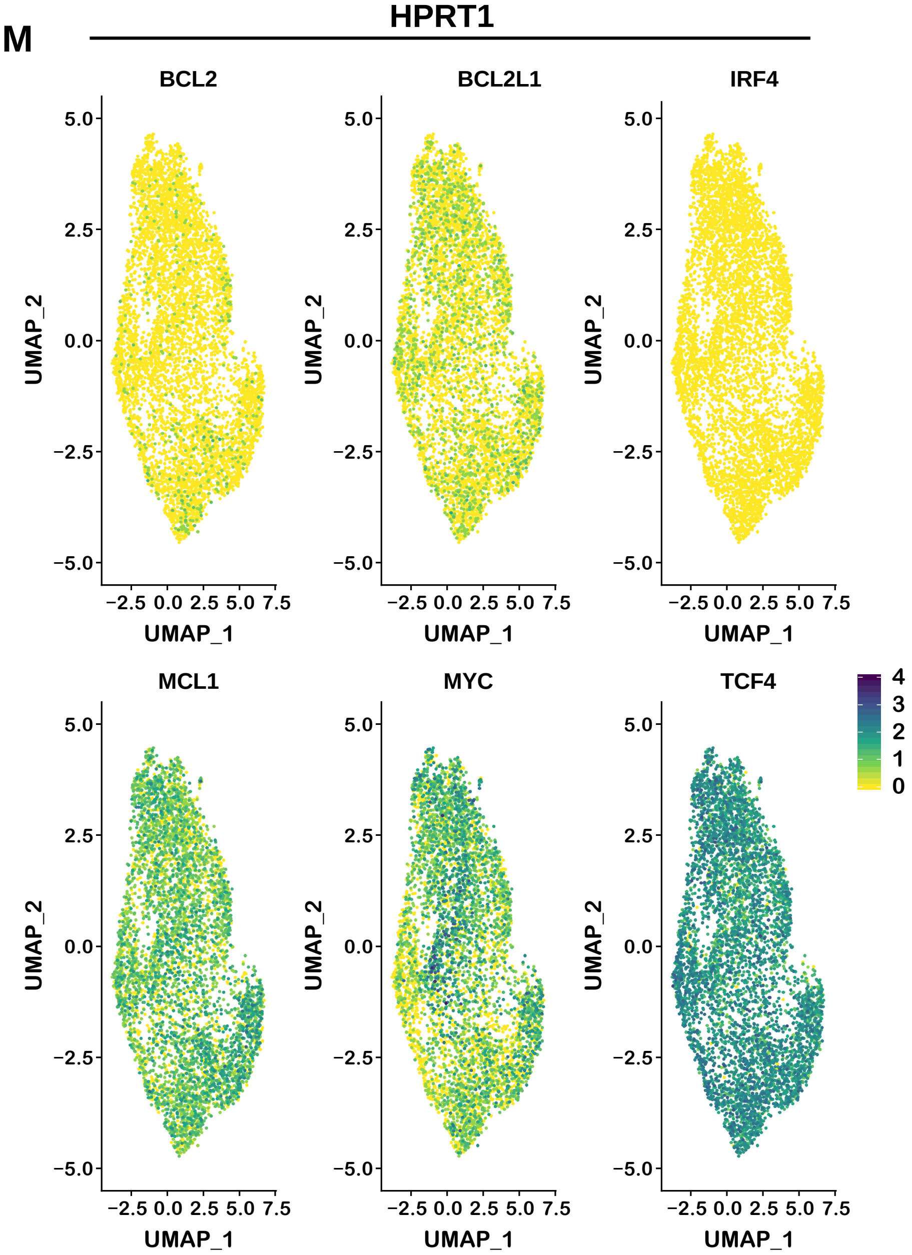
HPRT1
BCL2
BCL2L1
IRF4
MCL1
MYC
TCF4
UMAP_2
UMAP_1
5.0
2.5
0.0
−2.5
−5.0
−2.5 0.0 2.5 5.0 7.5
4
3
2
1
0

Figure S4

Table S9. Individual cluster marker genes for single cell RNA-Seq data in HPRT3, HPRT2 and HPRT1 DLBCL cells.

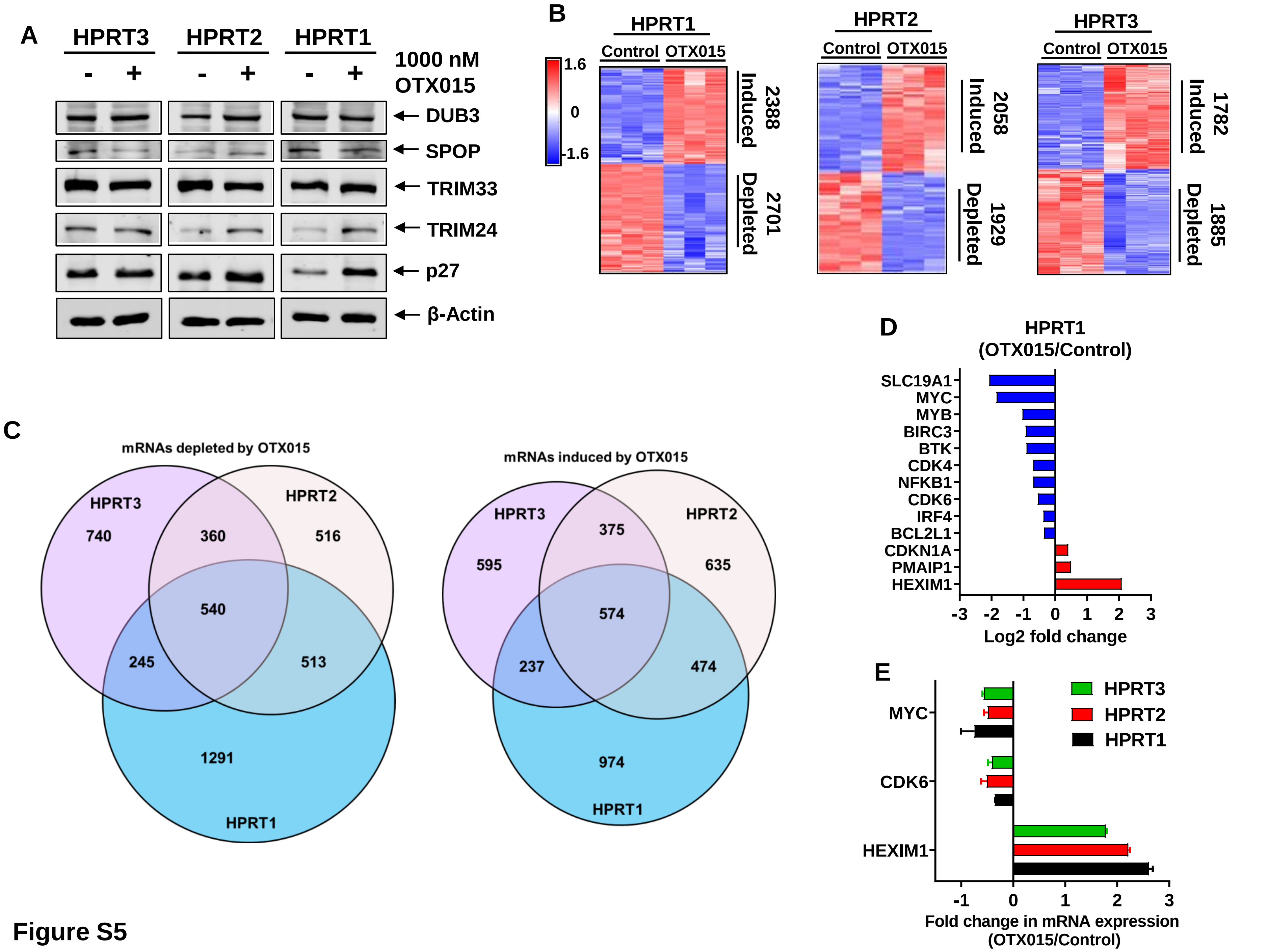
A
HPRT3
HPRT2
HPRT1
1000 nM OTX015
- + - + - +
DUB3
SPOP
TRIM33
TRIM24
p27
β-Actin
B
HPRT1
HPRT2
HPRT3
Control OTX015
1.6
0
-1.6
2388 Induced
2701 Depleted
2058 Induced
1929 Depleted
1782 Induced
1885 Depleted
C
mRNAs depleted by OTX015
HPRT3 740
HPRT2 516
360
540
245
513
1291
HPRT1
mRNAs induced by OTX015
HPRT3 595
HPRT2 635
375
574
237
474
974
HPRT1
D
HPRT1 (OTX015/Control)
SLC19A1
MYC
MYB
BIRC3
BTK
CDK4
NFKB1
CDK6
IRF4
BCL2L1
CDKN1A
PMAIP1
HEXIM1
-3 -2 -1 0 1 2 3
Log2 fold change
E
HPRT3
HPRT2
HPRT1
MYC
CDK6
HEXIM1
-1 0 1 2 3
Fold change in mRNA expression (OTX015/Control)

Figure S5

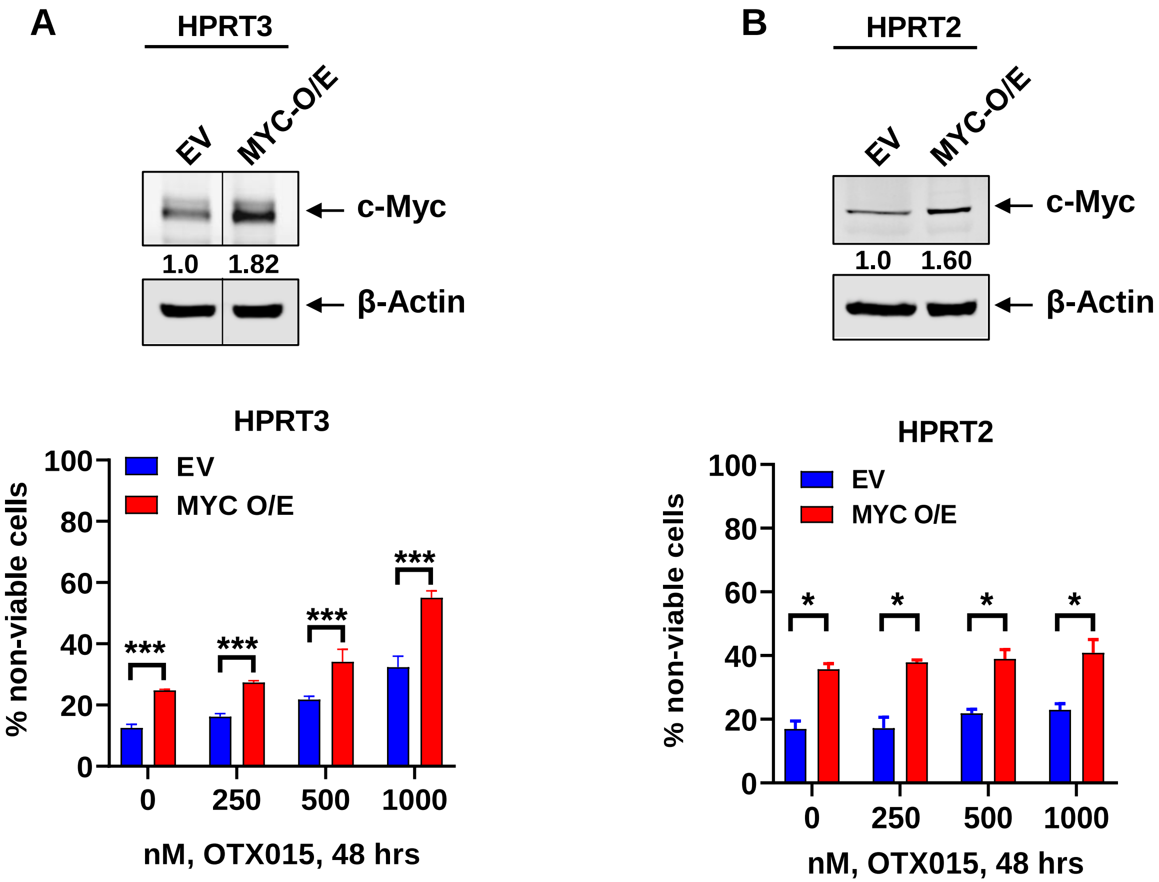
A
HPRT3
EV
MYC-O/E
c-Myc
1.0
1.82
β-Actin
HPRT3
% non-viable cells
EV
MYC O/E
***
nM, OTX015, 48 hrs
B
HPRT2
EV
MYC-O/E
c-Myc
1.0
1.60
β-Actin
HPRT2
% non-viable cells
EV
MYC O/E
*
nM, OTX015, 48 hrs

Figure S6

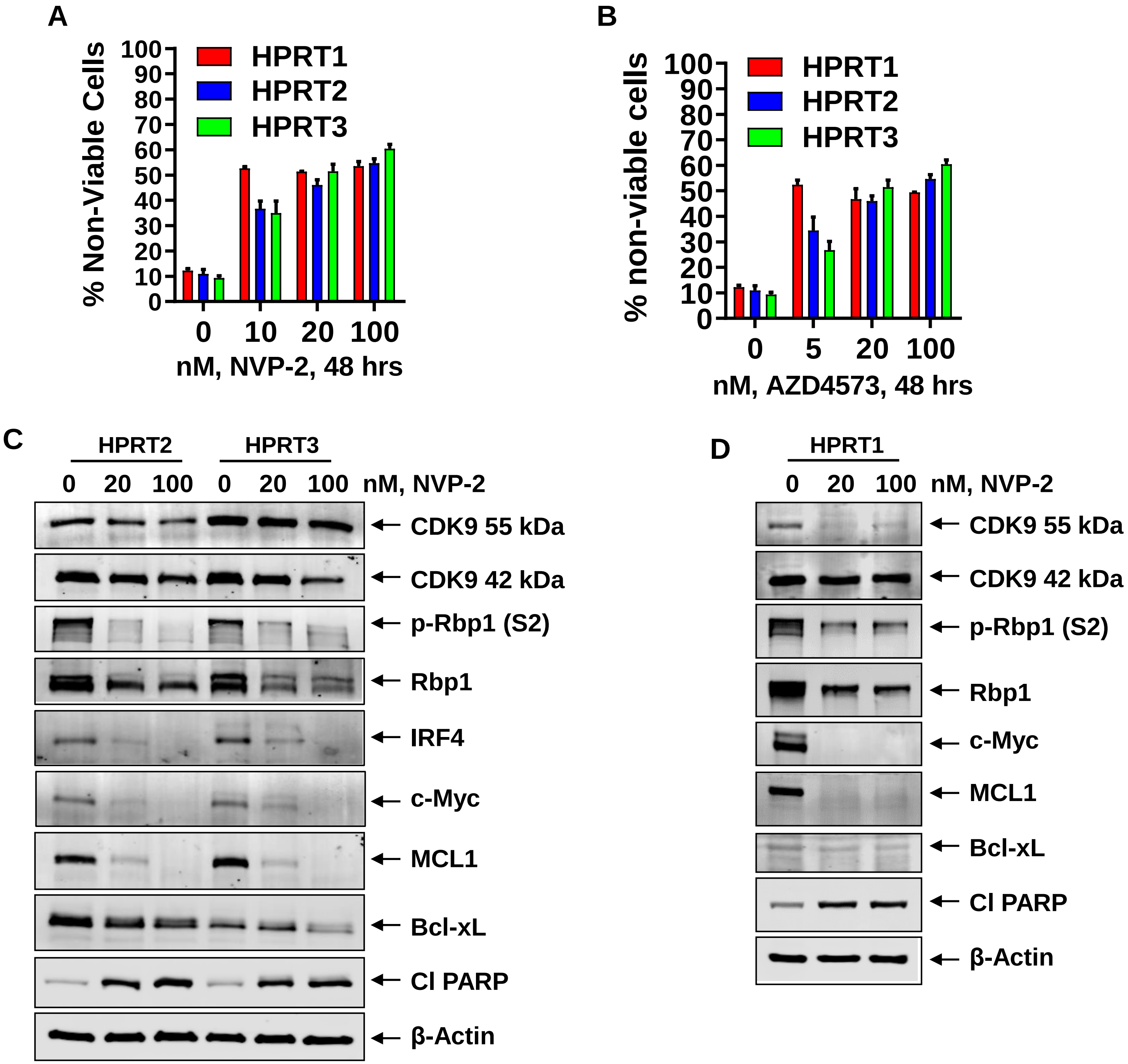
A
% Non-Viable Cells
HPRT1
HPRT2
HPRT3
0 10 20 100
nM, NVP-2, 48 hrs
B
% non-viable cells
HPRT1
HPRT2
HPRT3
0 5 20 100
nM, AZD4573, 48 hrs
C
HPRT2
HPRT3
0 20 100 0 20 100 nM, NVP-2
CDK9 55 kDa
CDK9 42 kDa
p-Rbp1 (S2)
Rbp1
IRF4
c-Myc
MCL1
Bcl-xL
Cl PARP
β-Actin
D
HPRT1
0 20 100 nM, NVP-2
CDK9 55 kDa
CDK9 42 kDa
p-Rbp1 (S2)
Rbp1
c-Myc
MCL1
Bcl-xL
Cl PARP
β-Actin

Figure S7

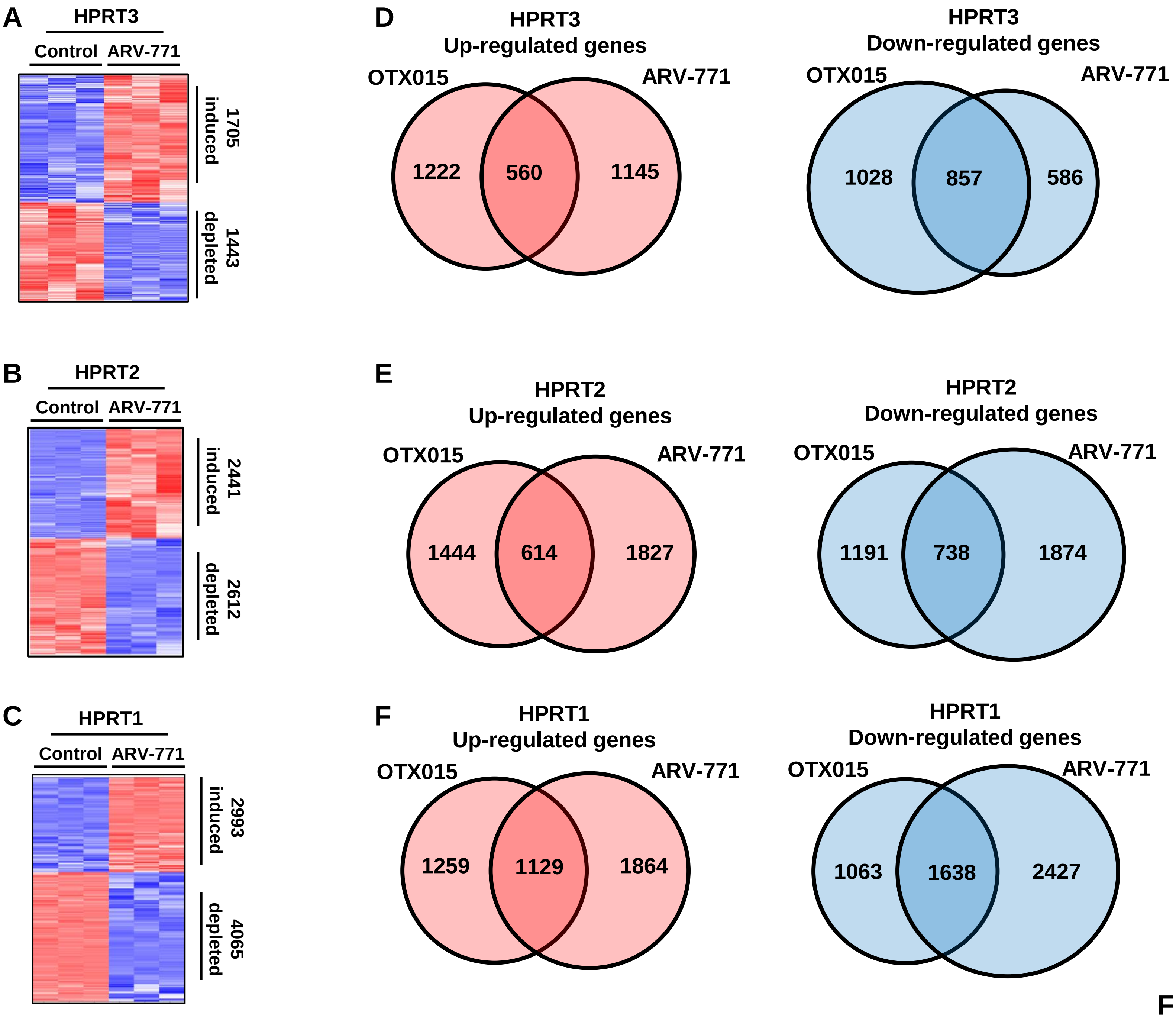

A
HPRT3
Control
ARV-771
1705 induced
1443 depleted
B
HPRT2
Control
ARV-771
2441 induced
2612 depleted
C
HPRT1
Control
ARV-771
2993 induced
4065 depleted
D
HPRT3
Up-regulated genes
OTX015
ARV-771
1222
560
1145
HPRT3
Down-regulated genes
OTX015
ARV-771
1028
857
586
E
HPRT2
Up-regulated genes
OTX015
ARV-771
1444
614
1827
HPRT2
Down-regulated genes
OTX015
ARV-771
1191
738
1874
F
HPRT1
Up-regulated genes
OTX015
ARV-771
1259
1129
1864
HPRT1
Down-regulated genes
OTX015
ARV-771
1063
1638
2427

Figure S8

Table S10. Sequences of oligonucleotide primers and TaqMan probe identifiers utilized in these studies.

Primers or TaqMan probes utilized	
**Primer Name**	**Primer Sequence (5'-3')**
**EBV For**	**CTT GGA GAC AGG CTT AAC CAG ACT CA**
**EBV Reverse**	**CCA TGG CTG CAC CGA TGA AAG TTA T**
**Control For**	**GAG GAA GCA GTC CTT AGT TTCATG**
**Control Rev**	**GTA CTT ATT GAA TCT CAG TCT GTC AGT TAC**
**MYC**	**Hs00153408_m1**
**CDK6**	**Hs01026371_m1**
**HEXIM1**	**Hs00538918_s1**
**GAPDH**	**Hs99999905_m1**

Table S10

A

Table S11. Absolute loss of viability for RT-DLBCL cells treated with BET inhibitor or BET PROTAC and ibrutinib or venetoclax for synergy analysis

HPRT3

OTX015 (nM)	Ibrutinib (μM)	Fractional Effect	CI
250.0	2.0	0.4677	0.09538
500.0	2.0	0.4793	0.17705
1000.0	2.0	0.5064	0.29759
250.0	10.0	0.5071	0.07406
500.0	10.0	0.5044	0.15071
1000.0	10.0	0.5238	0.26614

HPRT2

OTX015 (nM)	Ibrutinib (nM)	Fractional Effect	CI
100.0	500.0	0.253	0.37856
200.0	1000.0	0.266	0.39096
300.0	1500.0	0.301	0.23836
400.0	2000.0	0.324	0.22680
500.0	2500.0	0.342	0.22454

HPRT1

OTX015 (nM)	Ibrutinib (nM)	Fractional Effect	CI
50.0	50.0	0.412	0.48147
100.0	100.0	0.634	0.41959
200.0	200.0	0.904	0.18028
300.0	300.0	0.937	0.17916
400.0	400.0	0.985	0.06332
500.0	500.0	0.985	0.07915

B

HPRT3

OTX015 (nM)	venetoclax (nM)	Fractional Effect	CI
250.0	20.0	0.4289	0.35171
500.0	20.0	0.4737	0.36828
1000.0	20.0	0.5247	0.46794
250.0	50.0	0.4408	0.50538
500.0	50.0	0.4873	0.41458
1000.0	50.0	0.4965	0.64822
250.0	100.0	0.5109	0.29695
500.0	100.0	0.531	0.34396
1000.0	100.0	0.5042	0.71126

HPRT2

OTX015 (nM)	venetoclax (nM)	Fractional Effect	CI
100	50	0.257	0.1467
200	100	0.304	0.15159
300	150	0.293	0.26349
400	200	0.341	0.18849
500	250	0.359	0.18872

HPRT1

OTX015 (nM)	venetoclax (nM)	Fractional Effect	CI
50.0	50.0	0.227	0.61176
100.0	100.0	0.361	0.62973
200.0	200.0	0.575	0.52504
300.0	300.0	0.689	0.48056
400.0	400.0	0.618	0.87790

Table S11

C

HPRT3

ARV-771 (nM)	Ibrutinib (μM)	Fractional Effect	CI
50.0	2.0	0.5033	0.04750
100.0	2.0	0.5266	0.08177
250.0	2.0	0.5344	0.19440
50.0	10.0	0.5817	0.02856
100.0	10.0	0.5605	0.06565
250.0	10.0	0.5616	0.16294

HPRT2

ARV-771 (nM)	Ibrutinib (μM)	Fractional Effect	CI
20.0	2.0	0.3006	0.44292
50.0	2.0	0.7436	0.11288
100.0	2.0	0.7464	0.22181
20.0	10.0	0.4294	0.22666
50.0	10.0	0.8305	0.06029
100.0	10.0	0.8172	0.13455

HPRT1

ARV-771 (nM)	Ibrutinib (nM)	Fractional Effect	CI
2.0	50.0	0.713	0.42112
4.0	100.0	0.85	0.54197
8.0	200.0	0.987	0.28788
12.0	300.0	0.996	0.24021
16.0	400.0	0.997	0.27807
20.0	500.0	0.995	0.44691

Table S11

D

HPRT3

ARV-771 (nM)	Venetoclax (nM)	Effect	CI
50.0	20.0	0.3575	0.79680
100.0	20.0	0.4437	0.20702
250.0	20.0	0.5221	0.12479
50.0	100.0	0.5294	0.13880
100.0	100.0	0.4985	0.26674
250.0	100.0	0.5157	0.26008

HPRT2

ARV-771 (nM)	Venetoclax (nM)	Effect	CI
50.0	20.0	0.6092	0.01775
100.0	20.0	0.6202	0.01497
250.0	20.0	0.5638	0.03527
50.0	50.0	0.5612	0.09159
100.0	50.0	0.6144	0.04095
250.0	50.0	0.6234	0.03561
50.0	100.0	0.578	0.14252
100.0	100.0	0.6145	0.08178
250.0	100.0	0.5944	0.11126

HPRT1

ARV-771 (nM)	Venetoclax (nM)	Effect	CI
2.0	50.0	0.466	0.56141
4.0	100.0	0.789	0.56261
8.0	200.0	0.872	0.84631
12.0	300.0	0.956	0.73186
16.0	400.0	0.967	0.84660
20.0	500.0	0.979	0.84885

Table S11

Table S12: ChIP-Seq, ATAC-Seq and RNA-Seq sample Details and sequencing reads.

A

ChIP Sample	Number of sequencing reads
HPRT1 H3K27Ac ChIP	106,851,766
HPRT1 BRD4 ChIP	45,498,342
HPRT1 input	77,247,054
HPRT2 H3K27Ac ChIP	50,679,274
HPRT2 BRD4 ChIP	75,665,078
HPRT2 input	55,031,692
HPRT3 H3K27Ac ChIP	56,429,718
HPRT3 BRD4 ChIP	77,856,094
HPRT3 input	47,599,658

B

ATAC-Seq Samples	Number of sequencing reads
HPRT1 ATAC 1	138,296,734
HPRT1 ATAC 2	133,543,246
HPRT2 ATAC 1	95,236,150
HPRT2 ATAC 2	99,336,576
HPRT3 ATAC 1	58,437,328
HPRT3 ATAC 2	20,308,784

C

RNA-Seq Sample	Number of sequencing reads
HPRT1 Control 1	37,052,615
HPRT1 Control 2	34,468,023
HPRT1 Control 3	32,690,982
HPRT1 OTX015 1	34,266,702
HPRT1 OTX015 2	35,950,707
HPRT1 OTX015 3	45,779,630
HPRT1 ARV-771 1	27,331,677
HPRT1 ARV-771 2	23,977,473
HPRT1 ARV-771 3	26,819,429

RNA-Seq Sample	Number of sequencing reads
HPRT2 Control 1	30,772,821
HPRT2 Control 2	32,100,379
HPRT2 Control 3	34,815,791
HPRT2 OTX015 1	27,801,771
HPRT2 OTX015 2	30,881,955
HPRT2 OTX015 3	39,225,265
HPRT2 ARV-771 1	33,106,448
HPRT2 ARV-771 2	36,503,776
HPRT2 ARV-771 3	24,506,326

RNA-Seq Sample	Number of sequencing reads
HPRT3 Control 1	33,905,220
HPRT3 Control 2	36,557,945
HPRT3 Control 3	39,410,712
HPRT3 OTX015 1	38,621,342
HPRT3 OTX015 2	35,320,047
HPRT3 OTX015 3	40,106,214
HPRT3 ARV-771 1	32,753,015
HPRT3 ARV-771 2	30,915,987
HPRT3 ARV-771 3	36,637,739
