# Supplementary material for: BET proteolysis targeted chimera-based therapy of novel models of Richter Transformation-diffuse large B-cell lymphoma
Source: Leukemia. 2021 Mar 2;35(9):2621–34. doi: 10.1038/s41375-021-01181-w (PMC8410602; doi:10.1038/s41375-021-01181-w)
Supplement: Supplementary file 2 — Supplemental Figure Legends [file 41375_2021_1181_MOESM2_ESM.docx]

**Supplemental Figure Legends**

**Figure S1. HPRT3, HPRT2, and HPRT1 cells are negative for Epstein–Barr virus (EBV) and EBNA2 protein.** **A**. Tabulated cell cycle status of HPRT3, HPRT2, and HPRT1 cells (n=3) shown in Fig.1B. **B**. FISH analysis of the MYC locus in HPRT1 cells. An LSI MYC dual‐color break apart probe set (Abbott Laboratories) was used to assess rearrangements involving the MYC locus on chromosome 8q24. **C**. Immuno-histochemical evaluation of IRF4, CD10 and BCL6 in HPRT3, HPRT2 and HPRT1 cells. **D**. PCR primers were designed to detect the presence of EBV DNA in mammalian cells. RNA-free genomic DNA was isolated from HPRT3, HPRT2, and HPRT1 cells and utilized for PCR analysis to detect EBV DNA. Genomic DNA from Granta-519 cells (an EBV-positive MCL cell line) was used as a positive control. **E**. Immunoblot analysis of EBNA2 protein in HPRT2, HPRT3, and HPRT1. Granta-519 total cell lysate was utilized as a positive control for EBNA2 protein expression. The expression levels β-Actin in the lysates served as the loading control.

**Table S1. Type of tissue specimen and characteristics of immunostaining for the 52 cases of RT analyzed for expression of PAX5, IRF4, CD5, CD10, BCL6, and CD30**. In the table, (1) indicates positive staining, (0) indicates negative staining, and (D) indicates dim staining.

**Table S2. Immuno-histochemical analysis of PAX5, IRF4, CD5, CD10, BCL6, and CD30 expression in tissues from 52 cases of Richter Transformation.** Tabulated results of immuno-histochemical analysis of PAX5, IRF4, CD5, CD10, BCL6, and CD30 expression in tissues from 52 cases of Richter Transformation.

**Table S3. Clonal relationship of HPRT3, HPRT2, and HPRT1 RT-DLBCL patient-derived xenograft (PDX) models to antecedent CLL.** Immunoglobulin heavy chain (IGH) status and somatic hypermutation (SH) was utilized to establish the clonal relationship of HPRT3, HPRT2, and HPRT1 DLBCL cells with their antecedent CLL. FR= Framework regions of the VH segment of the IGH gene.

**Figure S2. Karyotype, array CGH analysis and low-pass whole-genome sequencing for global copy gains and losses in the three RT-DLBCL cell types**. **A**. HPRT3, HPRT2, and HPRT1 cells were karyotyped to detect chromosome level abnormalities. Detected chromosomal abnormalities are presented in the table above a representative karyotype for each RT-DLBCL sample. **B**. Genomic DNA was analyzed from HPRT3, HPRT2, and HPRT1 cells by array CGH analysis. The copy gains and losses in each RT-DLBCL are shown. **C-D**. Low-pass whole-genome sequencing of genomic DNA-identified copy gains in the TCF4 and BCL2 genes on chromosome 18 in HPRT3 and HPRT2 but not HPRT1 cells. Integrated Genome Viewer (IGV) plots show regions of increased copy gains (Red) and copy losses (Blue) in the HPRT3, HPRT2, and HPRT1 cells.

**Table S4. Genes included on the Illumina L-300 liquid panel. All coding exons in the genes are represented in the panel.**

**Table S5. All mutations identified by the L300 next generation sequencing panel in HPRT3 cells**.

**Table S6. All mutations identified by the L300 next generation sequencing panel in HPRT2 cells**.

**Table S7. All mutations identified by the L300 next generation sequencing panel in HPRT1 cells**.

**Table S8. Top 25 super enhancers identified by ROSE analysis in HPRT3, HPRT2, and HPRT1 cells.** **A-C**. H3K27Ac ChIP-Seq density was utilized to conduct ranked ordering of super enhancer (ROSE) analysis. The table shows the ranked order of the top 25 super enhancers, the gene associated with each super enhancer, and the predicted size of each super enhancer in the three RT-DLBCL cell types.

**Figure S3. Analysis of H3K27Ac and BRD4 occupancy by ChIP-Seq and chromatin accessibility by ATAC-Seq in HPRT3, HPRT2, and HPRT1 cells**. **A**. Sequence-tag density plot of H3K27Ac at scaled SEs in the RT-DLBCL cells compared to publicly available H3K27Ac ChIP-Seq density in normal CD34+ HPCs (GSM772870, GSM772885, and GSM772894). H3K27Ac sequence tag densities are different in the three RT cell types. They are greatest within the SE/E, compared to the 2-kb upstream and downstream. **B**. ATAC-Seq analysis was performed on the nuclei from RT-DLBCL cells to determine chromatin accessibility. Publicly available ATAC-Seq data from normal CD34+ HPCs were downloaded from GEO (GSE18927) for comparison. The heat maps show the ATAC-signal density from the center of the peaks +/- 2.5 kb upstream and downstream in the RT cells compared to normal CD34+ HPCs. **C-D.** Integrated Genome Viewer (IGV) plots showing H3K27Ac and BRD4 signal density and ATAC-Seq-determined chromatin accessibility in the MYC, PVT1 and TCF4 genes in clonally-related HPRT3-DLBCL cells. **E-H.** IGV plots showing H3K27Ac and BRD4 signal density and ATAC-Seq determined chromatin accessibility in the MYC, PVT1, IRF4, TCF4, and PAX5 genes in clonally unrelated HPRT2-DLBCL cells. The blue bars beneath the signal tracks indicate the position of the super enhancer for IRF4, TCF4, and PAX5 as determined by ROSE analysis. **I**. IGV plot showing H3K27Ac and BRD4 signal density and ATAC-Seq determined chromatin accessibility within the MYC gene and in the adjacent PVT1 gene in HPRT1 cells. The blue bars beneath the signal tracks indicate the positions of super enhancers for MYC in HPRT1 cells.

**Figure S4. Baseline RNA expressions in HPRT3, HPRT2, and HPRT1** **cells as determined by single-cell RNA-Seq analysis. A.** HPRT3, HPRT2, and HPRT1 cells were separated into single cell droplets utilizing a 10X Genomics chromium separator instrument. Single-cell RNA was reverse transcribed and cDNA libraries were constructed. Sequencing of the single-cell cDNA libraries was performed on a HiSeq4000 sequencer. Sequence reads were processed in Cell Ranger and entered in the Loupe Cell Browser for generation of t-SNE plots based on similar RNA expression levels in the clustered cells. The tables indicate the number of clusters and the number of cells per cluster for HPRT3, HPRT2, and HPRT1 cells. **B-D.** Selected t-SNE plots of baseline mRNA expression in HPRT3, HPRT2, and HPRT1 cells following single-cell RNA-Seq analysis. **E-G**. UMAP plots showing the number of clusters in HPRT3, HPRT2 and HPRT1 based on similar RNA expression levels. **H-J.** UMAP feature plots showing relative mRNA expression of BCL2, BCL2L1, IRF4, MYC, MCL1, and TCF4 at single-cell resolution in HPRT3, HPRT2, and HPRT1 cells. Green indicates strong positive expression of an mRNA; yellow indicates low or absent mRNA expression.

**Figure S5. Treatment with BET inhibitor OTX015 induces global mRNA expression alterations including depletion of c-Myc, CDK4/6, and Bcl-xL with concomitant induction of HEXIM1 in RT-DLBCL cells**. **A**. HPRT3, HPRT2, and HPRT1 cells were treated with OTX015 as indicated for 24 hours. At the end of treatment, total cell lysates were prepared and immunoblot analyses were conducted for DUB3, SPOP, TRIM33, TRIM24, and p27. The expression levels β-Actin in the lysates served as the loading control. **B**. HPRT1, HPRT2, and HPRT3 cells were treated with 1000 nM of OTX015 for eight hours. Total RNA was harvested and utilized for mRNA-Seq analysis. The heat map shows the number of induced and depleted genes with a fold-change (fc) of >1.5 and a p-value < 0.05. **C**. Three-way Venn diagrams comparing the overlaps in OTX015-mediated depletion or induction of mRNAs, as determined by RNA-Seq analysis, in HPRT3, HPRT2, and HPRT1 cells. **D**. Log2 fold-changes of selected, significantly altered (>1.5 fold change and p-value < 0.05) mRNAs from the RNA-Seq in HPRT1 are shown. **E**. RT-DLBCL cells were treated with 1000 nM of OTX015 for eight hours. Total RNA was harvested and reverse transcribed. The resulting cDNA was utilized for quantitative PCR analysis. Expression of MYC, CDK6, and HEXIM1 was normalized to the expression of GAPDH.

**Figure S6. Ectopic overexpression of c-Myc increased % non-viable cells, and significantly increased OTX015-induced lethality in HPRT3 and HPRT2 cells. A-B.** HPRT3 and HPRT2 cells were nucleofected with empty vector or a MYC cDNA overexpression vector and incubated for 48 hours. Immunoblot analysis confirmed overexpression of c-Myc in the RT-DLBCL cells. RT-DLBCL cells were treated with the indicated concentrations of OTX015 for 48 hours and the % non-viable cells were determined by flow cytometry. Mean of two independent experiments + S. D. * = p< 0.05 as determined by two-tailed, unpaired t-test.

**Figure S7**. **Treatment with CDK9 inhibitor inhibits RNAP2 phosphorylation, depletes c-Myc, MCL1, and Bcl-xL expression and induces cell death in RT-DLBCL cells**. **A-B**. HPRT1, HPRT2, and HPRT3 cells were treated with the indicated concentrations of CDK9 inhibitor, NVP-2 or AZD4573 for 48 hours. At the end of treatment, cells were washed with 1X PBS and stained with propidium iodide. The % of PI-positive, non-viable cells were determined by flow cytometry. Mean of 2 experiments + S. D. **C-D**. HPRT2, HPRT3, and HPRT1 cells were treated with the indicated concentrations of NVP-2 for 18 hours. Cells were harvested and total cell lysates were prepared. Immunoblot analyses were conducted on the total cell lysates. The expression levels of β-Actin in the lysates served as the loading control.

**Figure S8. Treatment with BET-PROTAC induces marked transcriptional alterations in RT-DLBCL cells. A-C.** HPRT3, HPRT2, and HPRT1 cells were treated with 250 nM of ARV-771 for eight hours. Total RNA was harvested and utilized for mRNA-Seq analysis. The heat map shows the number of induced and depleted genes with a fold change (fc) of >1.5 and a p-value < 0.05. **D-F**. Venn diagrams comparing the transcriptional alterations due to treatment with BET inhibitor OTX015 versus BET-PROTAC ARV-771 in HPRT3, HPRT2, and HPRT1 cells.

**Table S10. Sequences of oligonucleotide primers (5’ to 3’) and TaqMan probe identifiers utilized in these studies.**

**Table S11. Dose and Fraction tables for combinations of OTX015 or ARV-771 with ibrutinib or venetoclax in HPRT3, HPRT2, and HPRT1 cells.**

**Table S12. ChIP-Seq, ATAC-Seq and RNA-Seq sample details and sequencing reads. A**. Sample name, ChIP antibody and total number of sequencing reads for the 3 RT-DLBCL cells. **B**. Sample name and total number of sequencing reads for the ATAC-Seq performed on the 3 RT-DLBCL cells. **C**. Sample name and total number of sequencing reads for the RNA-Seq analysis performed on the three RT-DLBCL cells treated with OTX015 or ARV-771 for 8 hours.
