# Supplementary material for: BET proteolysis targeted chimera-based therapy of novel models of Richter Transformation-diffuse large B-cell lymphoma
Source: Leukemia. 2021 Mar 2;35(9):2621–34. doi: 10.1038/s41375-021-01181-w (PMC8410602; doi:10.1038/s41375-021-01181-w)
Supplement: Supplementary file 3 — Supplemental Methods and Materials [file 41375_2021_1181_MOESM3_ESM.docx]

**Supplemental Methods**

**Reagents and antibodies.** OTX015, ABBV075, ibrutinib, adriamycin, and ABT-199 (venetoclax) were obtained from Selleck Chemicals (Houston, TX). A1155463, AZD5991, and lenalidomide were obtained from MedChem Express (Monmouth Junction, NJ). ARV-771 was kindly provided by Arvinas, Inc. All compounds were prepared as 10 mM stocks in 100% DMSO and frozen at -80°C in 5-10 µL aliquots to allow for single use, thus avoiding multiple freeze-thaw cycles that could result in compound decomposition and loss of activity. Anti-BRD4 (Cat# A301-985A50, RRID:AB_2631449) and anti-BRD2 (Bethyl Cat# A302-583A, RRID:AB_2034829) antibodies were obtained from Bethyl Labs (Montgomery, TX). Anti-p-BTK(Y223) antibody was obtained from Abcam (Cambridge, MA). Anti-c-Myc [RRID:AB_1903938], anti-IRF4 [RRID:AB_10698467], anti-cyclin D1 [RRID:AB_2070400], anti-XIAP [RRID:AB_2214870], anti-cIAP2 [RRID:AB_10693298], anti-HEXIM1 [RRID:AB_2797969], anti-p21 [RRID:AB_823586], anti-BTK, anti-NFkB2, anti-BCL2 [RRID:AB_626733], anti-MCL-1 [RRID:AB_2799149], anti-BAX [RRID:AB_10695870], anti-BAK [RRID:AB_2290287], anti-BIM [RRID:AB_1030947], anti-Bcl-xL [RRID:AB_10695729], anti-pPLCγ2, anti-p-AKT [RRID:AB_2315049], anti-AKT [RRID:AB_329827], anti-RELA, anti-IKZF1 [RRID:AB_2744523], anti-TRIM33 [RRID:AB_2798201] and anti-IKZF3 [RRID:AB_2744524] antibodies were obtained from Cell Signaling (Beverly, MA). Anti-SPOP [sc-377206] and Anti-β-Actin [RRID:AB_626630] antibodies were obtained from Santa Cruz Biotechnologies (Santa Cruz, CA). Anti-TCF4 antibody (Cat# LS‑C331289) was obtained from LSBio (Seattle, WA). Anti-CRBN, anti-DUB3 [RRID:AB_1841372], and anti-TRIM24 [RRID:AB_1843950] antibodies were obtained from Sigma-Aldrich (St Louis, MO).

**Cell lines and cell culture.** Human Richter Transformation cells harvested from the bone marrow, spleen and liver from PDX engrafted mice were cultured in RPMI media with 20% heat-inactivated fetal bovine serum (FBS), 1% penicillin/streptomycin and 1% non-essential amino acids. Following drug treatments, cells were washed free of the drug(s) prior to the performance of the studies described. Mycoplasma negative-HEK293T cells (obtained from and authenticated by the M.D. Anderson Cancer Center Characterized Cell Line Core facility) were cultured in DMEM media with 10% FBS, 1% penicillin/streptomycin, 1% glutamine, and 1% non-essential amino acids. Cells were passaged at 80% confluence by utilizing trypsin and re-plating cells in fresh DMEM media. HS5 cells [ATCC Cat#CRL-11882, RRID:CVCL_3720] were obtained and cultured in DMEM media with 10% FBS, 1% penicillin/streptomycin, 1% non-essential amino acids and 1% glutamine.

**Cell Line Authentication**. The cell lines utilized in these studies were authenticated in the Characterized Cell Line Core Facility at M.D. Anderson Cancer Center, Houston TX utilizing STR profiling.

**Primary Richter Transformation (RT) samples**. Anonymized samples of Richter Transformation DLBCL (collected from lymph node core biopsies) were obtained from the Hematopathology bank of M.D. Anderson Cancer Center under an Institutional Review Board (IRB)-approved protocol. Core biopsy samples from RT patients were passed through a 40 µm mesh strainer (Thermo Fisher) to disaggregate the tissue and isolate RT-DLBCL cells. CD19+ RT-DLBCL cells were purified by immuno-magnetic beads conjugated with an anti-CD19 antibody (StemCell Technologies, Vancouver, British Columbia) following the manufacturer’s protocol. Positively–selected CD19+ RT-DLBCL cells were suspended in serum-free RPMI media to prepare for injection into mice.

**Establishment of Richter Transformation PDX models**. All animal studies were performed under a protocol approved by the IACUC at M.D. Anderson Cancer Center, an AAALAC-accredited institution. Female and male NOD.Cg-Prkdc^scid^ Il2rg^tm1Wjl^/SzJ (NSG) mice (IMSR Cat# JAX: 005557, RRID: IMSR_JAX:005557; 4-6 weeks of age) [Jackson Labs, Bar Harbor, ME] were exposed to 2.5 Gy of radiation from a cesium source. Patient-derived (PD) RT-DLBCL cells were injected into the lateral tail vein of pre-irradiated NSG mice and monitored for engraftment. Mice that became hunched in appearance, moribund or experienced hind limb paralysis were humanely euthanized according to the approved IACUC protocol. Veterinarians and veterinary staff assisting in determining when euthanasia was required were blinded to the experimental conditions of the study. The spleen, liver, and bone marrow were collected from the mice and passed through a 40 µm mesh strainer (Thermo Fisher) to disaggregate the tissue and isolate RT-DLBCL cells. Mononuclear cells were purified by Ficoll-Hypaque density centrifugation at 3000 rpm for 10 minutes in a swinging bucket centrifuge. Mononuclear cells were washed with complete RPMI-1640 media containing 20% FBS. Human CD19+ cells were purified by immuno-magnetic beads conjugated with anti-CD19 antibody (StemCell Technologies, Vancouver, British Columbia) prior to utilization in the cell viability assays and immunoblot analyses. At each passage following the initial engraftment of RT-DLBCL cells in NSG mice, isolated RT-DLBCL cells were purified and utilized for in vitro studies as well as for passaging the PDX cells into new mice. The PDX cells were also tested by a targeted NGS panel to compare back to the original RT tumor cells. To allow for bioluminescent imaging, RT-DLBCL cells were transduced with pHIV-Luc-ZsGreen-containing lentivirus 24 hours prior to injection into NSG mice. Luciferase-ZSGreen-expressing cells were enriched by flow sorting for ZsGreen-bright cells and subsequently engrafted into additional NSG mice.

**Lentivirus production.** To generate Luciferase-ZSGreen expressing cells, pHIV-Luc-ZsGreen vector (a gift from Bryan Welm [Addgene plasmid #39196; http://n2t.net/addgene:39196; RRID: Addgene_39196]) was combined with packaging plasmids psPAX2 and pMD2.G into HEK293T. The psPAX2 and pMD2.G packaging plasmids were a gift from Didier Trono (Addgene plasmid #12260 and 12259 [RRID: Addgene_12260; RRID: Addgene_12259]). A 3:1 ratio of DNA to PEI (1 µg/µL) was employed in the transfection. Media was changed the following day. Viral supernatant was collected 72 hours post-transfection and filtered through a 0.45 µm PES membrane. RT-DLBCL cells were seeded at 1 x 10^6^ cells/mL in a 50:50 mix of media and lentiviral supernatant with 8 µg/mL polybrene (Sigma-Aldrich, St Louis, MO). The following day, the viral supernatant was removed by centrifugation and cells were transduced with fresh viral supernatant.

**Cell cycle analysis of RT-DLBCL cells.** After the designated treatments, cells were harvested by centrifuging at 125 x g for 5 minutes. Cells were washed twice with 1× phosphate-buffered saline (PBS) in 12 x 75 mm flow tubes, resuspended in 200 µL of 1X PBS and fixed in 70% ethanol by adding 800 µL of molecular grade 70% ethanol dropwise to the cells in the tube. The tubes were then vortexed to mix and stored overnight at -20°C. Fixed cells were washed twice with 1× PBS by centrifuging at 125 x g for 5 minutes and then stained in 250 µL of DNA staining buffer [5 mL Triton-PBS (100 µL of Triton X-100 in 100 mL of 1X PBS) with 100 µL of 1 mg/mL propidium iodide (Sigma Aldrich) and 100 µL of 10mg/mL RNAse A] in the dark for 15 minutes at 37°C. Cell-cycle data were collected on a flow cytometer with a 488 nM laser in the FL2 channel and analyzed with BD-Accuri CFlow6 software (BD Biosciences).

**Flow cytometry analysis of cell surface markers on RT-DLBCL cells.** To determine the immuno-phenotype of the RT-DLBCL cells, HPRT3, HPRT2, and HPRT1 cells were harvested from NSG mice. Human RT-DLBCL cells were separated from mouse splenocytes using a human/mouse chimera kit (Catalog #19849) from Stem Cell Technologies (Cambridge, MA). Human-sorted RT-DLBCL cells were washed with 1X PBS and centrifuged at 200 x g for 5 minutes. Cells were suspended in 100 µL of 0.5% BSA/PBS and stained with fluorophore-conjugated anti-CD19, anti-CD5, anti-CD10, anti-CD20, anti-CD23, anti-PD-1 or IgG-isotype controls (BD Biosciences, San Jose, CA) for 15 minutes at 4°C. Cells were washed with 200 µL of 0.5% BSA/PBS and centrifuged at 200 x g for 5 minutes. RT-DLBCL cells were suspended in 200 µL of 0.5% BSA/PBS, placed on ice, and the expression of cell surface markers was determined by flow cytometry. Percent expression of each cell surface marker is reported relative to the respective IgG isotype control.

**Immunohistochemistry analysis of RT-DLBCL cells.** Immunohistochemistry stains were performed on 4 µm-thick formalin-fixed paraffin-embedded (FFPE) tissue sections using automated Leica Bond immunostainers (Leica Biosystems, Buffalo Grove, Illinois). De-paraffinization, antigen retrieval, and staining were performed as described previously. (1, 2) IHC-certified anti-CD19, anti-CD20, anti-BCL6, anti-Ki-67, anti-MUM1/IRF4, and anti-p53 were obtained from Dako (Carpinteria, CA). Anti-CD10 and anti-BCL2 were obtained from Leica Microsystems (Novocastra) (Buffalo Grove, IL). Anti-CD5 was obtained from Thermo Fisher (Fremont, CA), anti-cMyc from Ventana Medical Systems (Tucson, AZ), and anti-PAX5 from BD Biosciences (San Jose, CA).

**FISH analysis.** Fluorescence in situ hybridization (FISH) was performed as described previously. (3) An LSI *MYC* dual‐color break apart probe set (Abbott Laboratories) was used to assess rearrangements involving the *MYC* locus on chromosome 8q24. Signals from at least 200 nuclei were analyzed.

**Karyotyping analysis.** Cytogenetic analysis was performed on G-banded metaphase cells prepared from unstimulated 24-hour and 48-hour cultured samples as described previously (4). A total of 20 metaphases (10 from each culture) were analyzed.

**Array CGH analysis.** DNA was extracted and digested, labeled, and subjected to competitive hybridization versus a reference sample on an oligonucleotide genomic array targeting cancer genes per hg-19 (4 x 180K format) (3). The presence of genomic losses/gains was determined on a genome-wide basis with an analytical sensitivity (lower limit of detection) of 1 in 5 aberration-containing cells and an average resolution of 25 kb.

**Low pass whole genome sequencing of RT-DLBCL cells for detection of TCF4 amplification.** To assess RT-DLBCL cells for TCF4 copy gains, we performed low-pass whole genome sequencing according to the previously described method. (5) Regions of amplification and loss were visualized with the Integrated Genomics Viewer (IGV).

**Next Generation Sequencing (NGS) of RT-DLBCL cells by L-300 liquid panel.** Total genomic DNA was isolated from RT-DLBCL cells (HPRT1, HPRT2 and HPRT3) and submitted to the Sheikh Khalifa Bin Zayed Al Nahyan Institute for Personalized Cancer Therapy (IPCT) at the M.D. Anderson Cancer Center for targeted NGS analysis. Genomic DNA was quantified by Picogreen (Invitrogen) and quality was accessed using Genomic DNA Tape for the 2200 Tapestation (Agilent). DNA from each sample (200-500 ng of genomic DNA) was sheared by sonication with the following conditions: Peak Incident Power 175, Duty Cycle 20%, Intensity 5, Cycles per Burst 200, and 120 seconds using Covaris E220 instrument (Covaris). To ensure the proper fragment size, samples were checked on TapeStation using the DNA High Sensitivity kit (Agilent). The sheared DNA proceeded to library prep using KAPA library prep kit (KAPA) following the “with beads” manufacturer protocol. Briefly, this protocol consists of 3 enzymatic reactions for end repair, A-tailing and adaptor ligation, followed by barcode insertion by PCR using KAPA HiFi polymerase (6 cycles). PCR primers were removed by using 1.8x volume of Agencourt AMPure PCR Purification kit (Agencourt Bioscience Corporation). At the end of the library prep, samples were analyzed on TapeStation to verify correct fragment size and to ensure the absence of extra bands. Samples were quantified using KAPA qPCR quantification kit. Equimolar amounts of DNA were pooled for capture (8-16 samples per pool). 303 genes that are clinically relevant in cancer were selected for capture. Global copy number selected areas were also captured. We designed biotin-labeled probes with Roche Nimblegen for capturing target regions (all exons in those 303 genes plus copy number regions) and followed the manufacture’s protocol for the capture step. Briefly, DNA was pooled (8-16 samples), dried out and after addition of the capture reagents and probes, samples were incubated at 47ºC on thermocycler with heated lid (57ºC) for 64-74 hours. The targeted regions were recovered using streptavidin beads and the streptavidin-biotin-probe-target complex was washed and another round of PCR amplification was performed according to manufacturer’s protocol. The quality of each captured sample was analyzed on TapeStation using the DNA High Sensitivity kit and the enrichment was accessed by qPCR using specific primers designed by Roche Nimblegen. The cutoff for the enrichment was a 50-fold minimum. The captured libraries were sequenced 2 × 100 paired end reads with a 7-nt read for indexes using Cycle Sequencing v3 reagents on a HiSeq 4000 [Illumina HiSeq 3000/HiSeq 4000 System RRID:SCR_016386] (Illumina Inc., San Diego, CA, USA) on a version 3 TruSeq paired-end flow-cell according to manufacturer’s instructions at a cluster density between 700 – 1000 K clusters/mm2. The resulting BCL files containing the sequence data were converted into “.fastq.gz” files and individual libraries within the samples were de-multiplexed using CASAVA 1.8.2 [CASAVA, RRID:SCR_001802] with no mismatches. All regions were covered by >20 reads. For data analysis, we aligned the L300 target-capture deep-sequencing data to human reference assembly hg19 using BWA [ BWA, RRID:SCR_010910] (6) and removed duplicated reads using Picard [Picard, RRID:SCR_006525] (7). We called single nucleotide variants (SNVs) and small indels using an in-house developed analysis pipeline (8), which classified variants into 3 categories: somatic, germline, and loss of heterozygosity based on variant allele frequencies in the tumor and the matched normal tissues. We called copy number alterations using a previously published algorithm, which reports gain or loss status of each exon (9). To understand the potential functional consequence of detected variants, we compared them with dbSNP [dbSNP, RRID:SCR_002338], COSMIC (10), and TCGA databases, and annotated them using VEP (11), Annovar [ANNOVAR, RRID:SCR_012821] (12), CanDrA (13) and other programs. The mutations identified in the RT-DLBCL PDX cells were compared back to the NGS panels conducted on the original tumor sample from the patient.

**Analysis of epigenetic state in RT-DLBCL cells**. ATAC-Seq analysis of RT-DLBCL cells was performed following a previously described protocol (14) with modifications on the number of amplification cycles utilized for final library preparation_._ Libraries were generated with a Nextera DNA Library Preparation Kit containing the mutant Tn5 transposase (Illumina, San Diego, CA; Catalog number: FC-121-1030). The DNA fragments were indexed utilizing a Nextera Index Kit (Illumina, San Diego, CA; Catalog number: FC-121-1011) and amplified by PCR utilizing NEBNext 2.5X PCR master mix according to the manufacturer’s protocol (New England Biolabs, Ipswich, MA). Library fragments were amplified for 12-15 cycles utilizing the denaturation, annealing, and extension times as previously described (14). The amplified library fragments were PCR-purified with a Qiagen MinElute column (Qiagen, Germantown, MD) then size selected with a 1.0X bead concentration to remove fragments shorter than 200 bp. Library fragments were incubated with AMPure XP SPRI beads (Beckman Coulter, Indianapolis, IN) for 10 minutes at room temperature in 1.5 mL microcentrifuge tubes. The mixture was placed on a magnetic stand for 10 minutes. The supernatant was removed and the SPRI beads were washed twice with fresh 80% ethanol (30 seconds each wash) and air-dried for 2-3 minutes. Library DNA was eluted from the SPRI beads with a 20 µL volume of 10 mM Tris-HCl (pH 8.5). Beads were incubated at room temperature for 10 minutes then the tubes were transferred to a magnetic stand for 10 minutes. The supernatant containing the DNA libraries was carefully removed by pipetting and transferred into a clean microcentrifuge tube. The individual libraries were quantified and quality-checked by Qubit fluorometric [Thermo Fisher Qubit fluorimeter, RRID:SCR_018095] quantification and Agilent 2100 Bioanalyzer [2100 Bioanalyzer Instrument, RRID:SCR_018043] analysis, respectively. Individual libraries were pooled at equivalent DNA amounts into one tube and purified over a Qiagen MinElute column. Pooled library DNA was eluted in 20 µL of 10 mM Tris, (pH 8.5) and sequenced on a NextSeq 500 next generation sequencer [(Illumina NextSeq 500, RRID:SCR_014983] utilizing a 150 cycle mid-output kit (Illumina, San Diego, CA). Raw sequencing data was mapped using TopHat2 [TopHat, RRID:SCR_013035] (15,16) onto the human genome build UCSC hg19 (NCBI 37) and log2-fold changes were calculated with diffReps [diffReps, RRID:SCR_010873] (17). Sequence tracks were visualized with IGV software [RRID:SCR_011793] (18,19). We also determined the H3K27Ac status and BRD4 occupancy in untreated HPRT cells by ChIPmentation following a previously described protocol (20), with modifications on the concentration of AmpPure XP beads utilized for dual AmpPure XP SPRI bead selection of the final libraries. We utilized a 0.65X bead concentration for the first selection, then a 1.0X bead concentration to narrow the fragment size of the final indexed ChIP DNA library. The individual libraries were quantified and quality-checked with Qubit and Bioanalyzer analyses, as above. The libraries were pooled at equivalent DNA amounts into one tube and purified utilizing a Qiagen MinElute column. Pooled library DNA was eluted in 20 µL of 10 mM Tris, (pH 8.5) for loading onto a NextSeq 500 sequencer utilizing a mid-output kit. Raw sequence data were mapped to UCSC hg19 (NCBI 37) (as above) and log2 fold-changes were calculated with diffReps [diffReps, RRID:SCR_010873] (17). Sequence tracks were visualized with IGV software [RRID:SCR_011793] (18, 19). To identify super enhancers, we performed a rank order of super enhancers (ROSE) analysis [ROSE, RRID:SCR_017390] utilizing the H3K27Ac status of the chromatin according to the methods of Loven et al. (21) following the default settings within the algorithm. To compare H3K27Ac status in RT-DLBCL cells with that of normal CD34+ HPCs, we utilized publicly available H3K27Ac ChIP-seq datasets from GEO (GSM772870, GSM772885, and GSM772894). To compare chromatin accessibility in RT-DLBCL cells with that of normal CD34+ HPCs, we utilized publicly available ATAC-Seq data from GEO (GSE18927).

**Transcriptome Analysis.** Following the designated treatments with ARV-771 or OTX015 for 8 hours, total RNA was isolated from RT-DLBCL cells and sequencing libraries were prepared in the MD Anderson Cancer Center DNA Sequencing and Microarray core facility. Prepared cDNA libraries for mRNA-Seq were sequenced on an Illumina HiSeq 4000 [Illumina HiSeq 3000/HiSeq 4000 System, RRID:SCR_016386]. Each library yielded 30-40 million read pairs. Data was mapped using TopHat2 [TopHat, RRID:SCR_013035] (15,16) onto the human genome build UCSC hg19 (NCBI 37). Gene expression was assessed using Cufflinks2 [Cufflinks, RRID: SCR_014597] (22), then variance stabilization and quantile normalization were applied. Significantly altered transcripts were determined using the limma package (23) in R [LIMMA, RRID: SCR_010943]; multiple hypotheses testing correction was applied using the false discovery rate (fdr) method implemented in the R statistical system. We considered that significance was achieved for fold changes greater than or equal to 1.5-fold (up or down) relative to the untreated cells, and p-values less than 0.05.

**RNA isolation and quantitative polymerase chain reaction.** Following the designated treatments with ARV-771 or OTX015, total RNA was isolated from RT-DLBCL cells utilizing a PureLink RNA Mini kit from Ambion, Inc. and reverse transcribed with a High Capacity Reverse Transcription kit from Ambion Inc. Quantitative real-time PCR analysis for the expression of MYC, CDK6, and HEXIM1 was performed on cDNA using TaqMan probes from Applied Biosystems (Foster City, CA). Relative mRNA expression was normalized to the expression of GAPDH and compared to the untreated cells.

**Single-cell RNA Seq analysis of RT-DLBCL cells.** To determine baseline expression of mRNA in the RT-DLBCL cells at single cell resolution, we performed single-cell RNA Seq analysis utilizing the 10X Genomics Chromium Separator and a Chromium™ Single Cell 3’ Solution kit following the manufacturer’s protocol. Briefly, RT-DLBCL cells were washed with 2% FBS/PBS, counted on a Countess-2 cell counting instrument (Life Technologies, Carlsbad, CA) and cell concentrations were adjusted to 700-1200 cells/microliter to prepare for loading on the 10X Genomics Chromium Separator. Microfluidic partitioning on the Chromium Separator was used to capture single cells with Gel Beads in emulsion (GEMs) containing 10X barcodes, unique molecular identifier sequence and poly(dT) primer sequence. GEMs with captured cells were subjected to reverse transcription in emulsion. GEMs were then broken by addition of recovery reagent and released cDNA was utilized to prepare barcoded, next-generation sequencing (NGS) cDNA libraries following the manufacturer’s protocol. Prepared libraries were sequenced on a Hi-Seq4000 Next generation sequencer [RRID:SCR_016386] using 2 × 100 paired end reads. Sequencing files were loaded into Cell Ranger and then Loupe Cell Browser for clustering, visualization, and analysis. Marker genes that define the clusters are (q-value <0.1, FC>1.2) in 2 out of the 3 cases; MYC (HPRT1, HPRT3), MCL1 (HPRT1, HPRT3), BIRC3 (HPRT1, HPRT3), IRF4 (HPRT2, HPRT3), TCF4 (HPRT2, HPRT3). These are all well-characterized BRD4 target genes, and are coordinately down-regulated following BET inhibition/degradation.

**Assessment of percentage non-viable cells.** Following designated treatments, RT-DLBCL cells were stained with trypan blue (Sigma, St. Louis, MO) and counted on a Countess-2 cell counting instrument (Life Technologies, Carlsbad, CA). Alternatively, cells were washed with 1X PBS, stained with propidium iodide or To-Pro-3 iodide (Life Technologies, Carlsbad, CA) and analyzed by flow cytometry on a BD Accuri CFlow-6 flow cytometer (BD Biosciences, San Jose, CA). To analyze synergism between ARV-771 or OTX015 and ibrutinib or ABT199, cells were treated with combinations for 48 hours and the percentage of To-Pro-3 iodide-positive, non-viable cells was determined by flow cytometry. The combination index (CI) for each drug combination was calculated by median dose effect and isobologram analyses (assuming mutual exclusivity) utilizing the commercially available software CompuSyn (24). CI values of less than 1.0 represent a synergistic interaction of the two drugs in the combination. The CI values were input into GraphPad [GraphPad Prism, RRID:SCR_002798] V8.0 to create the Box plots of the range of the CI values for each cell line and combination.

**Cell lysis and protein estimation.** Untreated or drug-treated cells were centrifuged, and the cell pellets were incubated in lysis buffer on ice for 20 minutes (25, 26). After centrifugation, an aliquot of each cell lysate was diluted 1:10 and the protein content was quantified using a BCA protein quantitation kit (Pierce, Rockford, IL), according to the manufacturer’s protocol. Protein concentrations were determined by comparing the absorbance at 562 nm compared to a known concentration range of bovine serum albumin (BSA) from 0.125 mg to 2 mg/mL on a BioTek synergy H1 plate reader (BioTek, Winooski, VT).

**SDS-PAGE and immunoblot analyses.** Seventy five micrograms of total cell lysate were used for SDS-PAGE. Western blot analyses of BRD4, BRD2, c-Myc, CDK4, HEXIM1, XIAP, Bcl-xL, p-BTK(Y223), BTK, Cyclin D1, MCL1 and cIAP2 were performed on total cell lysates using specific antisera or monoclonal antibodies. Blots were washed with 1X PBST, then incubated in IRDye 680 goat anti-mouse [ Cat# 925-68070, RRID:AB_2651128] or IRDye 800 goat anti-rabbit [Cat# 926-32211, RRID:AB_621843] secondary antibodies (LI-COR, Lincoln, NE) for 1 hour, washed 3 times in 1X PBST and scanned with an Odyssey CLX Infrared Imaging System (LI-COR, Lincoln, NE). The expression levels of β-Actin or GAPDH in the cell lysates were used as the loading control for the Western blots. Immunoblot analyses were performed at least twice and representative immunoblots are shown.

**CRISPR/Cas9-mediated gene editing in RT-DLBCL cells.** To study the effects of knockout of IRF4 in RT-DLBCL cells, the CHOP-CHOP [CHOPCHOP, RRID:SCR_015723] prediction algorithm (27) was utilized to develop guide RNAs. sgRNAs were synthesized by Synthego, Inc. To obtain Cas9-sgRNA RNPs (ribonucleoprotein complexes), 1 μg of synthetic sgRNA was incubated with 1.5 μg Cas9 for 15 min at room temperature. 7 x 10^6^ HPRT2 or HPRT3 cells were transfected by electroporation utilizing the Amaxa Nucleofector device and a CD34+ nucleofector kit, following the manufacturer’s protocol (U-08) (Amaxa GmbH, Cologne, Germany). Immediately post-transfection, cells were plated on a GFP-expressing HS5 stromal cell monolayer and incubated for 5 days. Knockout of IRF4 was confirmed by confocal immunofluorescent microscopy 5-6 days post-transfection. Gene-edited cells were also treated with OTX015 for 48 hours, and the percentage of To-Pro-3 iodide-positive, non-viable cells were determined by flow cytometry.

**Confocal immunofluorescence microscopy.** Following drug treatments or sgRNA transfection, HPRT cells were cytospun onto glass slides to prepare for confocal microscopy. Cells were fixed with 4% paraformaldehyde in 1X PBS for 10 minutes, then washed three times with 1X PBS. Next, cells were permeabilized with 0.5% Triton X-100 for 5 minutes, then rinsed three times with 1X PBS. HPRT cell containing slides were blocked in 3% BSA/PBS + 1% FBS for 30 minutes at room temperature. Next, primary antibody for IRF4 or c-Myc (mouse monoclonal) was added and the cells/slides were incubated in a humidified chamber for 2-3 hours. Excess antibody was removed by washing the slides with 1X PBS for 5 minutes. Anti-mouse or anti-rabbit Alexa 488- or Alexa 594-conjugated secondary antibodies (Invitrogen, Carlsbad, CA) were added and the slides were incubated in a humidified chamber for 1 hour in the dark. Excess antibody was removed by washing three times with 1X PBS. Nuclei were counterstained with DAPI (#62248, ThermoFisher), and then coverslips were mounted onto the slides utilizing Prolong Diamond anti-fade mountant (P36970, LifeTech, Carlsbad, CA). Imaging was performed on a Zeiss Confocal microscope with a 60X objective in the MD Anderson Flow Cytometry and Imaging Core. Representative images of IRF4 and c-Myc in the sg-Negative- or sgIRF4 transfected cells are shown for each condition.

**Image Quantification**. Mean fluorescence intensity analysis of confocal images and Western blot densitometry analysis was performed using ImageJ [ImageJ, RRID:SCR_003070] software (28). Twenty-five to fifty cells per condition were used for quantitative analysis of mean fluorescence intensity of confocal images. Violin plots were generated in and significance was calculated with GraphPad [GraphPad Prism, RRID:SCR_002798] V8 software.

**Ectopic expression of c-Myc in RT-DLBCL cells.** For ectopic overexpression of c-Myc in RT-DLBCL cells, HPRT3 and HPRT2 cells (5-7 x 10^6^ cells per condition) were centrifuged at 200 x g for 5 minutes, washed with 1X PBS, then suspended in 100 µL of P3 primary cell solution (Lonza Catalog number PBP3-02250) and combined with 3 µg of pCDH-puro-Myc vector. pCDH-puro-cMyc was a gift from Jialiang Wang (Addgene plasmid #46970; http://n2t.net/addgene:46970; RRID:Addgene_46970). Cells were nucleofected with program EO-117 on a 4D nucleofector device (Lonza). Cells were immediately transferred to complete media and plated on a GFP-expressing HS5 stromal cell monolayer and incubated at 37°C for 48 hours. Cells were harvested for immunoblot analysis or treated with BET inhibitor for determining the effects on cell viability.

**Power analysis for in vivo studies**. With a sample size of 6 mice per group, we can achieve 80% power to detect a difference of overall survival at a significance level of 0.05 with log-rank test, assuming a signal to noise ratio of 1.8 and a 30% mouse-survival at the end of study in the experimental groups.

**RT-DLBCL xenograft models.** All animal studies were performed under a protocol approved by the IACUC at M.D. Anderson Cancer Center, an AAALAC-accredited institution. To assess the in vivo activity of BET-PROTAC ARV-771 and/or venetoclax, 3 x 10^6^ HPRT3 cells were injected into the lateral tail vein of female NOD.Cg-Prkdc^scid^ Il2rg^tm1Wjl^/SzJ (NSG) mice (stock number: 005557; 4-6 weeks of age) [Jackson Labs, Bar Harbor, ME; RRID: IMSR_JAX:005557] (n=6 per cohort) which had received a pre-conditioning dose of radiation (2.5 gray) 24 hours prior to injection of cells. Mice were monitored daily for signs or symptoms of disease. Anti-CD45 antibody staining and flow cytometry was used to document engraftment (> 1.0% CD45+ cells in the peripheral blood of mice) of HPRT3 cells in the mice before treatment was initiated. Mice were randomized into 4 cohorts and treated with vehicle, 20 mg/kg of ARV-771 (subcutaneous injection, daily x 5 days per week) or 15 mg/kg of venetoclax (by oral gavage, daily x 5 days per week) for 3 weeks. Mice that became moribund or experienced hind limb paralysis were euthanized according to the approved IACUC protocol. Veterinary staff assisting in determining when euthanasia was required were blinded to the experimental conditions of the study. The survival of the mice is represented by a Kaplan Meier plot. A Mantel–Cox Rank sum test was utilized for group comparisons. P-values of < 0.05 were assigned significance A separate cohort of mice was injected and monitored as above. Mice were then treated for 3 weeks with vehicle, ARV-771, venetoclax, or the combination, as above. To document effects of treatment on spleen and liver volume mice were anesthetized with 2% isoflurane and then oxygen (0.5%) and isoflurane (2%) were administered to keep the mice anesthetized during imaging. The liver and spleen in the mice were imaged utilizing a T2-weighted axial scan performed by small animal imaging facility (SAIF) staff on a Biospec USR70/30 magnetic resonance imaging (MRI) instrument (Bruker Biospin MRI, Billerica, MA). Mice were also sacrificed for correlative analysis of spleen length and spleen mass. Spleen and liver volume were quantified from MRI images by ImageJ [RRID: SCR_003070] and graphed with GraphPad V8 [GraphPad Prism, RRID:SCR_002798].

**Statistical analysis**. Significant differences between values obtained in HPRT3, HPRT2, or HPRT1 cells treated with different experimental conditions were determined using the Student’s t-test in GraphPad V8 [GraphPad Prism, RRID:SCR_002798]. For the *in vivo* mouse models, a two-tailed t-test or a Mantel–Cox Rank sum test was utilized for group comparisons. P values of < 0.05 were assigned significance.

**Data and Software availability**. RNA-Seq, ATAC-Seq and ChIP-Seq datasets have been deposited in GEO as a Super Series under accession # GSE154463. Sample names and total sequencing reads for each sample are also provided in Supplemental Table S12.

**REFERENCES for Supplemental Methods**

1. Khoury JD, Wang WL, Prieto VG, Medeiros LJ, Kalhor N, Hameed M, Broaddus R, Hamilton SR. Validation of Immunohistochemical Assays for Integral Biomarkers in the NCI-MATCH EAY131 Clinical Trial. Clin Cancer Res. 2018; 24:521-531.

2. Sukswai N, Khoury JD. Immunohistochemistry Innovations for Diagnosis and Tissue-Based Biomarker Detection. Curr Hematol Malig Rep. 2019; 14: 368-375.

3. Tang Z, Li Y, Wang W, Yin CC, Tang G, Aung PP, Hu S, Lu X, Toruner GA, Medeiros LJ, Khoury JD. Genomic aberrations involving 12p/ETV6 are highly prevalent in blastic plasmacytoid dendritic cell neoplasms and might represent early clonal events. Leuk Res. 2018; 73: 86-94.

4. Khoury JD, Sen F, Abruzzo LV, Hayes K, Glassman A, Medeiros LJ. Cytogenetic findings in blastoid mantle cell lymphoma. Hum Pathol. 2003; 34: 1022-9.

5. Dong Z, Xie W, Chen H, Xu J, Wang H, Li Y, Wang J, Chen F, Choy KW, Jiang H. Copy-Number Variants Detection by Low-Pass Whole-Genome Sequencing. Curr Protoc Hum Genet. 2017; 94:8.17.

6. Li H, Durbin R: Fast and accurate short read alignment with Burrows-Wheeler transform. Bioinformatics 2009, 25:1754-1760.

7. DePristo MA, Banks E, Poplin R, Garimella KV, Maguire JR, Hartl C, Philippakis AA, del Angel G, Rivas MA, Hanna M, et al: A framework for variation discovery and genotyping using next-generation DNA sequencing data. Nat Genetics 2011, 43:491-498.

8. Zhou W, Chen T, Zhao H, Eterovic AK, Meric-Bernstam F, Mills GB, Chen K: Bias from removing read duplication in ultra-deep sequencing experiments. Bioinformatics 2014, 30: 1073-80.

9. Lonigro RJ, Grasso CS, Robinson DR, Jing X, Wu YM, Cao X, Quist MJ, Tomlins SA, Pienta KJ, Chinnaiyan AM: Detection of somatic copy number alterations in cancer using targeted exome capture sequencing. Neoplasia 2011, 13:1019-1025.

10. Bamford S, Dawson E, Forbes S, Clements J, Pettett R, Dogan A, Flanagan A, Teague J, Futreal PA, Stratton MR, Wooster R: The COSMIC (Catalogue of Somatic Mutations in Cancer) database and website. Brit J Cancer 2004, 91:355-358.

11. McLaren W, Pritchard B, Rios D, Chen Y, Flicek P, Cunningham F: Deriving the consequences of genomic variants with the Ensembl API and SNP Effect Predictor. Bioinformatics 2010, 26:2069-2070.

12. Wang K, Li M, Hakonarson H: ANNOVAR: functional annotation of genetic variants from high-throughput sequencing data. Nucleic Acids Res 2010, 38:e164.

13. Mao Y, Chen H, Liang H, Meric-Bernstam F, Mills GB, Chen K: CanDrA: Cancer-Specific Driver Missense Mutation Annotation with Optimized Features. PLoS One 2013, 8: e77945.

14. Buenrostro JD, Wu B, Chang HY, Greenleaf WJ. ATAC-seq: A Method for assaying chromatin accessibility genome-wide. Curr Protoc Mol Biol 2015; 109, 21.29.1-9.

15. Heinz S, Benner C, Spann N, Bertolino E, Lin YC, Laslo P et al. Simple Combinations of Lineage-Determining Transcription Factors Prime cis-Regulatory Elements Required for Macrophage and B Cell Identities. Mol Cell 2010; 38: 576-589.

16. Kim, D., Pertea, G., Trapnell, C., Pimentel, H., Kelley, R., and Salzberg, S. L. TopHat2: accurate alignment of transcriptomes in the presence of insertions, deletions and gene fusions. Genome Biol 2013; 14, R36.

17. Shen L, Shao N-Y, Liu X, Maze I, Feng J, Nestler EJ. diffReps: Detecting Differential Chromatin Modification Sites from ChIP-seq Data with Biological Replicates. PLoS ONE. 2013; 8: e65598. doi:10.1371/journal.pone.0065598.

18. Robinson JT, Thorvaldsdottir H, Winckler W, Guttman M, Lander ES, Getz G, et al. Integrative genomics viewer. Nat Biotechnol. 2011;29:24-6.

19. Thorvaldsdottir H, Robinson JT, Mesirov JP. Integrative Genomics Viewer (IGV): high-performance genomics data visualization and exploration. Briefings in bioinformatics. 2013;14:178-92.

20. Schmidl C, Rendeiro AF, Sheffield NC, Bock C. ChIPmentation: fast, robust, low-input ChIP-seq for histones and transcription factors. Nat Methods. 2015;12:963-5.

21. Loven J, Hoke HA, Lin CY, Lau A, Orlando DA, Vakoc CR, et al. Selective inhibition of tumor oncogenes by disruption of super-enhancers. Cell. 2013;153:320-34.

22. Trapnell, C., Williams, B. A., Pertea, G., Mortazavi, A., Kwan, G., van Baren, M. J., Salzberg, S. L., Wold, B. J., and Pachter, L. Transcript assembly and quantification by RNA-Seq reveals unannotated transcripts and isoform switching during cell differentiation. Nat Biotechnol. 2010; 28, 511-515.

23. Smyth, G. K. Linear models and empirical bayes methods for assessing differential expression in microarray experiments. Statistical Appl Gen Mol Biole. 2004; 3, Article3.

24. Chou TC, Talalay P. Quantitative analysis of dose-effect relationships: the combined effects of multiple drugs or enzyme inhibitors. Adv Enzyme Regul 1984; 22: 27-55.

25. Wang Y, Fiskus W, Chong DG, Buckley KM, Natarajan K, Rao R, et al. Cotreatment with panobinostat and JAK2 inhibitor TG101209 attenuates JAK2V617F levels and signaling and exerts synergistic cytotoxic effects against human myeloproliferative neoplastic cells. Blood 2009; 114: 5024-5033.

26. Fiskus W, Verstovsek S, Manshouri T, Rao R, Balusu R, Venkannagari S, et al. Heat Shock Protein 90 Inhibitor Is Synergistic with JAK2 Inhibitor and Overcomes Resistance to JAK2-TKI in Human Myeloproliferative Neoplasm Cells. Clin Cancer Res 2011; 17: 7347-7358.

27. Labun K, Montague TG, Gagnon JA, Thyme SB, Valen E. CHOPCHOP v2: a web tool for the next generation of CRISPR genome engineering. Nucleic Acids Res. 2016; 44: W272-6.

28. Schneider, C.A., Rasband, W.S., Eliceiri, K.W. NIH Image to ImageJ: 25 years of image analysis. Nat Methods 2012; 9, 671-675.
